# In silico designing of vaccine candidate against *Clostridium difficile*

**DOI:** 10.1038/s41598-021-93305-6

**Published:** 2021-07-09

**Authors:** Srijita Basak, Debashrito Deb, Utkarsh Narsaria, Tamalika Kar, Filippo Castiglione, Indraneel Sanyal, Pratap D. Bade, Anurag P. Srivastava

**Affiliations:** 1 Biopharmaceutical Development Department, Syngene International Limited, Bangalore, India; 2 Institute for Applied Computing (IAC), National Research Council of Italy, Rome, Italy

**Keywords:** Computational biology and bioinformatics, Immunology, Biochemistry

## Abstract

*Clostridium difficile* is a spore-forming gram-positive bacterium, recognized as the primary cause of antibiotic-associated nosocomial diarrhoea. *Clostridium difficile* infection (CDI) has emerged as a major health-associated infection with increased incidence and hospitalization over the years with high mortality rates. Contamination and infection occur after ingestion of vegetative spores, which germinate in the gastro-intestinal tract. The surface layer protein and flagellar proteins are responsible for the bacterial colonization while the spore coat protein, is associated with spore colonization. Both these factors are the main concern of the recurrence of CDI in hospitalized patients. In this study, the CotE, SlpA and FliC proteins are chosen to form a multivalent, multi-epitopic, chimeric vaccine candidate using the immunoinformatics approach. The overall reliability of the candidate vaccine was validated in silico and the molecular dynamics simulation verified the stability of the vaccine designed. Docking studies showed stable vaccine interactions with Toll‐Like Receptors of innate immune cells and MHC receptors. In silico codon optimization of the vaccine and its insertion in the cloning vector indicates a competent expression of the modelled vaccine in *E. coli* expression system. An in silico immune simulation system evaluated the effectiveness of the candidate vaccine to trigger a protective immune response.

## Introduction

*Clostridium difficile*, a spore forming, gram positive bacteria is the causative agent of nosocomial, antibiotic-associated severe diarrhoea^1–3^. These obligate anaerobic bacteria cause *Clostridium difficile* infection (CDI) that includes symptoms like diarrhoea with or without colitis, abdominal pain, fever with chills and discomfort^3–5^. CDI is mostly acquired and transmitted in hospitals and nursing homes, where the use of efficacious antibiotics and antimicrobials are high^6,7^. It has a mortality rate ranging from 6 to 17% and a substantial morbidity all over the world^8^. CDI also shows high recurrence rates (range 15–41%) complicating the infection. This may surge even further with every new episode with larger and more prominent impact, which has been previously discussed by Haubitz et al., and Peery et al.^9,10^**.** Likewise, Murphy et al., summarized the mortality data in National Centre for Health Statistics (NCHS) by noting, “Enterocolitis due to *Clostridium difficile*… has become a growing concern in recent years”^11^.

CDI contamination occurs after the ingestion of spores, which are shed into the environment by patients with or without disease symptoms. These spores germinate in the gastro-intestinal tract, allowing the colonization of the vegetative cells in the gut and multiplication in the colon^12^. The colonization is aided by the dysbiosis of internal gut microbiota which mostly results from excessive antibiotic treatments. The spore proteins, other than being agents for disease transmission, play a role in development of the disease. There are number of proteins present on the outermost layer of the spore coat, but CotE protein is of particular significance as it plays an important role in virulence^13^. This 81 kDa protein is bi-functional, as it carries the N-terminal peroxiredoxin domain as well as the C-terminal chitinase domain^13,14^ hence it binds to internal mucin glycoproteins enabling adhesion of the spore to the internal GI tract surface. Spore attachment is essential for CDI with the internal colonization being initiated by the CotE spore protein^15^.

The surface-layer proteins (SLP) are the most abundant cellular proteins in bacteria^16^. SLPs are capable of adherence to human gastro-intestinal and intestinal epithelial cells in vitro^17^. Studies on *C. difficile* SLP shows that they not only play major roles in survival and growth, but also interact with the host and its immune system through TLR4 activation, by inducing the production of cytokines^7,18^. SlpA protein being the most abundant protein in the S-layer, acts as a major colonization factor^19^ and recombinant vaccines developed against SlpA showed reduced gut colonization in mice^7,20^. Thus, this protein can be regarded as a possible candidate for the design of vaccines.

The flagellar proteins, FliC and FliD present in the bacteria play important roles in motility, colonization, biofilm formation and toxin gene expression^21,22^. It has been observed that the major role of bacterial cell adherence to the gut surface is played by flagellar cap protein (FliC), unlike flagellar subunit protein (FliD) which acts as a weak binder for cell adherence activity^23^. The attachment of the bacterial cell to the gut enables onset of infection. This marks the triggering of innate immune responses, through the activation of TLR5 which helps in protection against colonization^12^. FliC protein’s active interaction with TLR5 induces strong immune response in the body, which makes it a preferred choice for vaccine construction^2^.

For immunization through vaccination against highly morbid CDI, toxin antigens represent the first studied targets and anti-toxin antibodies formed after such vaccinations are concerned with preventing CDI recurrence^24^. Given that, *C. difficile*’s virulence factors also include TcdA and TcdB proteins, where efforts were made to develop *C. difficile* vaccines targeting both TcdA and TcdB. Vaccination with toxoids or recombinant fragments of toxins were tested with varying but minimal success in both animals and humans^24^. Nonetheless, *C. difficile* survives in the environment as very stable spore forms, resistant to antibiotics and harsh conditions, and is the root cause of recurrent CDI^25^. Toxoid vaccines cannot avoid the sporulation or deposition of *C. difficile* spores into the body, potentially raising the number of asymptomatic carriers of the disease. As a result, vaccine candidates that prevent colonization and adhesion of bacteria to gut epithelia are currently under consideration^26^. A vaccine strategy that tackles single or multiple factors responsible for colonization, adherence and persistence has a considerable advantage over other developing vaccines. Hence, other proteins like CotE spore protein, SlpA S-layer protein and FliC flagellar cap protein should be considered preferred vaccine candidates over conventional toxoid vaccines for the prevention of *C. difficile* infections.

**Figure 1:**
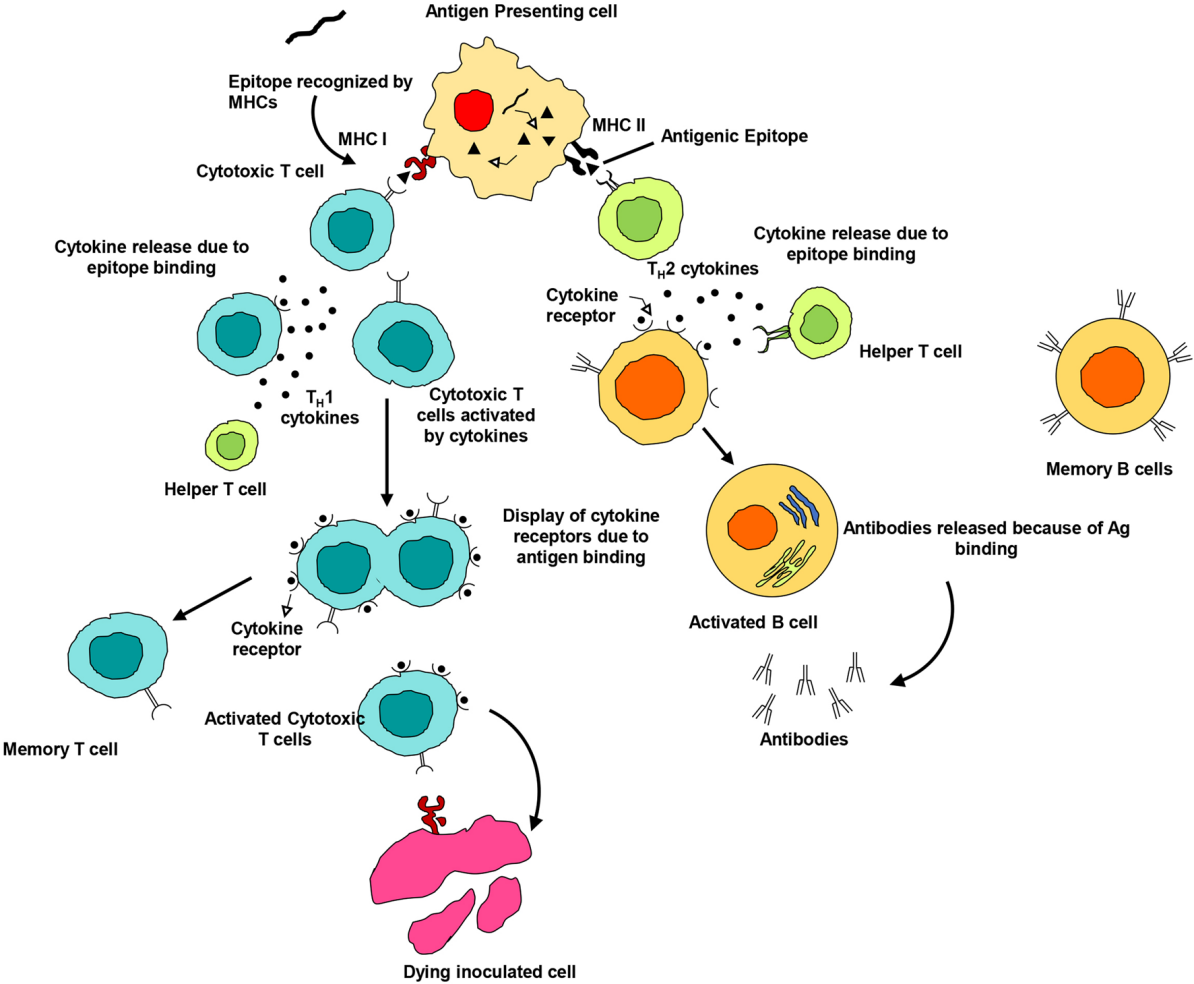
Immune mechanism of action elicited by the vaccine construct.

Upon injecting into the body, the vaccine candidate is identified by the innate immune system of the host through pattern recognition receptors (PRRs) that bind pathogen-associated molecular patterns (PAMPs). The host cells sense the possible danger when they detect a pathogen through PAMPs embedded in the vaccine and activate dendritic cells, monocytes and neutrophils that patrol the whole body^27^. The activated immune cells (dendritic cells and monocytes) after sensing ‘danger signals’ elicited by the vaccine, modulate the expression of surface molecules and secrete pro-inflammatory cytokines and chemokines^28^. Matured dendritic cells formed due to an inflammatory microenvironment, migrate towards lymph nodes where T and B lymphocytes activate^29^. At around the same time, antigen presenting cells process the antigens into smaller fragments that are then displayed in the grooves of MHC (called Human Leukocyte Antigens, HLA, in humans) molecules on the cell surface. MHC class I molecules bind to antigen peptides produced in the cytosol of infected cells, whereas phagocytized antigens are primarily displayed on MHC class II molecules^30–32^. CD4 + T cells participate in the identification of antigenic peptides, which are displayed by MHC class II molecules, whereas after activation, CD8 + T cells binds to MHC class I- peptide^33^. Furthermore, the secreted cytokines by the activated CD4 + T cells helps in activation of B cells for proper antibody generation^34^ (Fig. 1).

Vaccine design which involves either the entire organism or large proteins results in unnecessary antigenic load and chances of allergenic responses^35^. This difficulty in vaccine development can be overcome by peptide-based vaccines, which involves short immunogenic peptide fragments^35^. These short peptides are meant to be sufficient in eliciting strong immune responses, while avoiding the induction of allergenic responses. Recent developments in computational biology have opened new opportunities in the design of successful vaccines in silico^36,37^. Vaccine models designed by this approach have been tested for their efficacy by injecting them into mice models^38,39^. The results of these experimentations have assured positive immune responses in the animal models, which promises its competency as fast-developing alternative for vaccine designing. In this study, the in silico approach has been used for designing a chimeric vaccine consisting of CotE, SlpA and FliC proteins to prevent *Clostridium difficile* infection. The designed vaccine candidate induces the cytotoxic T cell and helper T cell activation, along with the ability to induce interferon-γ (IFN-γ), interleukin-2 (IL-2) as well as other pro-inflammatory cytokines (like TNF, IL-18, IL-12, etc.).

## Results

### T cell epitopes and IFN-γ epitope prediction

**Figure 2:**
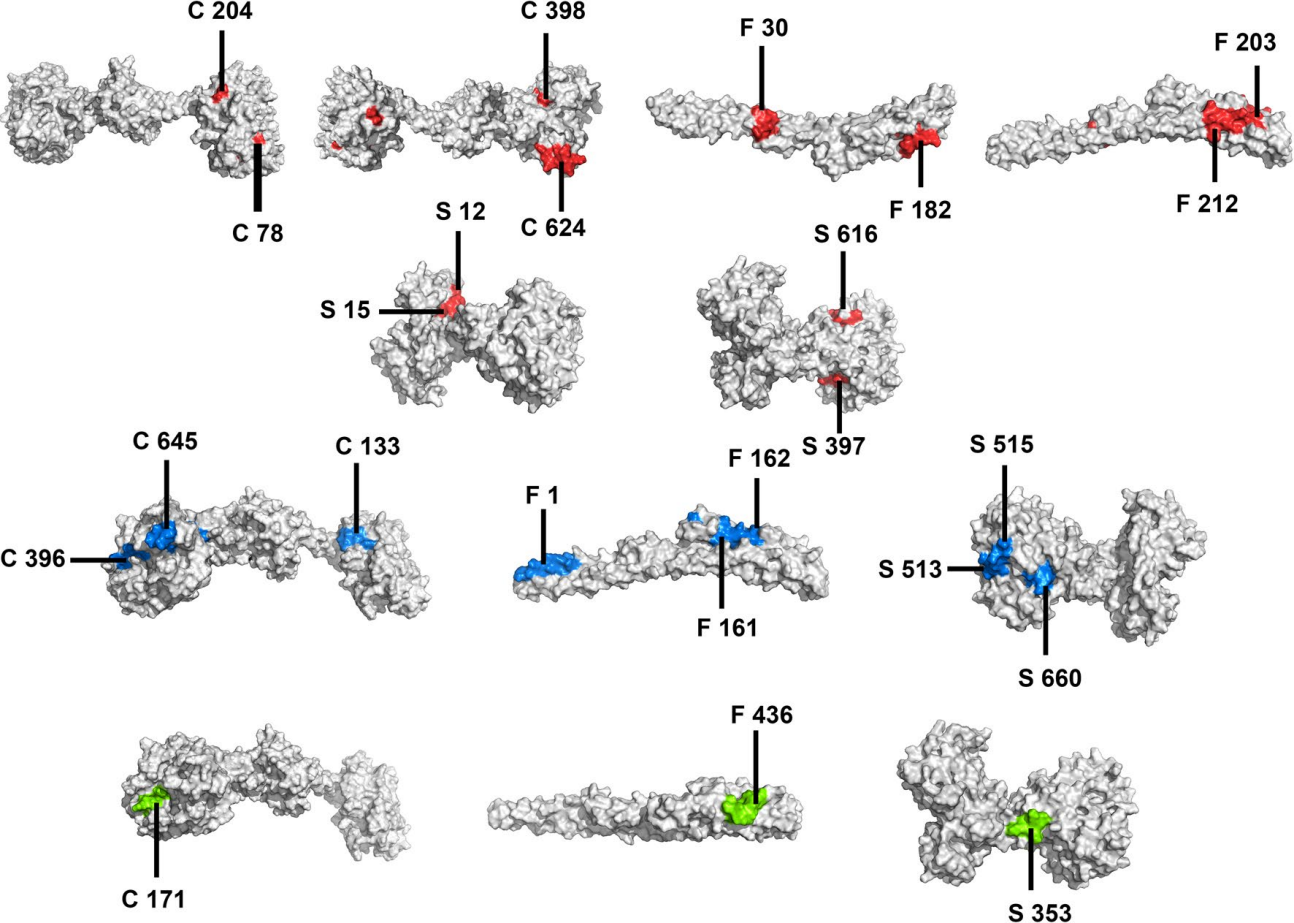
3D structure of the CotE, FliC and SlpA proteins showing surface positions of CTL epitopes in red colour, HTL epitopes in blue colour and IFN-γ epitopes in green colour, where C denotes CotE, F denotes FliC and S denotes SlpA.

A significant role is played by the cytotoxic T lymphocytes (CTL) and helper T lymphocytes (HTL) in generation of a long-lasting adaptive immunity against various microbial infections. Long lasting cellular immunity, generated by CTL epitopes, has the ability of eliminating the pathogen infected cells as well as the circulating antigen in the body^40^. In contrary to the CTL epitopes, HTL epitopes are necessary for the generation of both humoral and cell mediated immunity^41^. In fact, HTL epitopes are essential for the development of memory helper T cells which are pivotal to both the activation of cytotoxic T cells and stimulation of B lymphocytes responsible for producing antibodies^42^. Hence, consideration of CTL and HTL epitopes is preferred in an efficient vaccine candidate design which has the ability to elicit antibodies and cytotoxicity. The CTL epitopes from the selected proteins were predicted using the server NetCTL 1.2 (Table 1) (Supplementary Tables S1, S2 and S3). For the epitope prediction, this server uses an algorithm, which considers a combined score of MHC class I binding, proteasomal C terminal cleavage and TAP transport efficiency^43^. IEDB consensus method was used to screen the epitopes which are good binders to alleles^44^. The HTL epitopes were predicted using NetMHC II pan 3.2 server (Table 2) (Supplementary Tables S4, S5 and S6). The selected epitopes were further subjected to filters like antigenicity and immunogenicity where the antigenicity and immunogenicity of the epitopes were checked by VaxiJen v2.0 and IEDB class I immunogenicity servers, respectively. The final epitopes which are included in the vaccine construct were visualized on the modelled 3D structures of the SlpA, CotE and FliC proteins, as shown in Fig. 2.

**Table 1:** CTL epitopes predicted using NetCTL 1.2 server. Position indicates the starting residue of the predicted 9-mer. 9-mer peptides with prediction score greater than 0.75 were considered as epitopes which has a good probability to bind with the MHC class-I alleles. In addition, epitopes with lower binding score having Ic50 < 500 nm are considered as good binders, where the Ic50 value indicates how strongly an epitope can bind to a particular allele. Epitopes with antigenicity score > 0.4 are considered to be antigenic which have the possibility of generating an effective immune response.

CTL EPITOPES								
Protein	Epitopes	Supertype	Position	MHC class-I allele	Binding score	Ic50	Prediction score	Antigenic score
CotE	NSSHLAWMY	A1, A26, B58	78	HLA-A*01:01	0.13	20.05	3.5343	0.4680
				HLA-A*30:02	0.81	89.8		
	LLDAVIFAF	A1,A2, B58, B62	398	HLA-A*02:06	1.4	132.01	1.1808	1.1032
				HLA-A*32:01	1.4	231.86		
				HLA-A*02:01	2.0	461.74		
	SCMDWYLCF	A24, A26, B62	204	HLA-A*24:02	0.505	135.1	0.7504	0.9475
				HLA-A*23:01	0.865	195.5		
	YINKNGYEY	A1, A26, B62	624	HLA-A*30:02	0.525	123.44	1.8236	0.6808
				HLA-A*01:01	0.785	416.33		
				HLA-B*35:01	1.0	12.93		
				HLA-B*15:01	1.1	105.36		
SlpA	AATTGTQGY	A1, A26, B62	24	HLAA*30:02	1.5	379.91	0.9743	1.7427
				HLA-B*35:01	2.0	126.52		
	TAIELSSKY	A1, A26, B62, B58	397	HLA-A*26:01	0.22	32.47	0.9237	0.5584
				HLA-B*35:01	0.5	19.93		
	VLASAAPVF	A24, B58, B62	15	HLA-B*15:01	0.4	17.47	0.8543	0.4683
				HLA-A*24:02	1.515	323.22		
				HLA-A*23:01	1.95	927.42		
	RQATNAEVL	B27, B39, B44, B62	616	HLA-B*40:01	0.725	106.84	1.1875	0.9416
				HLA-B*15:01	1.1	137.3		
FliC	MVSSLDAAL	B7, B8, B62,	203	HLA-A*68:02	0.14	14.27	0.8390	0.6392
				HLA-B*35:01	0.6	18.18		
				HLA-B*53:01	2.0	968.62		
				HLA-B*07:02	2.0	959.49		
	TTASIGSMK	A1, A3	182	HLA-A*68:01	0.11	5.21	1.3955	0.5482
				HLA-A*11:01	0.245	17.24		
				HLA-A*03:01	0.39	66.19		
	LSSGVRIKR	A3	30	HLA-A*68:01	0.63	29.82	0.8417	1.0298
				HLA-A*31:01	1.035	44.88		
				HLA-A*33:01	1.175	204.72		
	KSLNSSRAK	A3	212	HLA-A*30:01	0.3	16.01	1.3136	1.2987
				HLA-A*03:01	0.35	71.31		
				HLA-A*11:01	0.855	72.67

**Table 2:** HTL epitopes predicted using NetMHC II pan 3.2 server. Position indicates the starting residue of the proteins being studied. Epitopes with score ≤ 2.0 are considered as strong binders and epitopes with antigenicity score > 0.4 are considered to be antigenic which have the possibility of generating an effective immune response.

HTL EPITOPES					
Protein	Epitopes	Position	Allele	Score	Antigenic score
FliC	MRVNTNVSALIANNQ	1	DRB1*13:02	0.50	0.4654
			DRB3*02:02	0.90	
			DQA1*01:02-DQB1*06:02	0.60	
	NNNEIKIQLVNTASI	161	DRB1*04:01	2.00	0.9979
			DRB1*08:02	1.90	
			DRB1*13:02	1.50	
			DRB4*01:01	0.20	
	NNEIKIQLVNTASIM	162	DRB1*04:01	1.10	0.7263
			DRB1*04:05	1.90	
			DRB1*08:02	0.90	
			DRB1*12:01	1.80	
			DRB1*13:02	0.50	
			DRB4*01:01	0.10	
CotE	NNQILRTILYYPLTT	133	DRB1*12:01	1.00	0.4123
			DPA1*02:01-DPB1*01:01	1.90	
			DPA1*01:03-DPB1*02:01	1.80	
			DPA1*01:03-DPB1*04:01	1.60	
			DPA1*03:01-DPB1*04:02	1.80	
	AQLLDAVIFAFAEID	396	DQA1*05:01-DQB1*02:01	1.00	0.4179
			DQA1*03:01-DQB1*03:02	0.40	
			DQA1*04:01-DQB1*04:02	0.40	
	KDGDFAMSYDDALSI	645	DRB3*01:01	1.30	0.5761
			DQA1*03:01-DQB1*03:02	1.70	
			DQA1*01:01-DQB1*05:01	0.40	
			DQA1*05:01-DQB1*02:01	0.10	
SlpA	RYETSLAIADEIGLD	513	DQA1*05:01-DQB1*02:01	0.25	0.7434
			DQA1*03:01-DQB1*03:02	0.07	
			DQA1*04:01-DQB1*04:02	0.07	
	ETSLAIADEIGLDND	515	DQA1*05:01-DQB1*02:01	1.30	0.6268
			DQA1*03:01-DQB1*03:02	1.00	
			DQA1*04:01-DQB1*04:02	1.40	
	IAGRFKESPAPIILA	660	DRB1*07:01	0.60	0.4282
			DRB1*09:01	0.50	
			DRB3*02:02	1.80	
			DPA1*02:01-DPB1*14:01	1.40

IFN-γ epitopes also play an important role in inducing innate as well as adaptive immunity as it triggers the release of IFN-γ, a signature cytokine released by the neutrophils during *Clostridium difficile* infection^45,46^. Therefore, IFN-γ epitopes were predicted from all the three proteins (Supplementary Table_IFN γ epitope), and the best scoring epitopes from each protein were included in the final vaccine construct.

### Multi-epitope vaccine construction, structure modelling and validation

**Figure 3:**
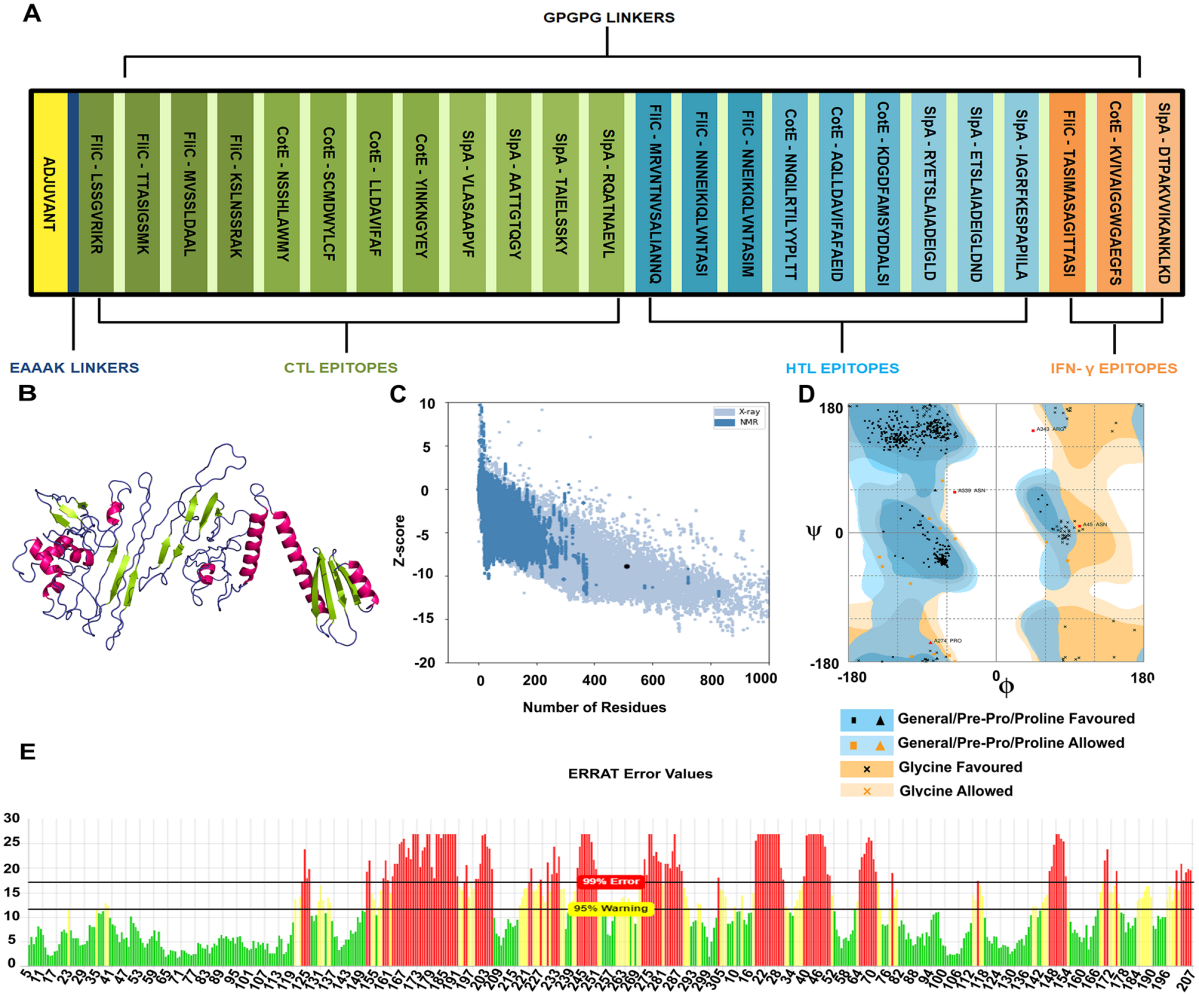
(**A**) Schematic representation of linear vaccine construct showing CTL epitopes in green colour, HTL epitopes in blue colour and IFN-γ in orange colour. Pale green colour represents GPGPG linkers, which have been used to link epitopes together. CTB adjuvant is represented in yellow colour, joined to the linear vaccine using deep blue EAAAK linker. (**B**) Tertiary structure of the vaccine. Limon, blue and hot pink colour represents the sheets, loops and helices, respectively. (**C**) Protein structure validation by ProSA with a Z-score of − 8.92. (**D**) Ramachandran plot analyses showing 96.5%, 2.7% and 0.8% in favoured, allowed and outlier regions, respectively as predicted by RAMPAGE (**E**) Protein structure validation by ERRAT score of 53.3865, where X axis denotes residues and Y axis denotes error values.

The chimeric vaccine was constructed according to few criteria, namely: (1) it should be antigenic and non-allergenic, (2) it should have overlapping HTL and CTL epitopes (Supplementary Table S7), (3) it must be immunogenic and must be having high affinity to HLA alleles. Following these requirements, a linear vaccine construct was designed using 12 CTL epitopes (4 CTL epitopes from each protein), 9 HTL epitopes (3 HTL epitopes from each protein) and 3 IFN-γ epitopes (1 IFN-γ epitope from each protein). These predicted epitopes were joined with each other using GPGPG linkers. These linkers help in the prevention of potential junctional epitopes and also facilitate immune processing^47,48^. An adjuvant, Cholera Toxin B (CTB) was attached to the N-terminal of the vaccine using the EAAAK linker (Fig. 3A), as it has been used for the construction of many fusion proteins and acts as a rigid spacer between the protein domains^49–51^. The addition of CTB adjuvants in vaccines improves the formation of a protective immunity as well as prevents chances of autoimmune reactions^52^. Considering the preliminary criteria for vaccine construction, 6 constructs were made depending upon the position of the epitopes from the three *Clostridium difficile* proteins (Supplementary Material SM1). The 3D structures of all the constructs were checked for their efficiency based on Z-scores, ERRAT and Ramachandran plot analysis (Supplementary Table S8) and the one with the best scores was chosen as our final vaccine construct. The final vaccine construct has a molecular weight of 51,649.46 Da, consisting of 512 amino acids. A tertiary structure of the final linear vaccine was generated using trRosetta web-server (Fig. 3B)^53^. The Ramachandran plot of the model structure was generated using RAMPAGE^54^, which showed that 96.5% residues lie in favoured region, 2.7% lie in allowed and 0.8% residues lie in outlier region (Fig. 3D). This analysis verifies the overall quality of the vaccine model. Also, the generated Z-score of the 3D model was found to be − 8.92, as predicted by ProSA webserver, which indicates that the protein falls in the plot which consists of Z-scores of the already determined structures solved by NMR and X-ray crystallographic experiment (Fig. 3C)^55^. For further validation, ERRAT analysis was performed which resulted in ERRAT score of 53.3865, representing the percentage of residues falling under 95% rejection limit (Fig. 3E)^56^**.** An ERRAT score more than 50 denotes a good quality model; hence a score of 53.3865 further confirms the structural validation of the protein^57^.

### Antigenicity, allergenicity, immunogenicity and physicochemical properties of the vaccine construct

The capacity to induce humoral and cellular immune responses is defined by immunogenicity while the ability to recognize a specific antigen, which is further accompanied by the generation of an immune response is termed as antigenicity. Hence, in order to recognize an antigen and elicit an immune response with the development of both humoral and cell-mediated immunity, the vaccine should be antigenic as well as immunogenic. The antigenicity of the vaccine was confirmed using VaxiJen v2.0 with a score of 0.9103, where scores of sequences > 0.4 (the predefined cut off or threshold value) are considered to be antigenic^58^. The immunogenicity of the vaccine was 2.30148, as predicted by the IEDB class I immunogenicity tool (according to IEDB a higher score indicates greater chances in generating an immune response). To assure that no allergenic reactions are triggered by the vaccine, allergenicity of the vaccine was checked. The designed vaccine was predicted to be a non-allergen by AllerTOP webserver. The physicochemical properties of the vaccine were predicted by ExPASy ProtParam tool in order to determine its safety and efficacy (Supplementary Material SM2). The predicted aliphatic index of the vaccine is 76.15, which denotes the vaccine to be stable at high temperatures^59^. Generally, for a protein, higher the aliphatic index, higher is the protein’s thermostability^60^. The vaccine has an instability index of 22.16, which means it is quite stable since; in general, an instability index below 40 indicates protein stability^60^. The grand average hydropathicity (GRAVY) score is − 0.162, which indicates the hydrophilic nature of the vaccine: a lower GRAVY score is the indication of better solubility of the protein in an aqueous environment^61^. An estimated half-life of the vaccine is 30 h in mammalian reticulocytes, > 20 h in yeast and > 10 h in *Escherichia coli* by ExPASy ProtParam tool^60^. There are no transmembrane helices or signal peptides detected in the vaccine structure, which signifies no expression difficulties of the protein and prevention of protein localization, respectively (Supplementary Figs. S1 and S2).

### B cell epitope, IL-2 inducing, pro-inflammatory cytokine inducing and IFN-γ inducing HTL epitope predictions

B cells are liable for the humoral immune response^62^. Helper T cells help in the activation and differentiation of B cells to specific antibody-secreting plasma B cells. Therefore, the presence of B cell epitopes is important in vaccine designing. In this study, the ElliPro server was used to predict the presence of linear/continuous and conformational/discontinuous B cell epitopes in the vaccine construct, under default parameters (Tables 3, 4). The predicted B cell epitopes in the vaccine construct were also visualized using PyMOL (Supplementary Fig. S3).

**Table 3:** Linear/continuous B cell epitopes as predicted by ElliPro server.

Linear epitopes	Position	Score
MTPQNITDLCAEYHNTQIHTLNDKIFSYTESLAGKREMAIITFKNGATFQVEVPGSQHIDSQKKAIERMKDTLR	1–74	0.831
ASAAPVFGPGPGAATTGT	224–241	0.801
FSGPGPGDTPAKVVIKANKLKD	491–512	0.732
ANNQGPGPGNNNE	289–330	0.72
SIMGPGPGNNQI	330–341	0.704
EIDGPGPGKDGDFAMSYDDALSIGPGPGRYE	370–400	0.699
GLDNDGPGPGI	428–438	0.674
ASIGPGPGKVIV	470–481	0.669
TTGPGPGA	351–358	0.649
LAIADEIGLDGPGPGETSLA	403–422	0.635
AKVEKLCVWNNKTPH	81–95	0.626
IGPGPGNN	312–319	0.602
SRAKGPGPGNS	157–167	0.57
GPGPGLL	189–195	0.567
LAGPGPGT	451–458	0.558

**Table 4:** Discontinuous/conformational B cell epitopes as predicted by ElliPro server.

Discontinuous epitopes	Score
KLK (509–511)	0.848
MTPQNITDLCAEYHNTQIHTLNDKIFSYTESLAGKREMAIITFKNGATFQVEVPGSQHIDSQKKAIERMK (1–70) L (73) YL (77–78) AK (81–82) EK (84–85)	0.822
INKNGY (209–214) ASAAPNFGPGPGAATTGTQ (224–242)	0.708
KAN (506–508)	0.693
ANNQGPGPGNNNESIMGPGPGNN (289–339) I (341) AEIDGPGPGKDGDFAMS (369–385) DDALSIGPGPGRYE (387–400) LAADEIGLDGPGPGETSLA (403–422) D (425) GLDNDGPGPGI (428–438) R (441) KESP (443–446) ILAGPGPGT (450–458) ASIGPGPGKVI (470–480) EGFSGPGPGDTPAKV (489–504)	0.674
LC (86–87) NK (91–92)	0.659
IGPGPGNN (312–319) TTGPGPGA (351–358)	0.625
K (152) AKGPGPGNS (159–167) GPGPGLL (189–195)	0.578
TPH (93–94)	0.574

The selected HTL epitopes were subjected to prediction for IL-2 inducing epitopes using IL2Pred server in order to ensure their effectiveness in inducing interleukin-2 [https://webs.iiitd.edu.in/raghava/il2pred/stat.php]. The results indicated that 5 of our selected HTL epitopes are IL-2 inducing epitopes (Supplementary Table S9), thereby assuring a strong the immune response.

Additionally, the CD4+ epitopes included in our vaccine were checked for pro-inflammatory cytokine inducing epitopes by the server ProInflam^63^. The server indicates that the epitopes which are pro-inflammatory cytokine inducers can induce cytokines like TNF, IL-18, IL-12, IL-23^63^. 5 of the 9 HTL epitopes were predicted as inducers of pro-inflammatory cytokines, which has the capacity to induce strong immune response in the body (Supplementary Table S10). The HTL epitopes were also checked for their ability to induce IFN-γ by subjecting them to the Predict Algorithm of IFNepitope server^46^. Results show that 2 epitopes with positive scores are effective IFN-γ inducers (Supplementary Table S11).

### Population coverage

Development of a successful vaccine demands the assessment of HLA allele distribution around the world population^64^. Based on the difference between regions and ethnicities, the distribution and expression of HLA alleles may vary across the world^65^. Hence, it is necessary to evaluate if the designed vaccine will be effective against the world population. The selected epitopes in the study have world population coverage of 98.55% (Table 5). In addition, the epitopes showed 99.54%, 99.1%, 94.15%, 95.03%, 95.68% coverage in Europe, United States, China, South Asia and Oceania, respectively (Table 5) (Supplementary Fig. S4). The results are suggestive of the fact that the designed vaccine candidate has the effectiveness to tackle CDI infection globally.

**Table 5:** Population coverage of the selected epitopes of the vaccine construct, as predicted by IEDB server (pc: population coverage).

Population/area	Coverage (%)	Average hit	pc90
World	98.55	4.03	2.06
Europe	99.54	4.34	2.32
United States	99.1	4.36	2.19
China	94.15	3.26	1.41
South Asia	95.03	3.13	1.42
Oceania	95.68	3.18	1.94

### Molecular docking studies

In order to study the interaction between the vaccine construct with the Toll-Like Receptors and MHC molecules, molecular docking studies using HADDOCK server was performed^66^. TLRs play a major role in adaptive immunity, forming the first line of defence against infections. On interaction with a wide range of microbes and viruses, these TLRs detect the pathogen-associated molecular patterns (PAMPs) on the microbes, which trigger the activation of innate immunity along with orchestration of humoral immune response^67^. TLR4 is known to recognize bacterial surface and coat proteins while TLR5 recognises flagellin proteins, which leads to an active immune response in body^68,69^. Hence, it is important for the designed vaccine to interact with target immune receptors like TLRs. The interaction of MHC I and MHC II molecules with the vaccine helps in activating the CTLs and HTLs, which are required for immune response generation^70^. Thus, the docking analyses of vaccine-MHC molecules is also important for this study.

#### Docking of the epitope with HLA alleles

**Figure 4:**
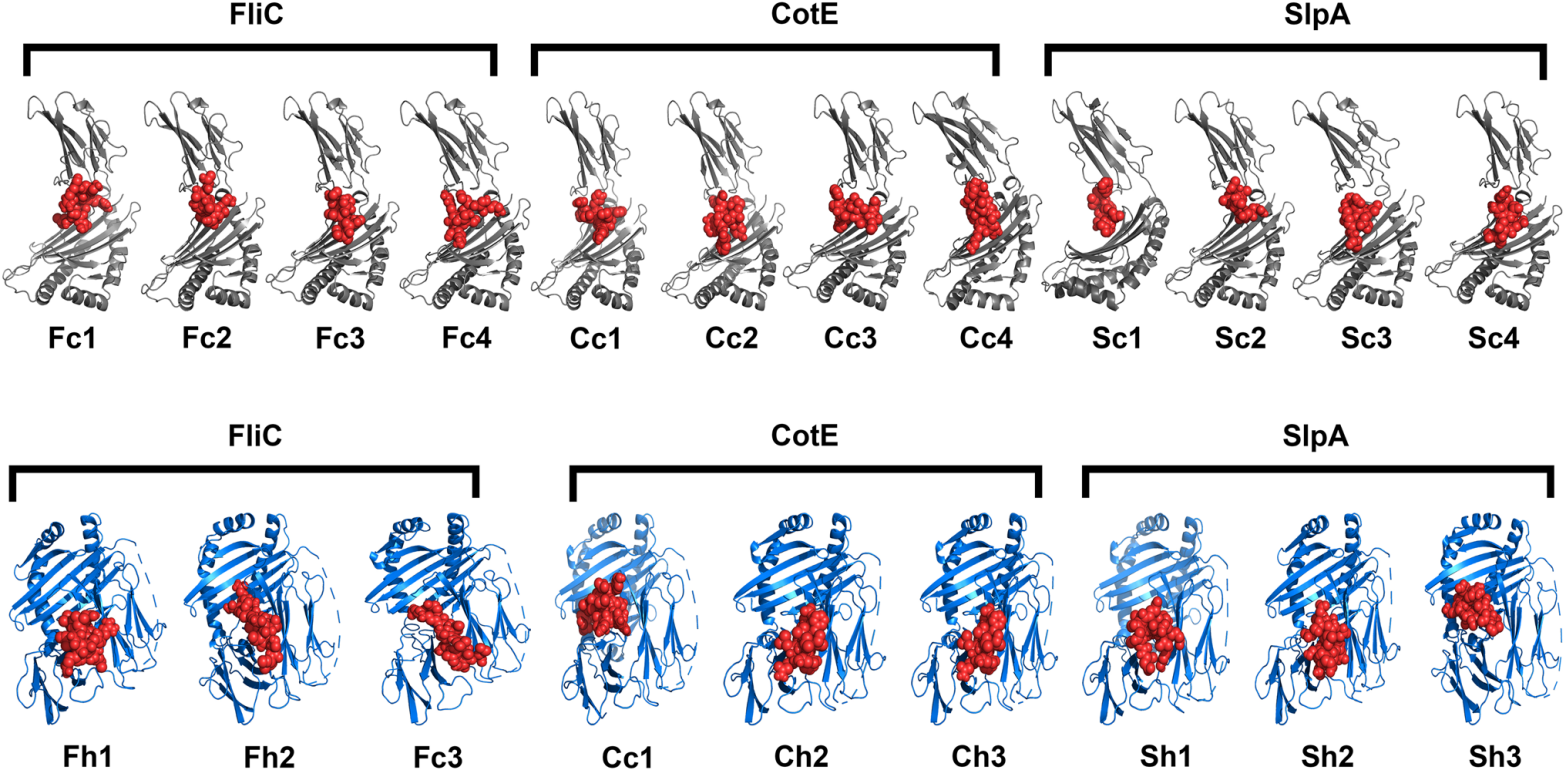
Docking of individual epitopes with MHC class I and class II receptors. ‘c’ represents the CTL epitopes docked with MHC class I receptor (grey colour) and ‘h’ represents the HTL epitopes docked with MHC class II receptor (blue colour). All the epitopes are depicted in red colour spheres. ‘F’ denotes epitopes derived from FliC protein, ‘C’ denotes epitopes from CotE protein and ‘S’ denotes the epitopes from SlpA protein. The arrangement of the epitopes are based on their position in the vaccine construct.

The predicted CTL and HTL epitopes were docked with the MHC class I and MHC class II receptor molecules, respectively. This was done for analysing the binding patters of each epitope with their respective receptors (Fig. 4). A detailed overview showing all the binding interactions between the CTL epitopes with MHC I and HTL epitopes with MHC II have been shown in Supplementary Figs. S5 and S6.

#### Docking of the vaccine with TLR4

**Figure 5:**
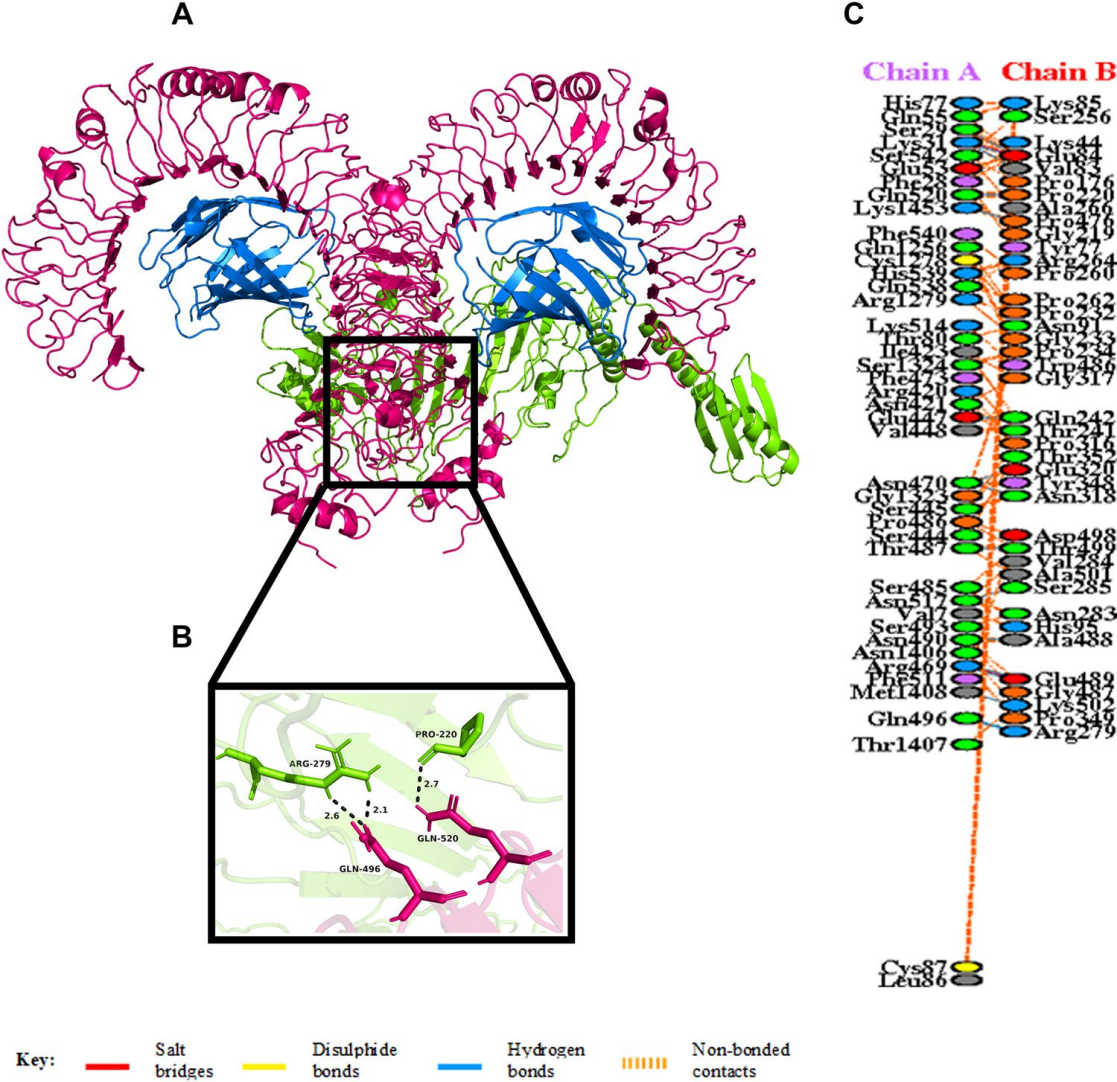
(**A**) Figure acquired after molecular docking which shows docked TLR4/MD2-vaccine complex. The vaccine construct is shown in green while the TLR4 dimer is shown in hotpink, and the blue colour represents the MD2 co-receptor. (**B**) Residue interaction between the docked tetramer TLR4 / MD2 (chain A) and the vaccine (chain B). (**C**) Few hydrogen bonds shown within vaccine-TLR4 complex are focused.

The HADDOCK server necessitates the ambiguous interaction restrains (AIRs), which highlights the active and passive residues of the receptor and the ligand required for docking. The interaction in between molecules and available solvent involves the active residues, while the surface neighbour active residues denote the passive residues. Here, the vaccine candidate is docked with the TLR4 heterodimer to study the interaction between vaccine and TLR4. Among the many predicted clusters, the best reliable cluster with the lowest HADDOCK score was subjected to refinement by the HADDOCK Refinement Server. The statistics of the refined structure is detailed in Table 6 and Supplementary Fig. S7. During refinement, 20 structures were clustered into one cluster, resulting in 100% water refined model. After refinement, the model showed a HADDOCK score of − 204.2 ± 2.0 a.u., which suggests good binding affinity between the receptor (TLR4) and ligand (vaccine) as a negative HADDOCK score suggests better docking. Less water-exposed protein surface and close binding proximity is indicated by the Buried Surface Area (BSA) of 5042.9 ± 160.6 Å^2^^71^. A low RMSD score of 0.3 ± 0.2 Å of the docked structure indicates a good quality model. A detailed overview of the docked complex along with interacting amino acid residues are shown in Supplementary Fig. S8. The docked complex along with the interacting residues is shown in Fig. 5 and detailed interaction is given in Supplementary Material SM3.

**Table 6:** Statistics of the best refined docked TLR4/MD2 and vaccine complex. Strong protein interaction expressed in arbitrary units (a.u.) is represented by smallest HADDOCK score.

Vaccine-TLR4	
HADDOCK score (a.u.)	− 204.2 ± 2.0
Cluster size	20
RMSD from the overall lowest-energy structure (Å)	0.3 ± 0.2
Van der Waals energy (kcal mol^−1^)	− 142.4 ± 4.9
Electrostatic energy (kcal mol^−1^)	− 470.4 ± 17.4
Desolvation energy (kcal mol^−1^)	32.2 ± 5.4
Restraints violation energy (kcal mol^−1^)	1.3 ± 0.62
Buried Surface Area (Å^2^)	5042.9 ± 160.6

#### Docking of the vaccine with TLR5

**Figure 6:**
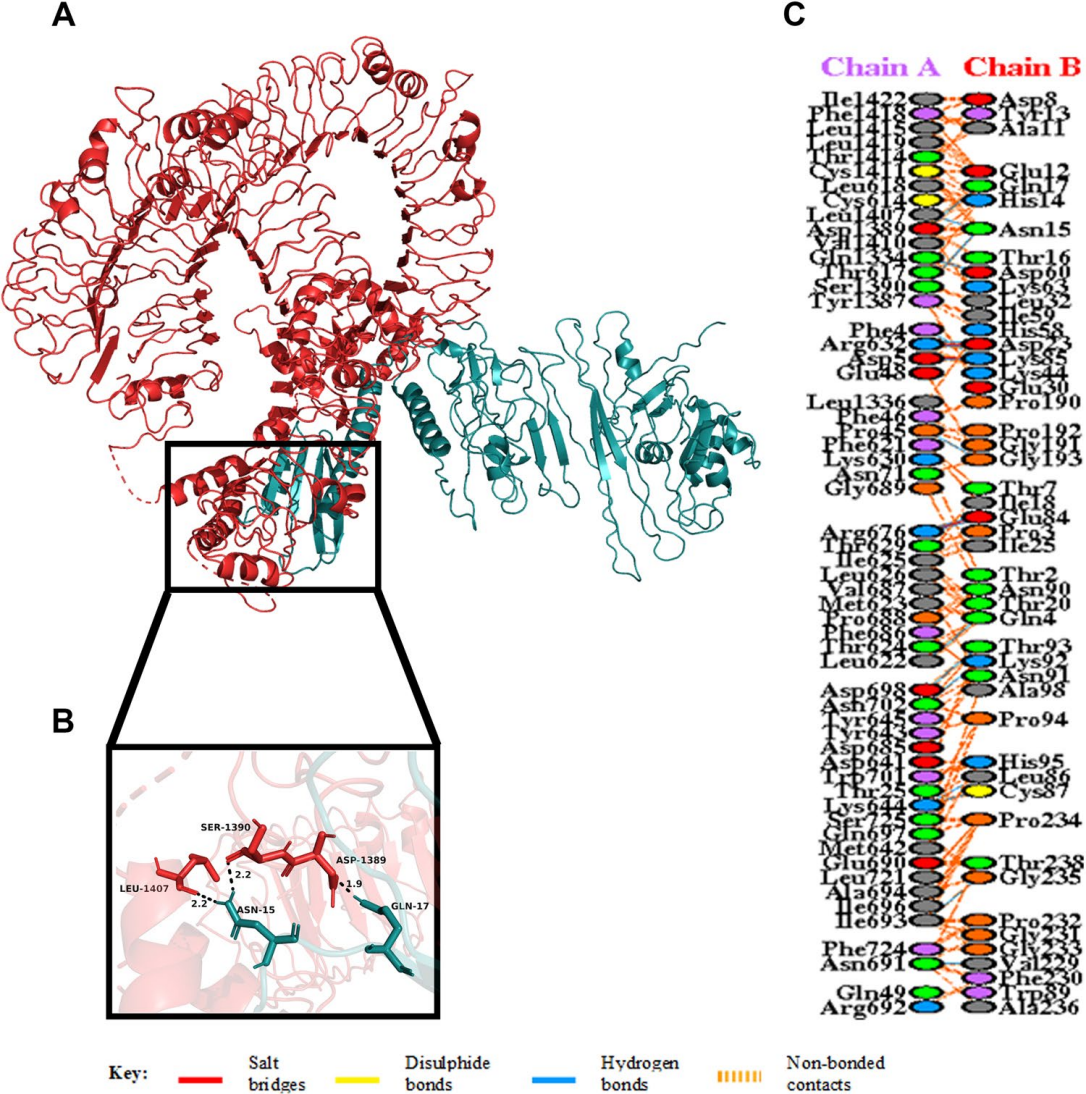
(**A**) Figure acquired after molecular docking which shows docked TLR5-vaccine complex. The vaccine construct is shown in teal while the TLR5 dimer is shown in red. (**B**) Residue interaction between the docked tetramer TLR5 (chain A) and the vaccine (chain B). (**C**) Few hydrogen bonds shown within vaccine-TLR5 complex are focused.

The HADDOCK server necessitates the ambiguous interaction restrains (AIRs), which highlights the active and passive residues of the receptor and the ligand. The interaction in between molecules and available solvent involves the active residues, while the surface neighbour active residues denote the passive residues. Here, the vaccine candidate is docked with TLR5 to study the interaction between vaccine and TLR5 molecule. Among the many predicted clusters, the best reliable cluster with the lowest HADDOCK score was subjected to refinement by the HADDOCK Refinement Server. The statistics of the refined structure is detailed in Table 7 and Supplementary Fig. S9. During refinement, 20 structures were clustered into one cluster, resulting in 100% water refined model. After refinement, the model showed a HADDOCK score of − 259.8 ± 3.1 a.u., which suggests good binding affinity between the receptor (TLR5) and ligand (vaccine) as a negative HADDOCK score suggests better docking. Less water-exposed protein surface and close binding proximity is indicated by the Buried Surface Area (BSA) of 5358.9 ± 45.0 Å^2^^71^. A low RMSD score of 0.3 ± 0.2 Å of the docked structure indicates a good quality model. A detailed overview of the docked complex along with interacting amino acid residues are shown in Supplementary Fig. S10. The docked complex along with the interacting residues is shown in Fig. 6 and detailed interaction is given in Supplementary Material SM4.

**Table 7:** Statistics of the best refined docked TLR5 and vaccine complex. Strong protein interaction expressed in arbitrary units (a.u.) is represented by smallest HADDOCK score.

Vaccine-TLR5	
HADDOCK score (a.u.)	− 259.8 ± 3.1
Cluster size	20
RMSD from the overall lowest-energy structure (Å)	0.3 ± 0.2
Van der Waals energy (kcal mol^−1^)	− 182.2 ± 4.2
Electrostatic energy (kcal mol^−1^)	− 514.1 ± 25.7
Desolvation energy (kcal mol^−1^)	25.1 ± 6.0
Restraints violation energy (kcal mol^−1^)	1.1 ± 0.39
Buried surface area (Å^2^)	5358.9 ± 45.0

#### Docking of the vaccine with MHC I and MHC II receptors

**Figure 7:**
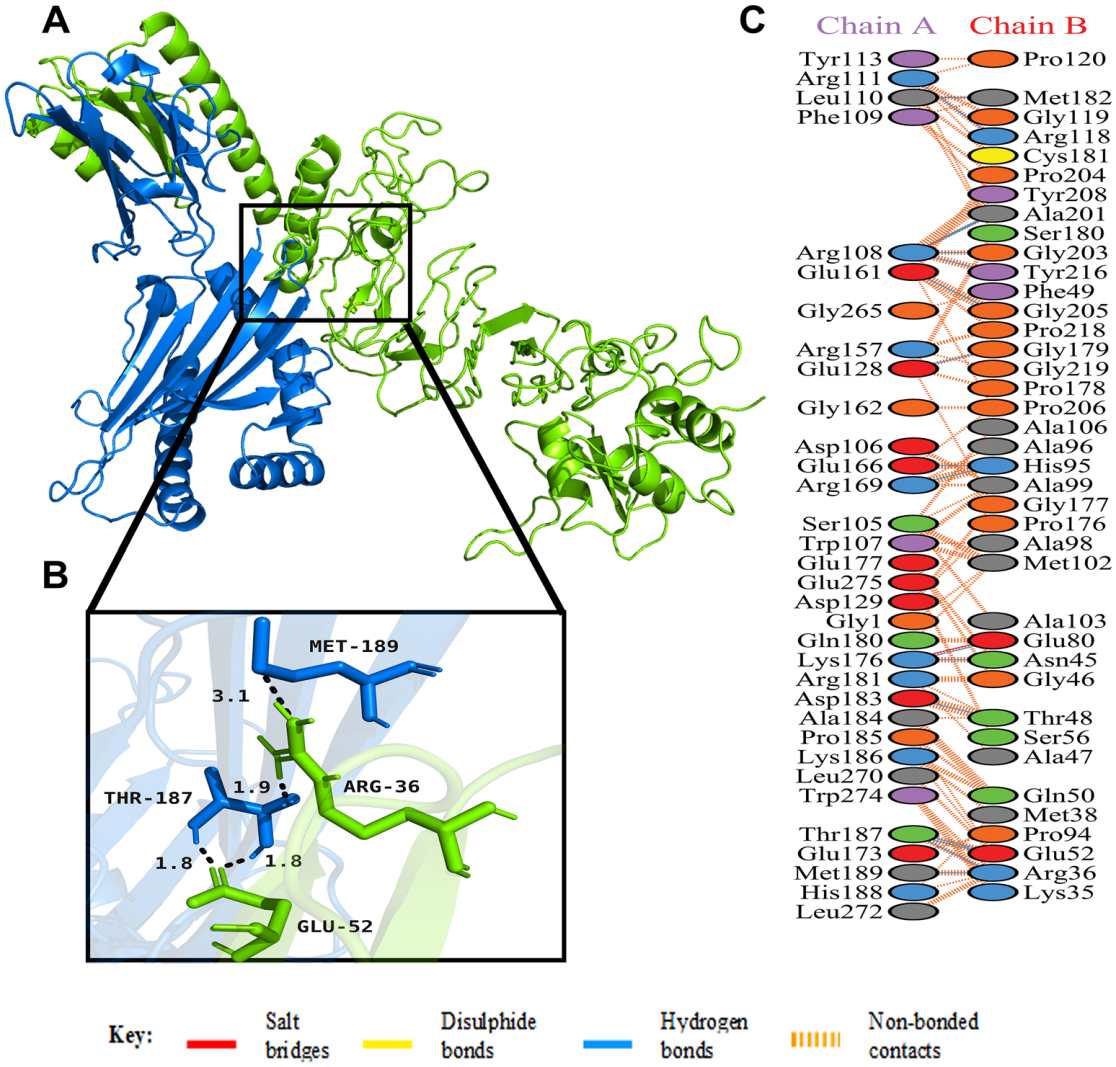
(**A**) Figure acquired after molecular docking which shows docked MHC class I receptor-vaccine complex. The vaccine construct is shown in green while the MHC class I receptor is shown in blue. (**B**) Residue interaction between the docked MHC class I receptor (chain A) and the vaccine (chain B). (**C**) Few hydrogen bonds shown within vaccine-MHC class I receptor complex are focused.

**Figure 8:**
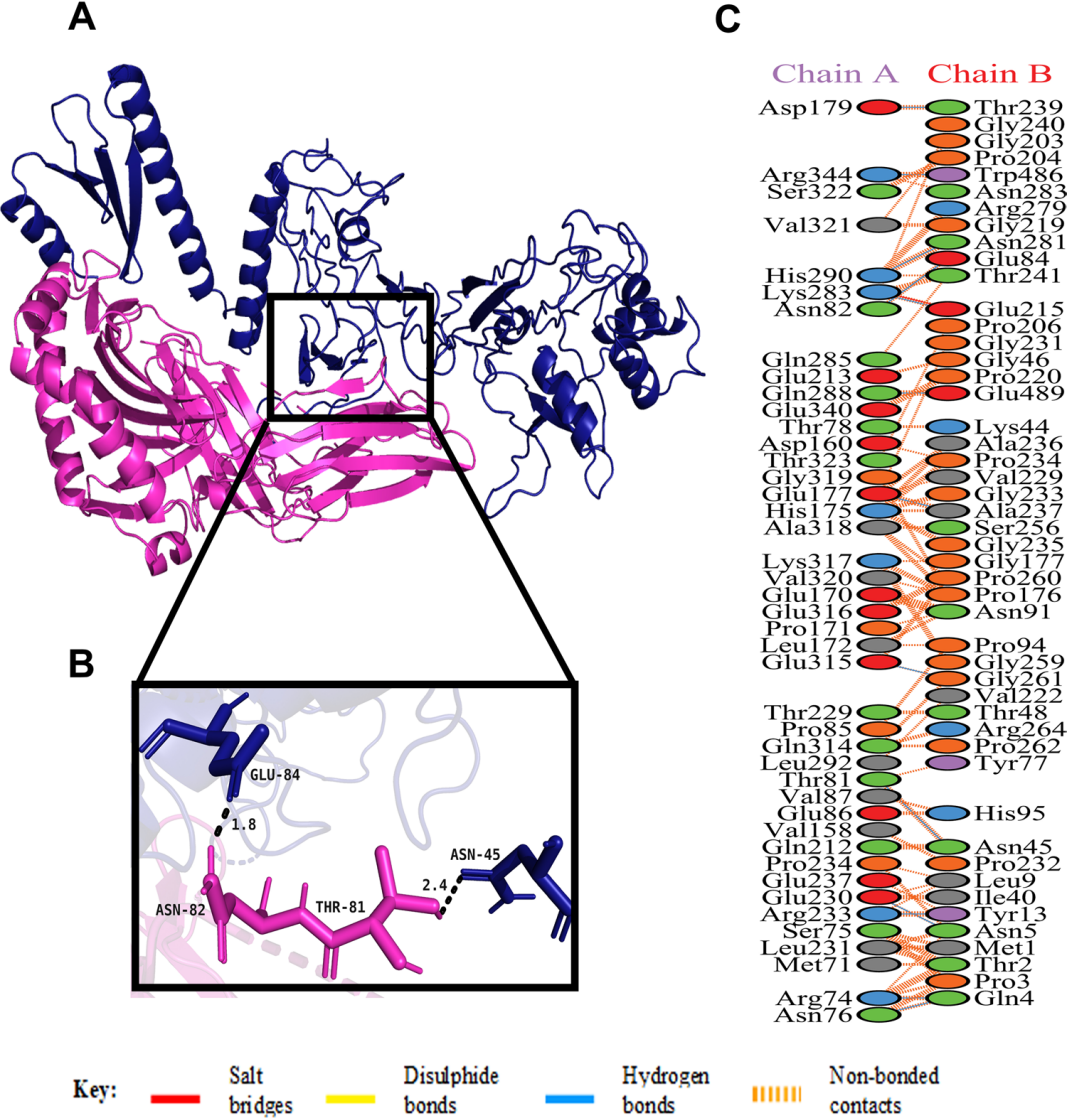
(**A**) Figure acquired after molecular docking which shows docked MHC class II receptor-vaccine complex. The vaccine construct is shown in deep blue while the MHC class II receptor is shown in purple. (**B**) Residue interaction between the docked MHC class II receptor (chain A) and the vaccine (chain B). (**C**) Few hydrogen bonds shown within vaccine-MHC class II receptor complex are focused.

The HADDOCK server requires the ambiguous interaction restrains (AIRs), which highlights the active and passive residues of the receptor and the ligand. The interaction in between molecules and available solvent involves the active residues, while the surface neighbour active residues denote the passive residues. Here, the vaccine candidate is docked with the MHC I and MHC II molecules separately to study the interaction between vaccine and MHC molecules. Among the many predicted clusters, the best reliable clusters for both the complexes having lowest HADDOCK scores were subjected to refinement by the HADDOCK Refinement Server. The statistics of the refined structure is detailed in Table 8 and Supplementary Figs. S11–S12. During refinement, 20 structures for each complex were clustered into one cluster, resulting in 100% water refined model. After refinement, the vaccine-MHC I and vaccine-MHC II docked models showed the HADDOCK scores of − 208.6 ± 8.4 a.u. and − 252.4 ± 4.1 a.u., respectively, which suggests good binding affinity between the receptors (MHC I and MHC II) and ligand (vaccine) as negative HADDOCK score suggests better docking. Less water-exposed protein surface and close binding proximity is indicated by the Buried Surface Area (BSA) of 3956.3 ± 75.1 Å^2^ for vaccine-MHC I complex and 5169.4 ± 45.9 Å^2^ for vaccine-MHC II complex^71^. Low RMSD score of 0.3 ± 0.2 Å (vaccine-MHC I complex) and 0.3 ± 0.2 Å (vaccine-MHC II complex) of the docked structures indicates them to be good quality models. A detailed overview of the docked complexes along with interacting amino acid residues are shown in Supplementary Figs. S13 and S14. The docked complexes along with their interacting residues are shown in Fig. 7 (vaccine-MHC I complex) and Fig. 8 (vaccine-MHC II complex). The detailed interactions of vaccine-MHC I and vaccine-MHC II complexes are given in Supplementary Materials SM5 and SM6, respectively.

**Table 8:** Statistics of the best refined docked vaccine-MHC I and vaccine-MHC II complexes. Strong protein interaction expressed in arbitrary units (a.u.) is represented by smallest HADDOCK score.

Parameters	Vaccine-MHC I	Vaccine-MHC II
HADDOCK score (a.u.)	− 208.6 ± 8.4	− 252.4 ± 4.1
Cluster size	20	20
RMSD from the overall lowest-energy structure (Å)	0.3 ± 0.2	0.3 ± 0.2
Van der Waals energy (kcal mol^−1^)	− 140.4 ± 5.8	− 189.9 ± 3.0
Electrostatic energy (kcal mol^−1^)	− 384.3 ± 27.6	− 342.3 ± 33.1
Desolvation energy (kcal mol^−1^)	8.6 ± 5.2	5.9 ± 9.2
Restraints violation energy (kcal mol^−1^)	0.0 ± 0.00	0.1 ± 0.06
Buried Surface Area (Å^2^)	3956.3 ± 75.1	5169.4 ± 45.9

### Binding affinity analysis

The binding affinity of a complex or the Gibbs free energy (∆G) in terms of thermodynamics, is an important quantity to determine if an interaction will actually occur or not in the cell at specific conditions^72,73^. Hence, the binding affinity of the docked structures were analysed using the PRODIGY web server^72^, which provided the ∆G values of the vaccine-TLR4, vaccine-TLR5, vaccine-MHC I and vaccine-MHC II complexes as − 17.2, − 18.2, − 16.7 and − 17.2, respectively. It can be concluded that all the interactions are energetically feasible, which is evident from the negative values of Gibbs free energy. The corresponding ∆G values along with the dissociation constant (K_d_) values of the docked complexes are provided in the Table 9.

**Table 9:** The PRODIGY server predicted the binding affinities of the docked complexes with vaccines and TLR4, TLR5, MHC I receptor, and MHC II receptor.

Complexes	Gibbs free energy (kcal mol^−1^)	K_d_ (M)
Vaccine-TLR4	− 17.2	2.4E−13
Vaccine-TLR5	− 18.2	4.6E−14
Vaccine-MHC class I receptor	− 16.7	6.0E−13
Vaccine-MHC class II receptor	− 17.2	2.4E−13

### Energy minimization and molecular dynamics simulation (MDS) of vaccine-TLR complexes

**Figure 9:**
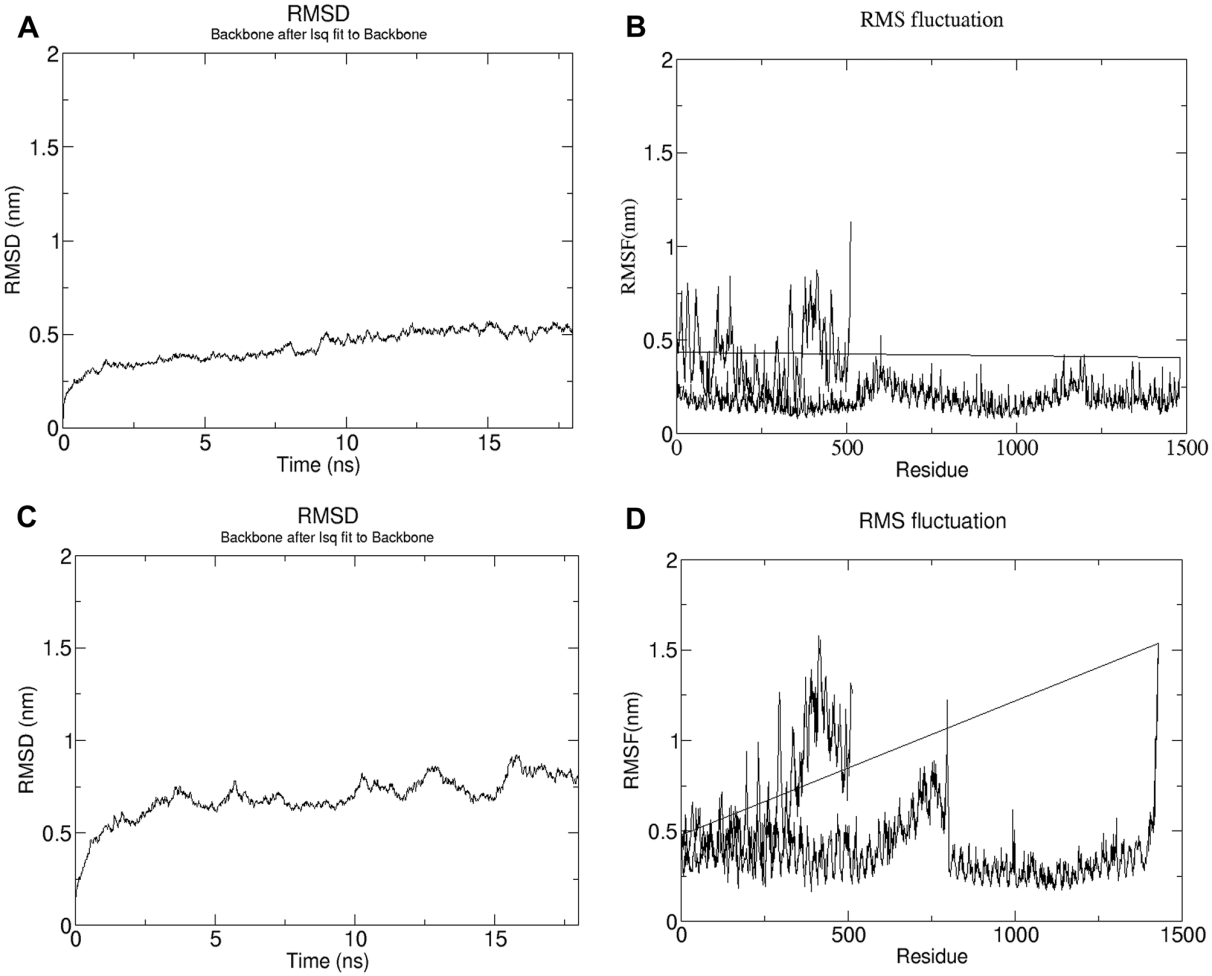
(**A**) RMSD plot of the vaccine-TLR4 complex backbone. (**B**) RMSF plot of vaccine-TLR4 complex with regions of flexibility. (**C**) RMSD plot of the vaccine-TLR5 complex backbone. (**D**) RMSF plot of vaccine-TLR5 complex with regions of flexibility.

The evaluated potential energy of the vaccine-TLR4 complex was − 9.83e + 06 kJ/mol and the plot for the same indicates a steady convergence of potential energy during this step (Supplementary Fig. S15). The consistent low energy of − 9.83e + 06 kJ/mol of the energy minimized structure supported it to a reasonable starting structure for carrying out further steps of MDS. An equilibration phase for 100 ps was done for studying the effect of temperature, pressure and other thermodynamic properties. The NVT ensemble was used for conducting the first equilibration phase to check if the system was stable at the desired temperature of 300 K. Results for this step indicated that the temperature of the system quickly reached 300 K which was maintained during the equilibration phase with very minimal fluctuations indicating the system to be stable at 300 K (Supplementary Fig. S15). In addition, the NPT ensemble evaluated the stability of the system with respect to the desired pressure of 1 bar. In this case the results indicated that the pressure of 1 bar was also maintained during equilibration and negligible fluctuations observed in the plot confirmed its stability for other trajectory analyses. The average density of the system was found to be 1017.3 kg/m^3^ with a total drift of 1.6 kg/m^3^. Similar results were observed for vaccine-TLR5 complex at same conditions of temperature and pressure (Supplementary Fig. S15). The computed average potential energy for this system was − 1.12e + 07 kJ/mol, with a density of 1009 kg/m^3^ having a total drift of 1.5 kg/m^3^. After a simulation period of 18 ns, a trajectory analysis was also performed to check the flexibility and stability of the vaccine-TLR complexes. The plots for the radius of gyration demonstrated the compactness of the protein along its axes (Supplementary Fig. S15). Very mild fluctuations in the plot of the RMSD backbone indicate that the vaccine-TLR complexes formed strong interactions among themselves hence, attaining greater stability of the complex over time. The RMSF plots for the vaccine-TLR complexes identified regions of high flexibility as indicated by the high peaks observed in the RMSF plot (Fig. 9). Though there were few fluctuating residues, the plots are also suggestive of the fact that those fluctuations were minimal and the vaccine-TLR complexes were mostly stable over time (Fig. 9).

### Codon optimization and in silico cloning of vaccine

**Figure 10:**
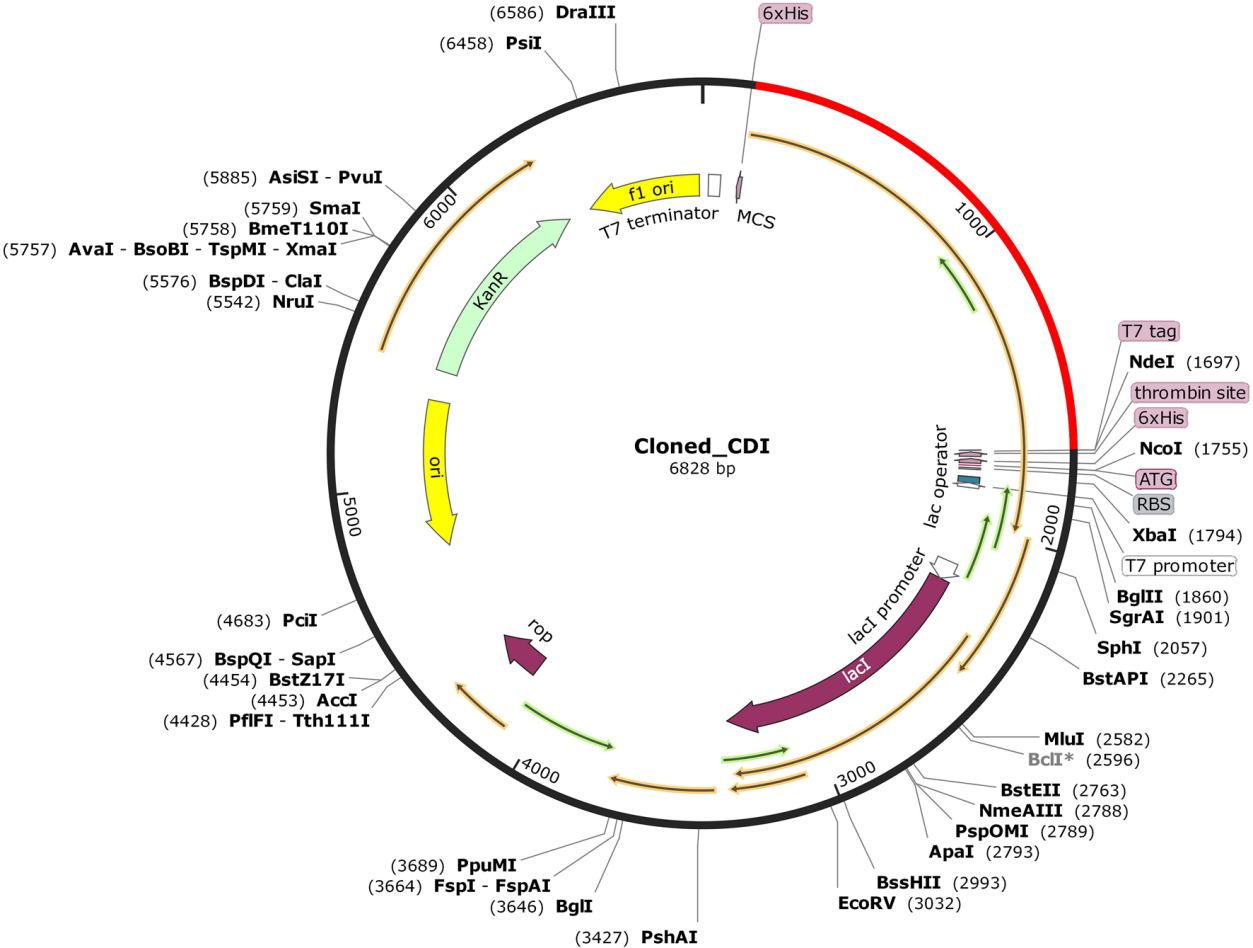
In silico restriction cloning. The black colour part represents the pET28a (+) expression vector in which the codon optimized multi-epitope vaccine is inserted (red colour part).

The Java Codon Adaptation Tool (JCat) has been used to optimize codon usage of the vaccine construct in *E. coli* (strain K12) for optimal protein expression. The optimized nucleotide sequence has Codon Adaptation Index (CAI) of 1.0 and the average GC content of the adapted sequence is 55.46875, showing the vaccine candidate's potential for good expression in *E. coli* host^74^. The ideal GC content percentage range is between 30 and 70^74^. Finally, the recombinant plasmid sequence was designed using SnapGene tool by inserting the adapted codon sequences into the pET28a (+) vector (Fig. 10).

### Immune simulation

**Figure 11:**
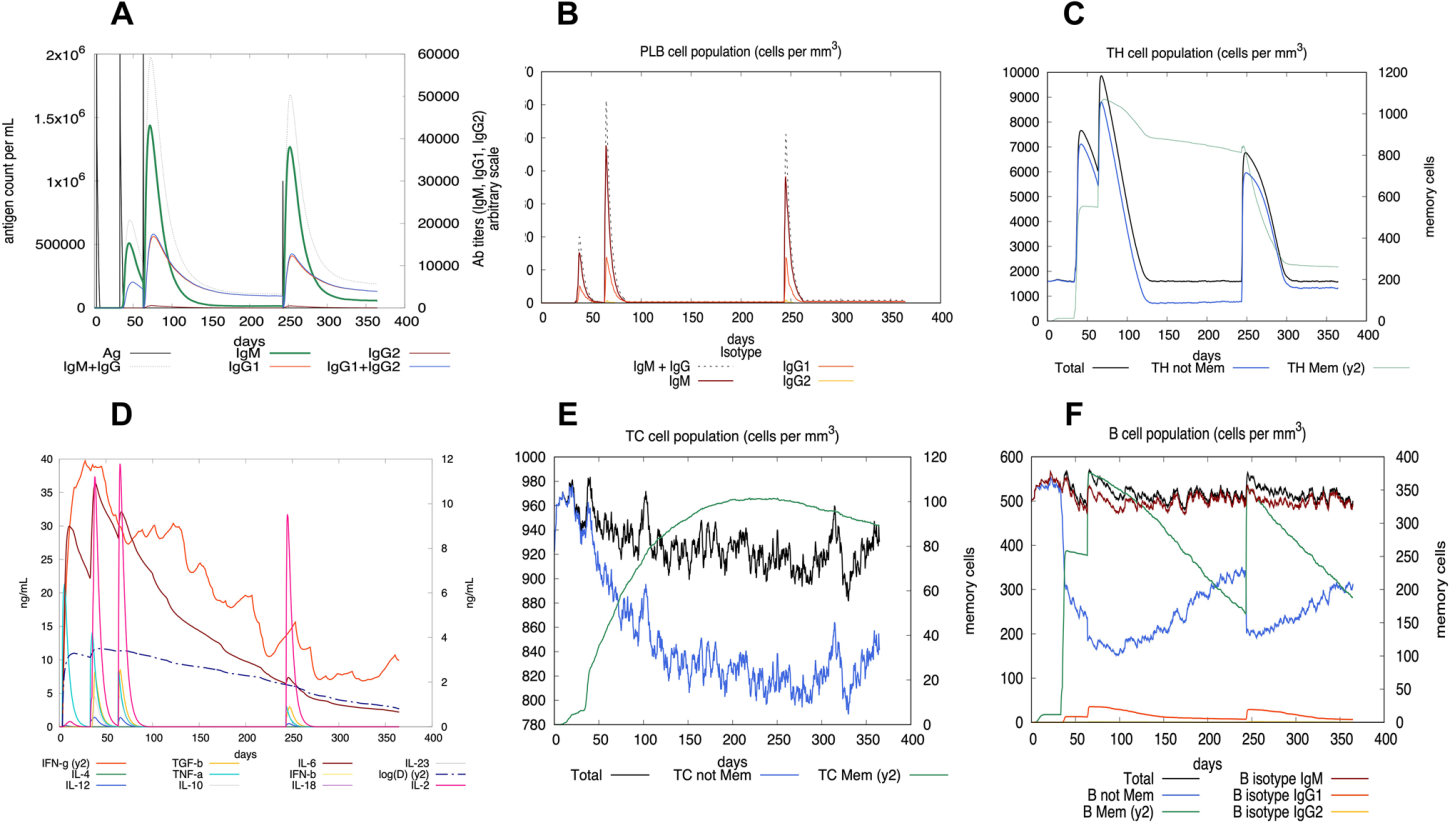
In silico simulation of immune response using vaccine as an antigen. (**A**) Antigen and immunoglobulins. (**B**) Plasma B cells. (**C**) TH cell population. (**D**) Cytokines and interleukins. (**E**) CTL population. (**F**) B cell population.

The C-IMMSIM server was used for generating an in silico immune response in order to assess the immunogenicity of the multi-epitope vaccine^75^. The vaccine administration protocol consisted in three injections at day 1, 30 and 60. A bacterial infection was started at day 240 in order to check the efficacy of the vaccination. As expected, the elicited secondary and tertiary responses were significantly higher when compared to the primary response. The decline in antigenic concentration with normal high levels of immunoglobulin activity (i.e., IgM, IgG1 and IgG2) is shown in panel A of Fig. 11. Additionally, a possible class switching and memory development was suggested due to the presence of multiple long lasting B cell isotypes (Fig. 11B). The pre-activation of TCs and a relatively high response in TH (helper) and TC (cytotoxic) cell populations was observed during vaccination (panels C and E of Fig. 11) (also Supplementary Fig. S16). During exposure, macrophages showed higher activities while the NK and dendritic cell activity was recorded to be consistent (Supplementary Fig. S16). The high levels of cytokines like IFN- γ and IL-2 supports the generation of an effective immune response (panel D of Fig. 11). The bacterial challenge following the vaccination shows the efficacy of the vaccine as the bacterial surge is virtually absent; mainly due to the protective action of high concentration of specific antibodies (panel A of Fig. 11). A control simulation was also performed consisting of an injection of replicating bacteria at time zero in absence of vaccination. The results indicate that without the preventive effect of the vaccine, there is an uncontrollable growth of the bacteria; even though the immune system faces the challenge by responding to the pathogen, it fails to control its growth due to the extreme replication speed (Supplementary Fig. S17).

## Discussion

During the last two decades, the morbidity and mortality rate of *Clostridium difficile* infection has vastly increased in both community and hospital settings^76^. Colonization is aided by dysbiosis of internal gut microbiota, which results mostly from elevated antibiotic therapy. Multiple proteins like CotE, SlpA and FliC are responsible for colonization, adherence and persistence of *Clostridium difficile*^19,20,23^. Hence, these proteins are selected for development of a multi-epitope vaccine candidate by using immunoinformatic approaches that is both time saving and cost effective^73,77–79^**.**

In this study, the designed multi-epitope vaccine not only elicits cell-mediated immunity, but also triggers humoral immune responses. Moreover, including only short immunogenic peptide sequences avoids allergic responses and prevents antigenic load^80,81^. The major advantages of the multi-epitope vaccine over conventional vaccines are as follows: (i) TCRs can identify several MHC Class I and Class II epitopes from diverse T cell subsets; (ii) the overlapping CTL, HTL and B cell epitopes have the capability to simultaneously activate humoral and cellular immune responses; (iii) linking a vaccine adjuvant guarantees long-lasting immune responses with improved immunogenicity; (iv) complications of the in vitro antigen expression and the complexity of pathogen culture can also be prevented^82–85^. The design of multi-epitope vaccines is a new field that has already gained significance, and the vaccines developed by this method have not only demonstrated protective immunity in vivo, but have also entered in phase I clinical trials^86–88^.

In this present study, an adjuvant, Cholera Toxin B (CTB) has been attached to N-terminal end of the vaccine using suitable linkers. Studies shows use of non-toxic CTB as a strong bacterial adjuvant in vaccines has successfully triggered an enhanced immune response by activation of CD4 + T cell responses^89–91^. Among various filters used for epitope screening, the CTL and HTL epitopes were checked for antigenicity, immunogenicity and promiscuity. In addition, overlapping CTL and HTL epitopes were also considered for final vaccine construction. The study confirms the antigenicity, immunogenicity and non-allergenicity of the final vaccine construct as predicted by VaxiJen v2.0, IEDB class I immunogenicity and AllerTOP servers, respectively^92^. Using the ExPASy ProtParam tool, the other physicochemical properties of the vaccine were analysed. The construct's molecular weight was 51,649.46 Da and the instability index was measured at 22.16, which classifies the vaccine as a stable protein. The vaccine's GRAVY index was − 0.162 (lower the GRAVY value, higher the solubility), which represents the polar identity of the vaccine and its efficient interaction with water, indicating high solubility^61^. The aliphatic index of 76.15 showed the protein as thermostable^59^. Half-life of 30 h in mammalian reticulocytes, > 20 h in yeast and > 10 h in *Escherichia coli* refers to the time taken by the protein to achieve 50% of its initial concentration after its synthesis in the cell. Ramachandran plot analysis of the vaccine using RAMPAGE showed 96.5%, 2.7% and 0.8% in favoured, allowed and outlier regions, respectively. An ERRAT score of 53.3865 further validated the overall quality of the vaccine. The ProSA web server predicted a Z-score of − 8.92, which indicates that the protein falls in a plot consisting of Z-scores of previously determined structures, which were solved using NMR and X-ray crystallography.

Our vaccine contains both CD8 + and CD4 + overlapping T cell epitopes which ensures stronger immune response in the body. Additionally, all the HTL epitopes were subjected to the prediction of IFN-γ, IL-2 and pro-inflammatory cytokine inducing epitopes. When the 9 HTL epitopes were screened for IL2Pred prediction, 5 epitopes were predicted to be strong inducers of interleukin-2 (Supplementary Table S9). Similarly, 5 epitopes were predicted to be inducing pro-inflammatory cytokines (including TNF) when subjected to the ProInflam web server (Supplementary Table S10). While checking for IFN-γ inducing HTL epitopes, the 15mer peptide sequences were subjected to the Predict Algorithm of the IFNepitope server, from which two epitopes were predicted as IFN-γ inducing (Supplementary Table S11). From these we can infer that few epitopes among our selected ones are responsible for inducing multiple immune factors. Thus, these epitopes might contribute for a robust immune response in the body.

Studies have shown that Toll-Like Receptor 4 (TLR4) recognizes spore protein CotE and surface layer protein SlpA whereas flagellar protein FliC is recognized by Toll-Like Receptor 5 (TLR5)^2,93–96^**.** TLR4 is expressed in multiple types of immune cells, such as immature dendritic cells, monocytes, granulocytes and macrophages^97^**.** In *C. difficile*-associated infection by SlpA, a significance of TLR4 is producing the immune response, which is required to clear the bacterium^94^. In addition, direct interaction of TLR4 with CTB is also responsible for CTB mediated activation of TLR4^98^. The ELISA-based assays have already shown that by binding directly to it, CTB can induce NF-κB activation in TLR4 receptor cells. Flagellin, on the other hand, may stimulate innate immunity by CCL20 and IL-8 production through the interaction of flagellar proteins with TLR5^99,100^**.** Also, a production of the inflammatory cytokine interleukin-22 (IL-22) is observed during TLR5 stimulation in body^96,101^. Molecular docking studies have assessed the vaccine's pattern of interaction with TLR4 and TLR5 (Figs. 5 and 6). The docking analysis of TLR4 with vaccine construct revealed that during this interaction there were 2 salt bridges and 19 hydrogen bonds formed. Similarly, docking analysis of TLR5 with the designed vaccine showed 3 salt bridges and 18 hydrogen bonds formed during the molecular interaction. Likewise, docking studies of the vaccine with MHC I and MHC II was also performed to check the binding of the vaccine with MHC molecules (Figs. 7 and 8). The docking analysis of MHC I with vaccine construct revealed that in this interaction there were 1 salt bridge and 17 hydrogen bonds formed; and 1 salt bridge and 13 hydrogen bonds were formed in the docked structure of MHC II and vaccine. Molecular Dynamics Simulation (MDS) of the vaccine-TLR complexes was performed for verifying the stability and flexibility of the complexes in various experimental conditions like temperature and pressure. A trajectory run of 18 ns showed stable interactions between the vaccine and the TLRs during the simulation run with limited fluctuations (Fig. 9). The RMSF graphs generated showed regions with high peaks which hint towards high flexibility of the vaccine-TLR complexes (Fig. 9). The immune simulation studies conducted on the designed vaccine construct confirmed its ability to clear the antigen on secondary exposure by eliciting specific immune responses (Fig. 11).

A similar array of in silico analysis was performed by Bazhan and his colleagues where they have designed a T-cell multi epitope vaccine against Ebola virus. The T cell epitopes were predicted using the same server, IEDB—Immune Epitope Database; and the vaccine candidate constructed using the suitable epitopes were found to be immunogenic when expressed in mice^38^. Similarly, Forountan and his co-workers have designed their experiments, where they have assessed the allergenicity and other physicochemical properties of their vaccine candidate against *Toxoplasma gondii* by a series of immunoinformatics tools, which are similar to the ones we have used, and their wet lab validation suggested that the multi-epitope vaccine was able to trigger strong humoral and cellular responses in mice^39^. The antigenicity, allergenicity and physicochemical scores obtained by our vaccine candidate were comparable, and in fact few values like antigenicity, aliphatic index, instability index and Ramachandran plot have better scores than the values reported by Forountan and his team in their published work. These studies strengthen the fact that the vaccine candidates designed in silico using computational tools can be a successful strategy for designing an efficient vaccine candidate against diseases. Similar immunoinformatics concepts have been applied in designing multi-epitope vaccines against SARS-CoV-2^73^**,** Dengue^102^**,** Nipah Virus^103^, Malaria^104^**,** Hendra virus^105^, and many more. Additionally, development of vaccine candidates against cancer antigens has also been seen^35,106^. The CTL, HTL, and IFN-γ epitopes found in the vaccine are capable of activating the regulation of the respective immune cells of the host, which may induce the triggering of other immune cells through complex signalling. The vaccine candidate proposed in the study is intended to be delivered via injection following an intramuscular route. Intramuscular administration (I.M) is preferred for the delivery of the vaccine candidate since it is easy to perform and well tolerated, with a low risk for adverse reactions at the site of injection and is the most commonly used route for licensed vaccines^107^. Recent studies also show that intramuscular immunization is a promising strategy for induction of strong systemic CTL response^108^. In addition, CDC recommends that vaccines containing an adjuvant should be injected into a muscle rather than any other routes since they can cause local irritation, induration, skin discolouration, inflammation, and granuloma formation^109,110^.

## Materials and methods

### Sequence retrieval and PDB structure retrieval

The FASTA sequences of SlpA, FliC and CotE proteins were retrieved from NCBI database having the accession numbers CAJ69681.1, AJP09935.1 and CAJ68298.1, respectively. The PDB structures of TLR4, TLR5, MHC I and MHC II receptors were retrieved from Protein Data Bank with accession ID 3FXI, 3J0A, 1I1Y and 1KG0, respectively.

### T cell epitope and IFN-γ epitope prediction

NetCTL 1.2 server was used for predicting the 9-mer long CTL epitopes with 54–89% sensitivity and 94–99% specificity^43^. These epitopes were recognized by commonly occurring HLA class I supertypes in human population, which are: A1, A2, A3, A24, A26, B7, B8, B27, B39, B44, B58 and B62. In addition, Immune Epitope Consensus (IEDB) tool also detected CTL epitopes which were recognized by other HLA class I alleles^44^. In contrary, 15-mer HTL epitopes, having an affinity to HLA class II alleles were predicted by NetMHCII pan 3.2 server^111^. Based on the idea of percentile rank given by the server, all the epitopes are classified as strong, intermediate and non-binders to HLA alleles with threshold values of 2, 10 and > 10% respectively. All the predicted epitopes were verified for antigenicity by VaxiJen v2.0 server^58^, along with immunogenicity check using IEDB class I immunogenicity web server^112^.

IFN-γ epitopes from the chosen bacterial proteins were predicted using the Scan Algorithm of the IFNepitope server^46^. This server uses various approaches like as machine learning strategy, motive-based analysis and accuracy hybrid approach for epitope prediction and has a maximum accuracy of 81.39%^46^.

### Multi-epitope vaccine construction, modelling and validation of vaccine structure

The selected CTL, HTL and IFN-γ epitopes screened from the SlpA, CotE and FliC proteins were linked together with GPGPG linkers. Also, EAAAK linker was used to attach Cholera Toxin B (CTB) adjuvant to the vaccine at the N terminal. The designed vaccine linear construct was subjected to tertiary modelling by trRosetta webserver^53^. The resulted 3D model was validated using ERRAT, Rampage and ProSA webservers^54–56^ and these servers were used for the prediction of ERRAT score, Ramachandran plot and Z-score of the protein 3D model.

### Antigenicity, immunogenicity, allergenicity and physicochemical properties of the vaccine model

To predict the antigenicity of the vaccine, VaxiJen v2.0 server was used. Sequences with a score more than 0.4 threshold value are considered antigenic. VaxiJen v2.0 server uses viral and bacterial databases which contain 100 identified antigens and 100 non-antigens. Models are evaluated using these datasets with 70–89% accuracy, using internal leave-one-out cross-validation and external validation. The immunogenicity of the vaccine construct was checked using IEDB class I immunogenicity server, where more the score, more is the immunogenicity. Similarly, the allergenicity of the vaccine was verified using AllerTOP server, which employs auto-cross-covariance (ACC) grouping of protein sequences into uniform equal-length vectors^92^. To evaluate other physicochemical properties like aliphatic index, theoretical pI, half-life, molecular weight, instability index and GRAVY score of the vaccine, ExPASy ProtParam tool was used^60^. The vaccine construct was also checked by SignalP4.1 and TMHMM server v2.0 for the presence of any signal peptides and transmembrane helices, respectively^113,114^.

### B cell epitope, IL2 inducing, pro-inflammatory cytokine inducing and IFN-γ inducing HTL epitope predictions

The presence of B cell epitopes in vaccine construct was confirmed using ElliPro webserver from IEDB^115^. The server predicted continuous/linear and discontinuous/conformational B cell epitopes in the vaccine.

The IL-2 epitopes were predicted by IL2Pred webserver (https://webs.iiitd.edu.in/raghava/il2pred/). The server predicts the IL-2 inducing positive and negative epitopes based on the derived epitopes from IEDB dataset. Similarly, Pro-inflammatory cytokine inducing CD4 + T cell epitopes were predicted by using the ProInflam web server (http://metagenomics.iiserb.ac.in/proinflam/) This server identifies the epitopes which are pro-inflammatory cytokine inducers and can induce cytokines like TNF, IL-18, IL-12, IL-23^63^. The IFN-γ inducing HTL epitopes, selected for the vaccine were identified using IFNepitope server’s Predict Algorithm^46^. This server identifies the IFN-γ epitopes on the basis of their respective scores, where a positive score indicates the epitopes to be IFN-γ secreting^46^.

### Population coverage

The population coverage for the epitopes of the designed vaccine was checked for the world population, United States, Europe, China, South Asia and Oceania using IEDB population coverage analysis tool (http://tools.iedb.org/population/)^65^. All the parameters for the study were kept at default and the coverage was checked against MHC class I and MHC class II alleles.

### Docking of the vaccine construct with TLR4, TLR5, MHC I and MHC II and binding affinity

The vaccine was subjected to molecular docking studies for better understanding of its interaction with Toll-Like Receptors and MHC molecules, which aid in better and more stable immune response. For this interaction study, firstly active and passive residues for the vaccine and receptor molecules used in this study are predicted using CPORT^116^ and HADDOCK 2.4 server then helps in performing docking of the vaccine with TLR4 and TLR5 as well as MHC I and MHC II^66^. Among the docked clusters, the best cluster was chosen based on lowest HADDOCK score. The chosen model was subjected to refinement by HADDOCK Refinement Server. The final docked structures after refinement were subjected to PRODIGY web-server (https://bianca.science.uu.nl/prodigy/) for prediction of binding energy^72^. In addition, PDBsum was used to map the interacting residues between the docked chains (i.e., the vaccine, the TLRs and the MHCs)^117^.

### Energy minimization and molecular dynamics simulation

#### Vaccine-TLR complexes

For performing Molecular Dynamics Simulation (MDS) and energy minimization of the vaccine-TLR complexes, a Linux-based command line program, GROMACS (GROningen MAchine for Chemical Simulations) was used^118^. In order to mimic the experimental conditions, the complexes were subjected to MDS. Physical conditions like pressure and temperature were imitated in this study using the canonical ensemble NVT and the isobaric and isothermal ensemble NPT. Mimicking the experimental conditions, temperature set for the desired simulation was 300 K. OPLS-AA (Optimized Potential for Liquid Simulation-All Atom) force field constraint for energy minimization and equilibration was used to generate a topology file used for the simulation. In order to obtain the periodic image of the complex structures 2 nm apart, the structures were positioned at a distance of 1 nm from the edge of the cube filled with water molecules. An equilibrated three-point water model, spc216 was used as the solvent to simulate the vaccine with periodic boundary conditions. After neutralizing the system by addition of charged ions, the net charge of the vaccine-TLR constructs were evaluated. The system’s temperature and pressure was also stabilized by performing the NVT and NPT equilibration for 100 pico-seconds (ps) consisting of 50,000 steps. In order to find the Root Mean Square Deviation (RMSD) of backbone and Root Mean Square Fluctuation (RMSF) of side chain, a 18 ns Molecular Dynamics (MD) simulation run was carried out for the energy minimised structure of the complexes. To analyse the compactness of the protein structure, the radius of gyration was also plotted. Xmgrace, software based on Linux, was employed to visualize the simulation graphs generated after the simulation runs^119^.

### Codon optimization and in silico cloning of vaccine

The Java Codon Adaptation Tool (JCat) was used to optimize codon and for reverse translation that generated the vaccine's cDNA sequence that can be used for an efficient expression in *E. coli* K-12 strain^74^. Additionally, the SnapGene software (from Insightful Science; available at snapgene.com) was used to insert the optimized multi-epitope vaccine’s DNA sequence into the pET28a (+) vector.

### Immune simulation

The C-IMMSIM server (https://kraken.iac.rm.cnr.it/C-IMMSIM/) was used to perform *in silico* immune simulation in order to characterize the immunogenicity and immune response of the engineered peptide^75^. C-IMMSIM is an agent-based model that uses position-specific scoring matrices (PSSM) derived from machine learning techniques for predicting immune interactions. For most of the vaccines currently in use, 4 weeks is the minimum recommended time between the first and second dose^120^. The entire simulation ran for 1400 time steps which are about 15 months (a time step is about 8 h). Three peptide injections were given at day 1, day 30 (i.e., after 1 month) and day 60 (i.e., after 2 months), followed by the bacterial challenge at day 240 (i.e., after 8 months = 6 months after end of vaccination) in order to check the efficacy of the vaccination process.

## Conclusion

Increased repetitive occurrence of CDI is observed in patients who receive antibiotic therapies in hospital environment. No proper prevention except antibiotic treatments and toxoid vaccines prescribed after incidence of *Clostridium difficile* infection is available for patients. Toxoid vaccines act on the secreted toxin A and toxin B after *C. difficile* colonize in gastro-intestinal tract. However, the concern regarding bacterial attachment, colonization and spore formation remains untouched. The in silico approaches can be used to develop an efficient vaccine using target antigenic proteins in less time and at minimal cost. In this study, immunoinformatic tools have been applied for constructing a multi-epitope chimeric vaccine candidate against *Clostridium difficile*. The designed vaccine showed both antigenic as well as immunogenic properties with no allergenic responses. Strong immune responses were validated using Immune Simulation studies. Also, detailed molecular docking and molecular dynamics simulation studies have shown the vaccine to be stable.

## Supplementary Information

Supplementary Information 1.

Supplementary Information 2.

Supplementary Information 3.
