# Supplementary material for: In silico designing of vaccine candidate against Clostridium difficile
Source: Sci Rep. 2021 Jul 9;11:14215. doi: 10.1038/s41598-021-93305-6 (PMC8271013; doi:10.1038/s41598-021-93305-6)
Supplement: Supplementary file 1 — Supplementary Information 1. [file 41598_2021_93305_MOESM1_ESM.pdf]

In silico designing of vaccine candidate against *Clostridium difficile*

Srijita Basak[1*], Debashrito Deb[1*], Utkarsh Narsaria[1*], Tamalika Kar[1*], Filippo Castiglione[2], Indraneel Sanyal[1], Pratap D. Bade[1†], Anurag P. Srivastava[1†]

[1]Biopharmaceutical Development Department

Syngene International Limited, Bangalore

India

[2]Institute for Applied Computing (IAC),

National Research Council of Italy,

Rome, Italy

*These authors contributed equally to this work.

†Corresponding Author

Anurag P. Srivastava: Anurag.Srivastava@syngeneintl.com, anuiitkgp@gmail.com

Phone: +91-9108720555

ORCID ID: 0000-0003-3493-1375

Pratap D. Bade: Pratap.Bade@syngeneintl.com

Phone: +91-8884522554

Supplementary Figures

Supplementary Figure S1: Signal Peptide

Measure	Position	Value	Cutoff	signal peptide?
max. C	40	0.120		
max. Y	50	0.124		
max. S	38	0.163		
mean S	1-49	0.103		
D	1-49	0.116	0.450	NO

Name=Sequence SP='NO' D=0.116 D-cutoff=0.450 Networks=SignalP-TM

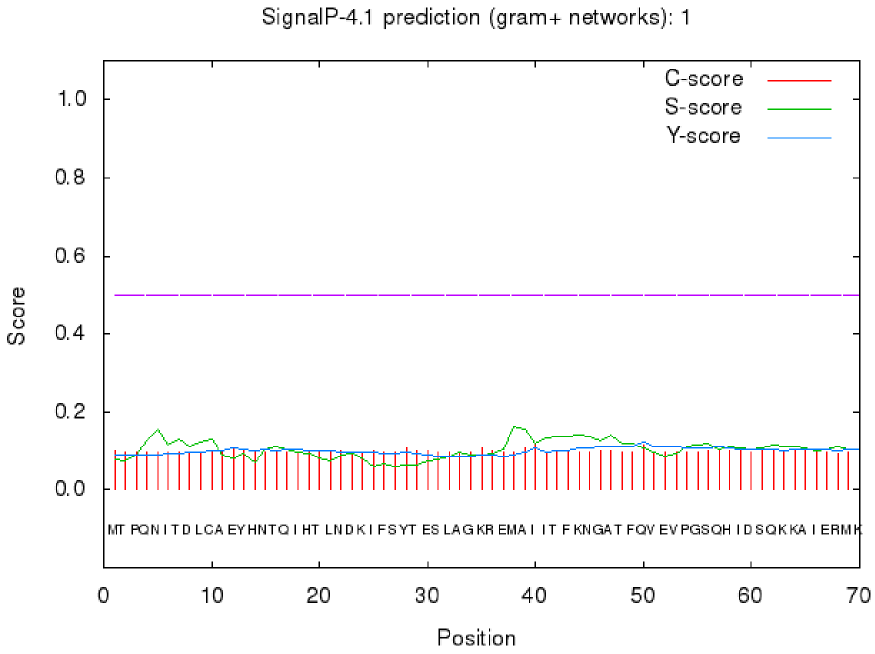

Supplementary Figure S2: Transmembrane Helix

WEBSEQUENCE Length: 512
WEBSEQUENCE Number of predicted TMHs: 0
WEBSEQUENCE Exp number of AAs in TMHs: 0.15955
WEBSEQUENCE Exp number, first 60 AAs: 0
WEBSEQUENCE Total prob of N-in: 0.00704
WEBSEQUENCE   TMHMM2.0   outside   1   512

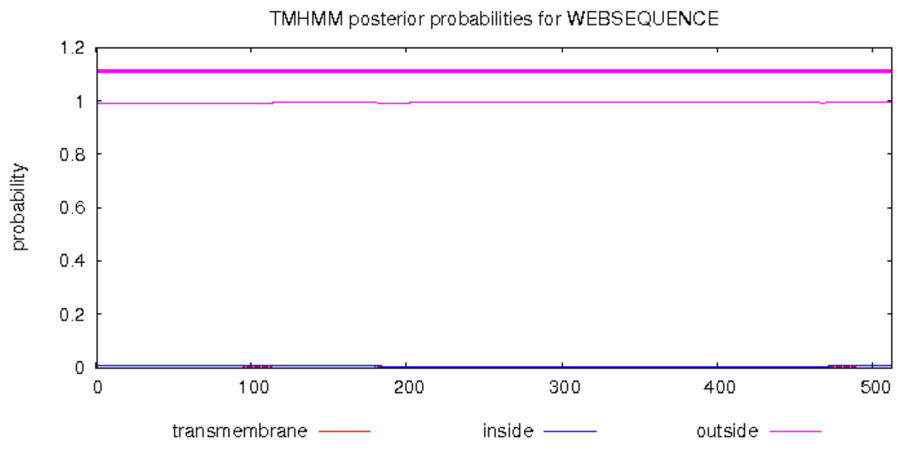

Supplementary Figure S3: B Cell Epitopes

A. Continuous Epitopes predicted by ElliPro and visualized using PyMol. Epitopes are shown in red colour and the vaccine is shown in yellow colour.

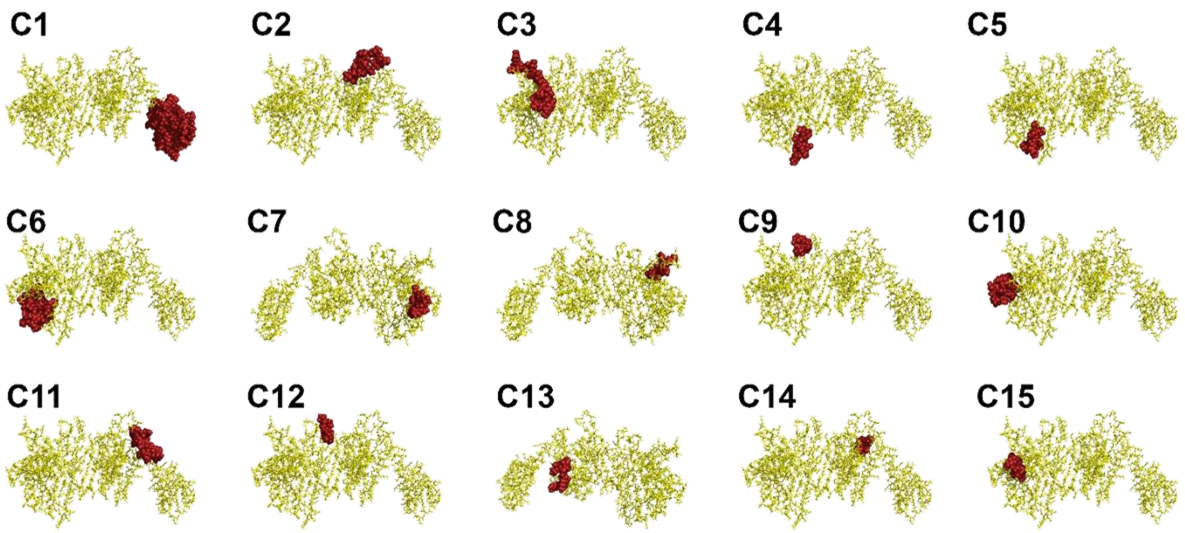

B. Discontinuous Epitopes predicted by ElliPro and visualized using PyMol. Epitopes are shown in green colour and the vaccine is shown in blue colour.

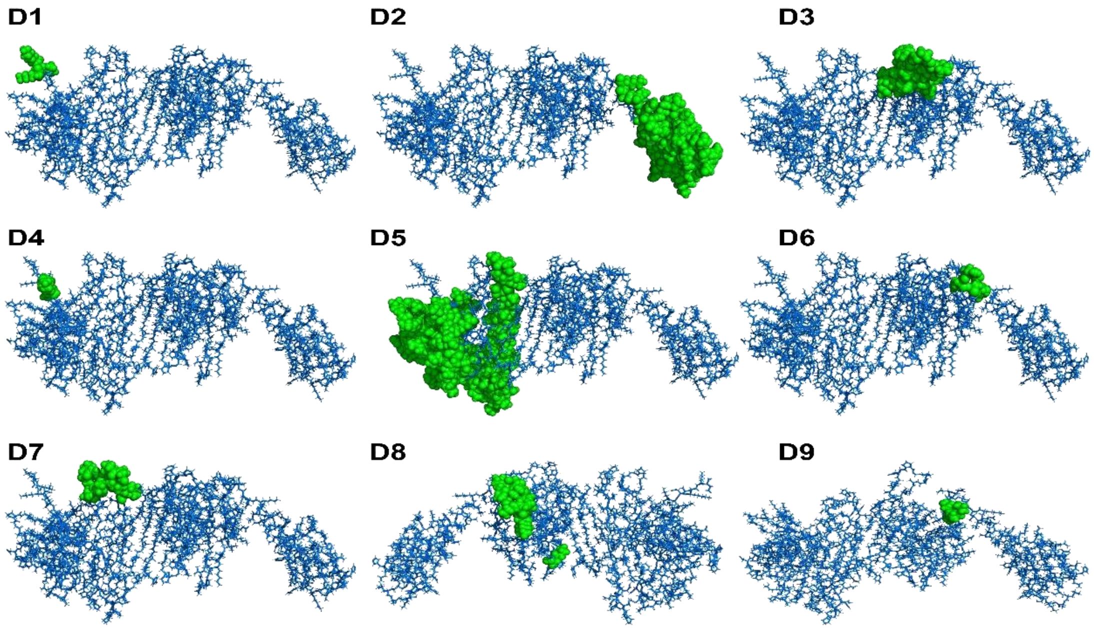

Supplementary Figure S4: **Plots showing population coverage.**

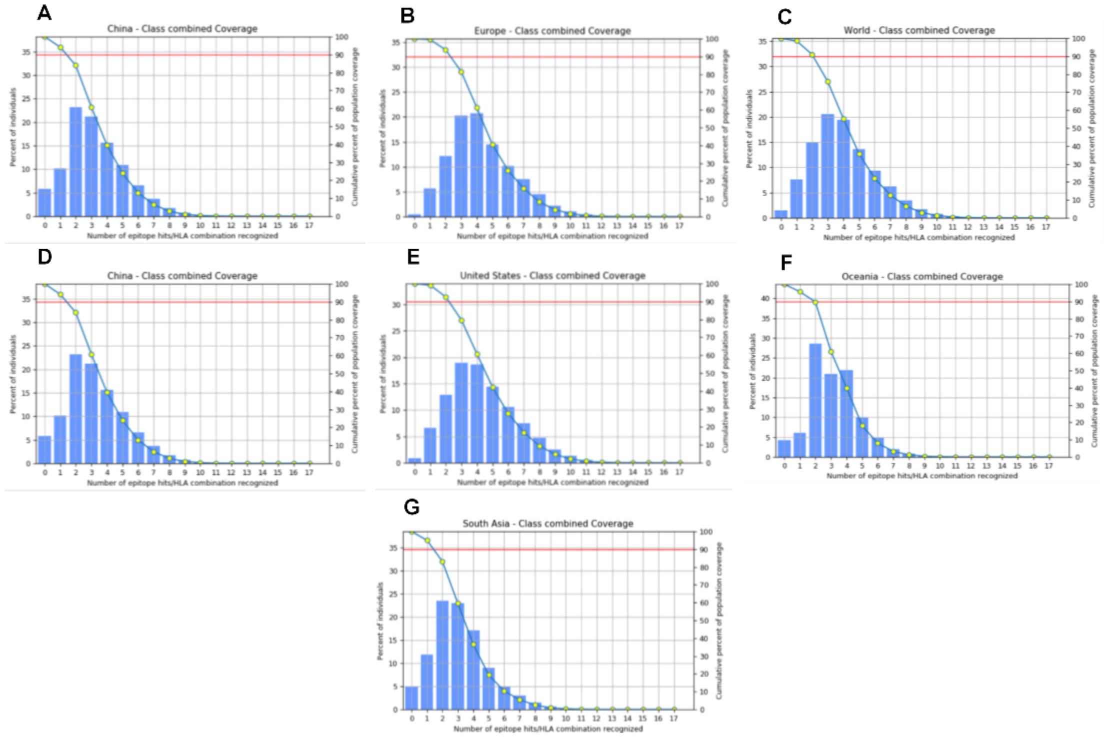

Supplementary Figure S5: Overview of docked complexes of individual CTL epitopes with MHC I receptor. 'c' represents the CTL epitopes docked with MHC class I receptor 'F' denotes epitopes derived from FliC protein, 'C' denotes epitopes from CotE protein and 'S' denotes the epitopes from SlpA protein. The arrangement of the epitopes is based on their position in the vaccine construct.

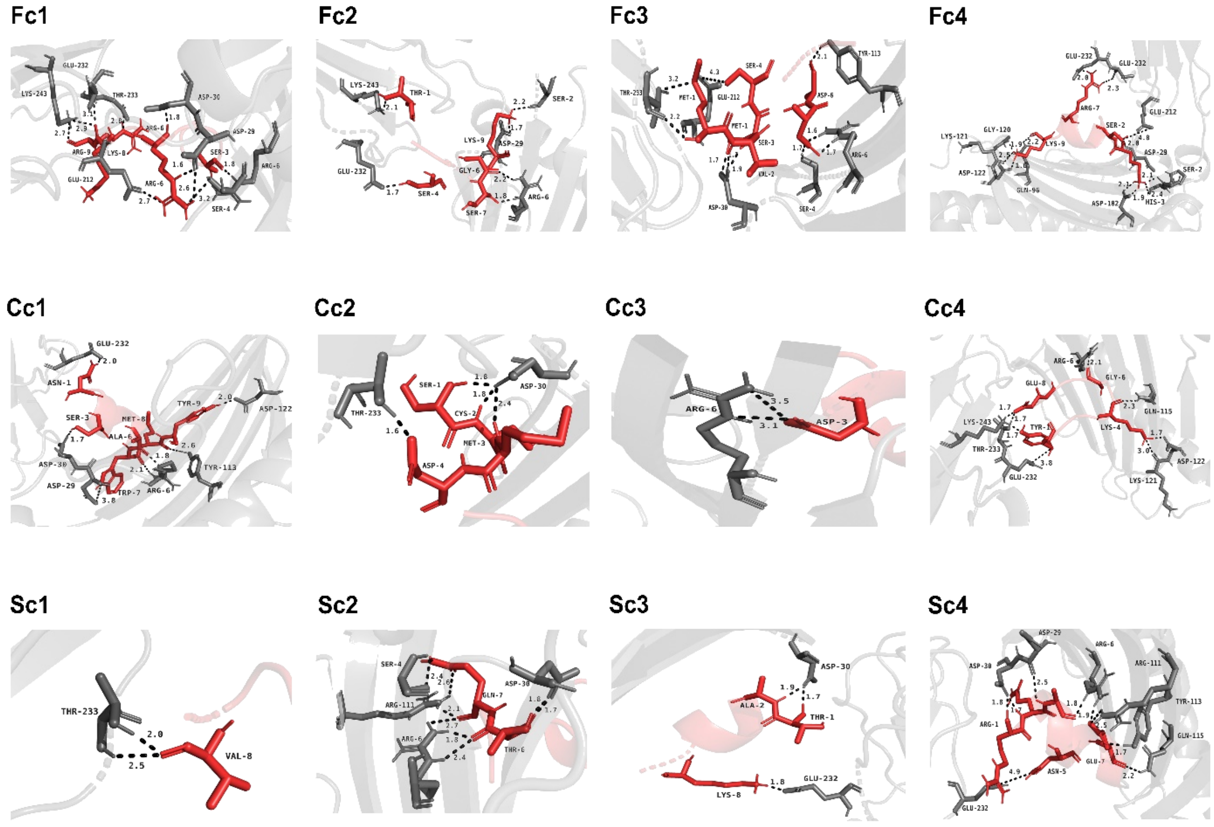

Supplementary Figure S6: Overview of docked complexes of individual HTL epitopes with MHC II receptor. 'h' represents the HTL epitopes docked with MHC class II receptor. All the epitopes are depicted in red colour spheres. 'F' denotes epitopes derived from FliC protein, 'C' denotes epitopes from CotE protein and 'S' denotes the epitopes from SlpA protein. The arrangement of the epitopes is based on their position in the vaccine construct.

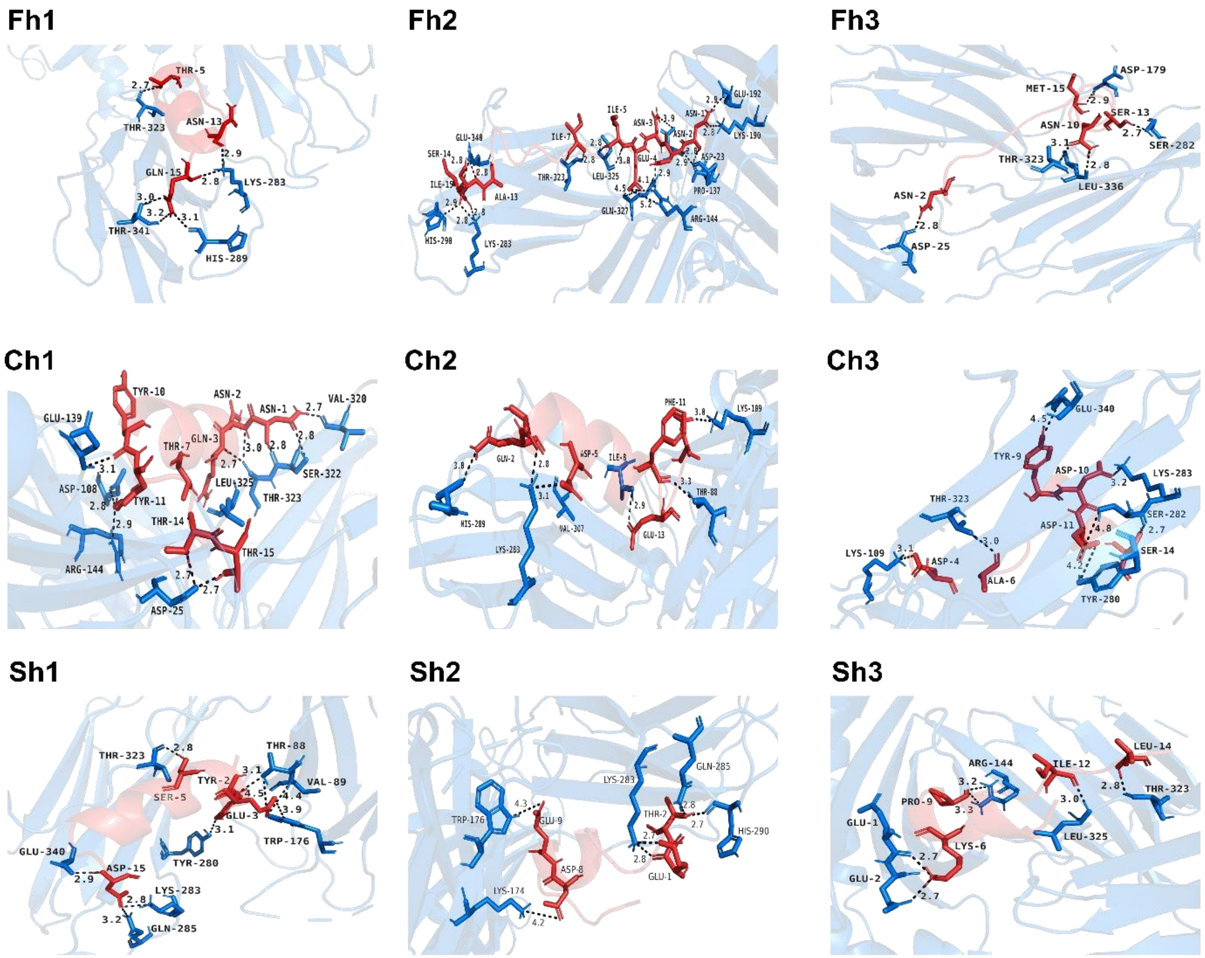

Supplementary Figure S7: The results and graphics presented below are based on HADDOCK generated water-refined models of TLR 4 with vaccine construct

i-RMSD -> interface-RMSD calculated on the backbone (CA,C,N,O,P) atoms of all residues involved in intermolecular contact using a 10Å cutoff.

l-RMSD -> ligand-RMSD calculated on the backbone atoms (CA,C,N,O,P) of all (N>1) molecules after fitting on the backbone atoms of the first (N=1) molecule.

FCC -> Fraction of common contacts. The intermolecular contacts are defined based on the best HADDOCK model using a 5Å cutoff.

a.u. -> Arbitrary Units.

The cluster averages and standard deviations are indicated by colored dots with associated error bars. The average values are calculated on the best 4 structures of each cluster (based on the HADDOCK score).

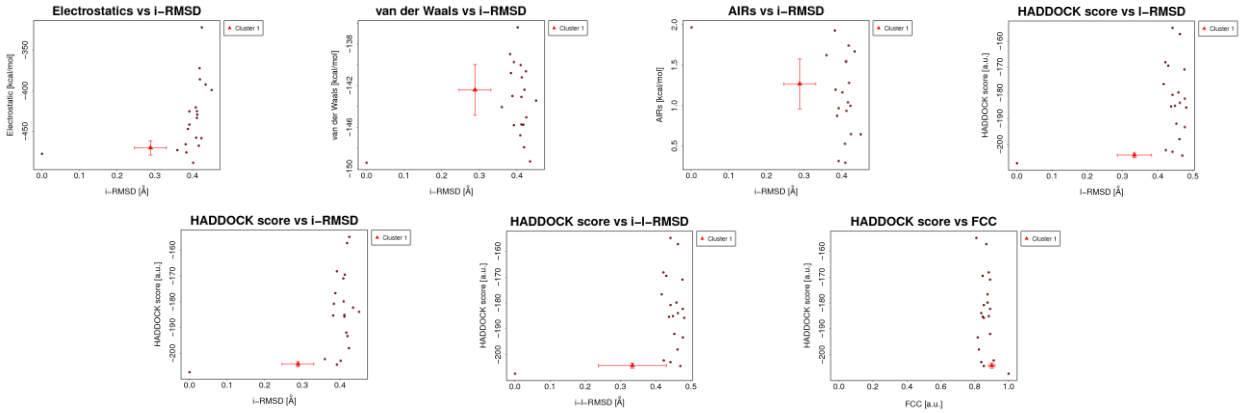

Supplementary Figure S8: Overview of docked complex of TLR4 and vaccine. Hydrogen bonds are shown with black dashes.

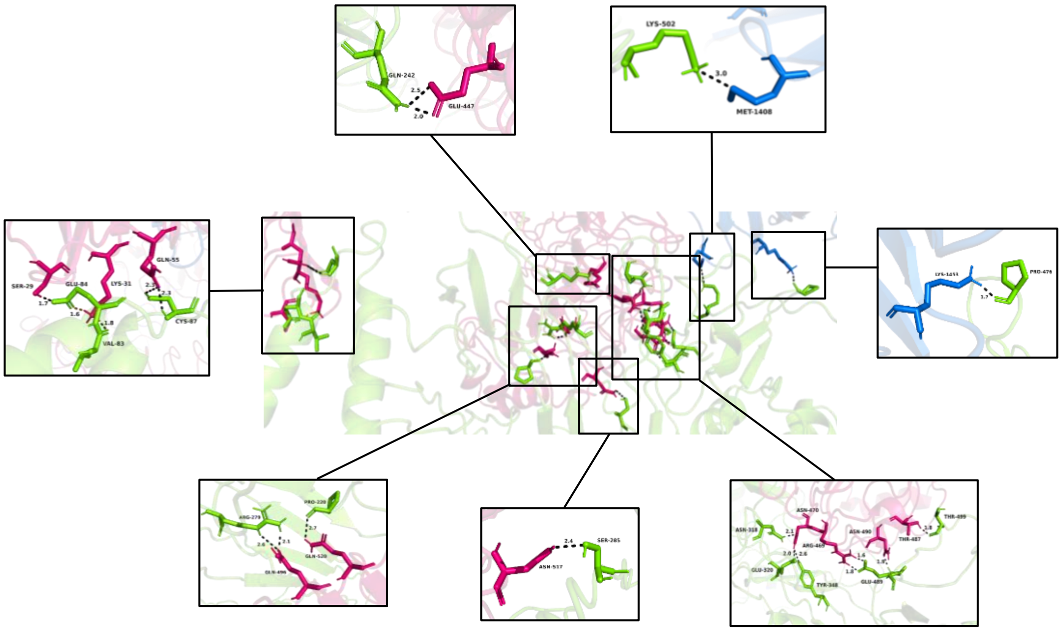

Supplementary Figure S9: The results and graphics presented below are based on HADDOCK generated water-refined models of TLR5 with vaccine construct.

i-RMSD -> interface-RMSD calculated on the backbone (CA,C,N,O,P) atoms of all residues involved in intermolecular contact using a 10Å cutoff.

l-RMSD -> ligand-RMSD calculated on the backbone atoms (CA,C,N,O,P) of all (N>1) molecules after fitting on the backbone atoms of the first (N=1) molecule.

FCC -> Fraction of common contacts. The intermolecular contacts are defined based on the best HADDOCK model using a 5Å cutoff.

a.u. -> Arbitrary Units.

The cluster averages and standard deviations are indicated by colored dots with associated error bars. The average values are calculated on the best 4 structures of each clusters (based on the HADDOCK score).

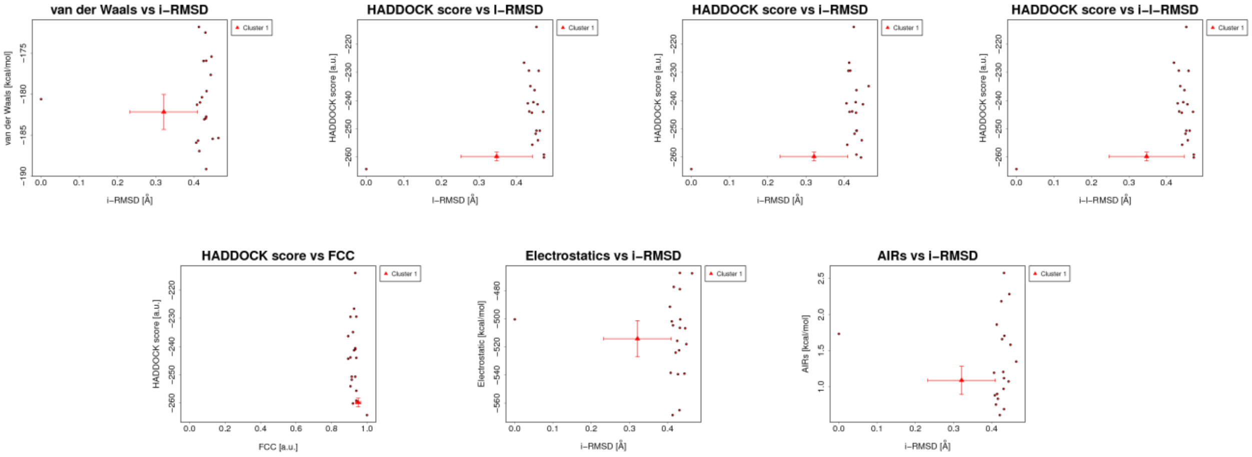

Supplementary Figure S10: Overview of docked complex of TLR5 and vaccine. Hydrogen bonds are shown with black dashes and salt bridge is shown with orange dashes.

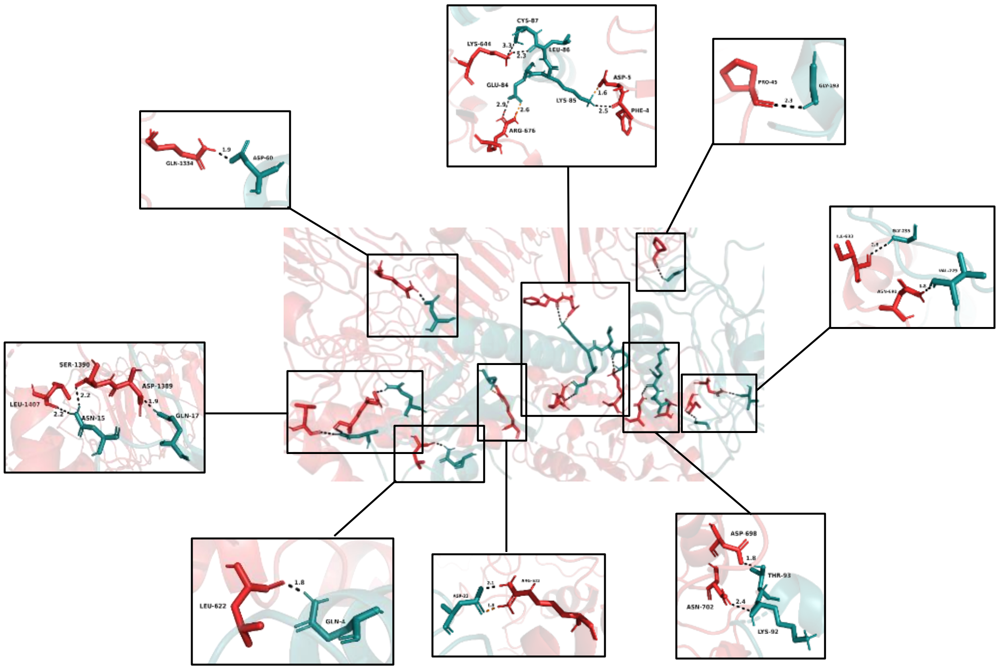

Supplementary Figure S11: The results and graphics presented below are based on HADDOCK generated water-refined models of MHC I with vaccine construct.

i-RMSD -> interface-RMSD calculated on the backbone (CA,C,N,O,P) atoms of all residues involved in intermolecular contact using a 10Å cutoff.

l-RMSD -> ligand-RMSD calculated on the backbone atoms (CA,C,N,O,P) of all (N>1) molecules after fitting on the backbone atoms of the first (N=1) molecule.

FCC -> Fraction of common contacts. The intermolecular contacts are defined based on the best HADDOCK model using a 5Å cutoff.

a.u. -> Arbitrary Units.

The cluster averages and standard deviations are indicated by colored dots with associated error bars. The average values are calculated on the best 4 structures of each clusters (based on the HADDOCK score).

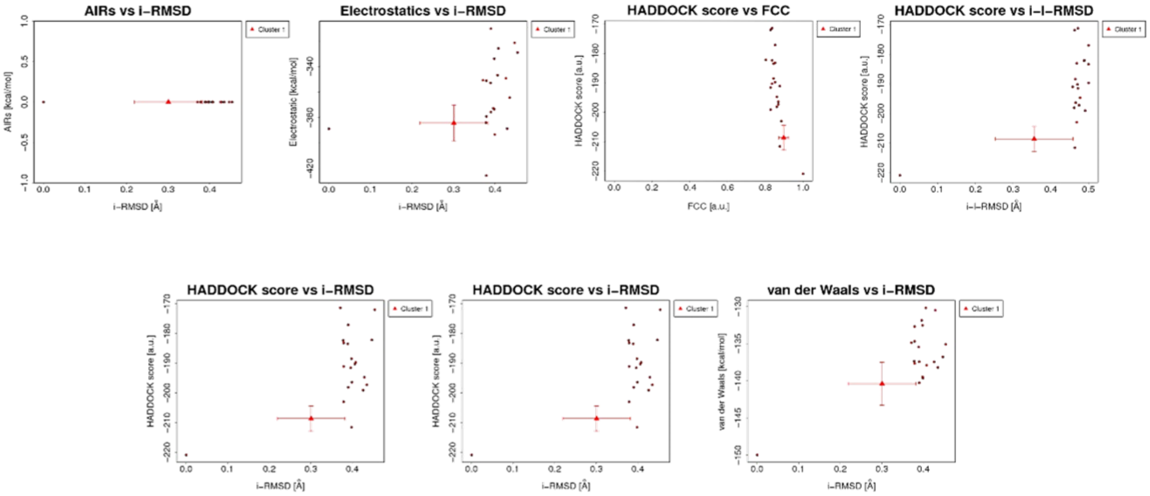

Supplementary Figure S12: The results and graphics presented below are based on HADDOCK generated water-refined models of MHC II with vaccine construct.

i-RMSD -> interface-RMSD calculated on the backbone (CA,C,N,O,P) atoms of all residues involved in intermolecular contact using a 10Å cutoff.

l-RMSD -> ligand-RMSD calculated on the backbone atoms (CA,C,N,O,P) of all (N>1) molecules after fitting on the backbone atoms of the first (N=1) molecule.

FCC -> Fraction of common contacts. The intermolecular contacts are defined based on the best HADDOCK model using a 5Å cutoff.

a.u. -> Arbitrary Units.

The cluster averages and standard deviations are indicated by colored dots with associated error bars. The average values are calculated on the best 4 structures of each clusters (based on the HADDOCK score).

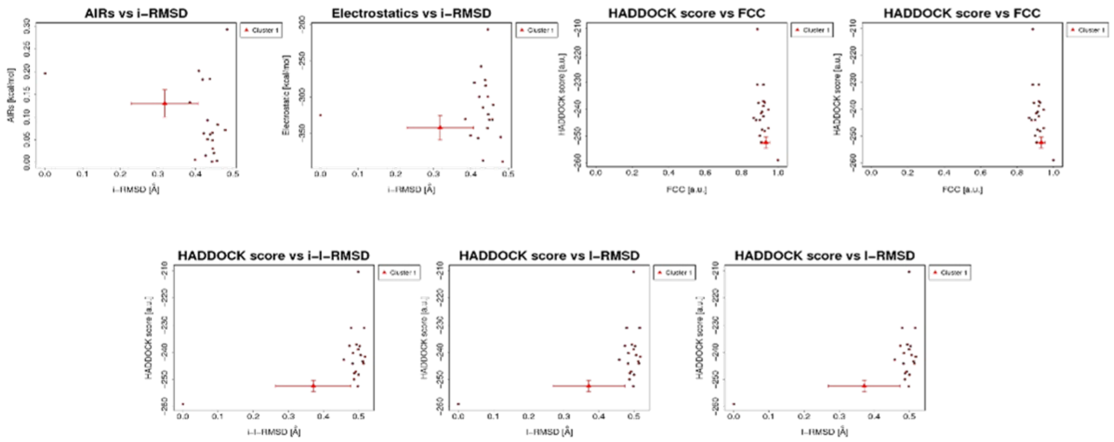

Supplementary Figure S13: Overview of docked complex of MHC I and vaccine. Hydrogen bonds are shown with black dashes and salt bridge is shown with orange dashes.

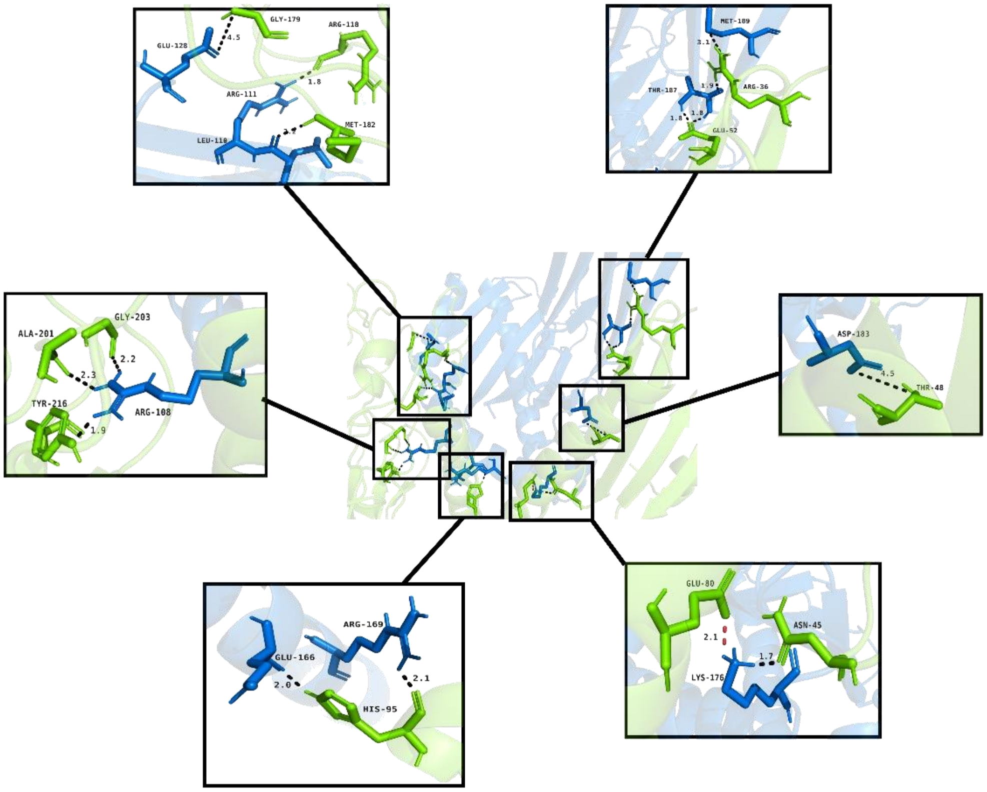

Supplementary Figure S14: Overview of docked complex of MHC II and vaccine. Hydrogen bonds are shown with black dashes and salt bridge is shown with orange dashes.

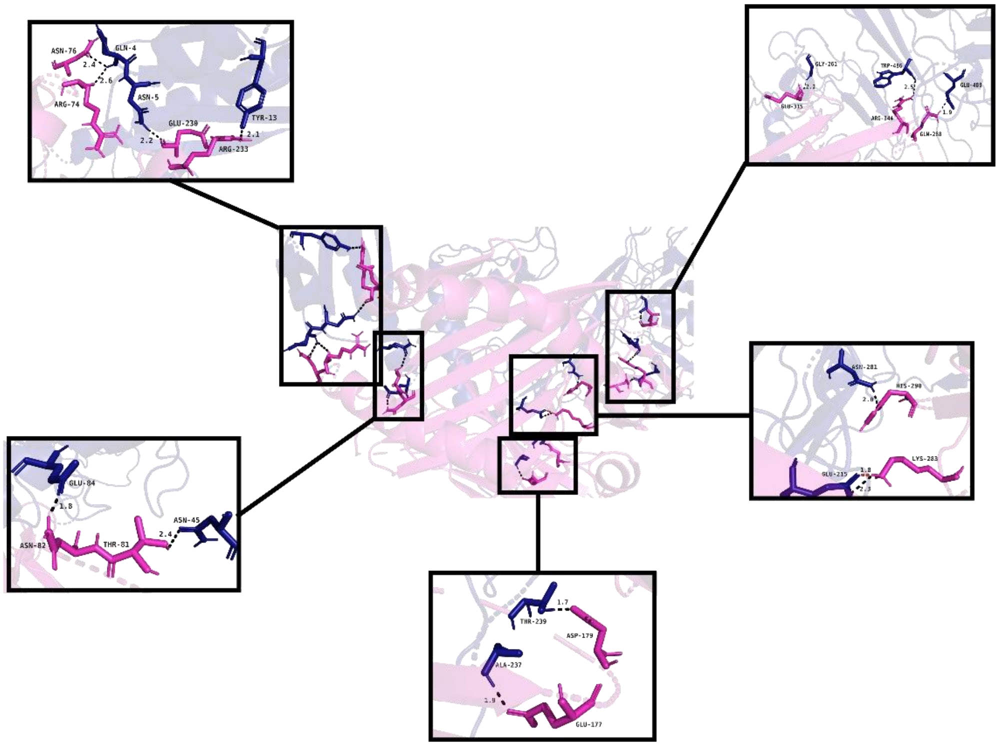

Supplementary Figure S15: Graphs for molecular dynamic simulation of the Vaccine- TLR complexes showing temperature, density, pressure and radius of gyration.

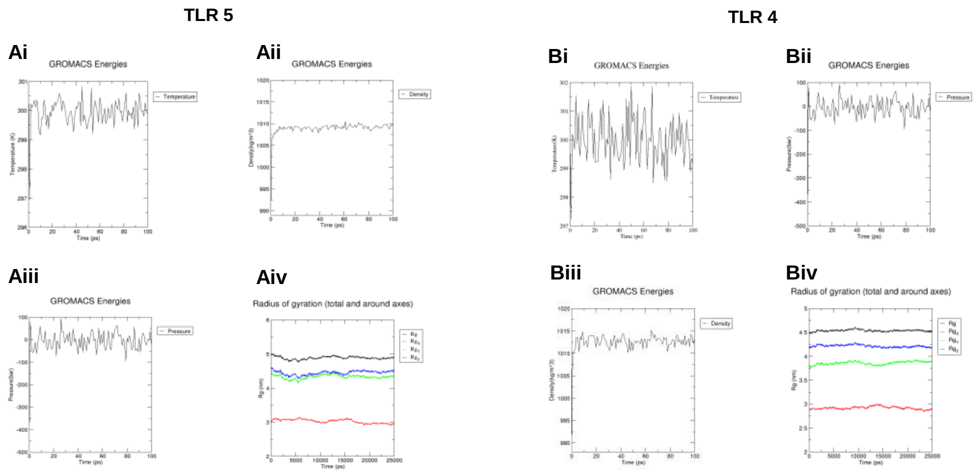

Supplementary Figure S16: Plots showing detailed dynamics of immune simulation variables.

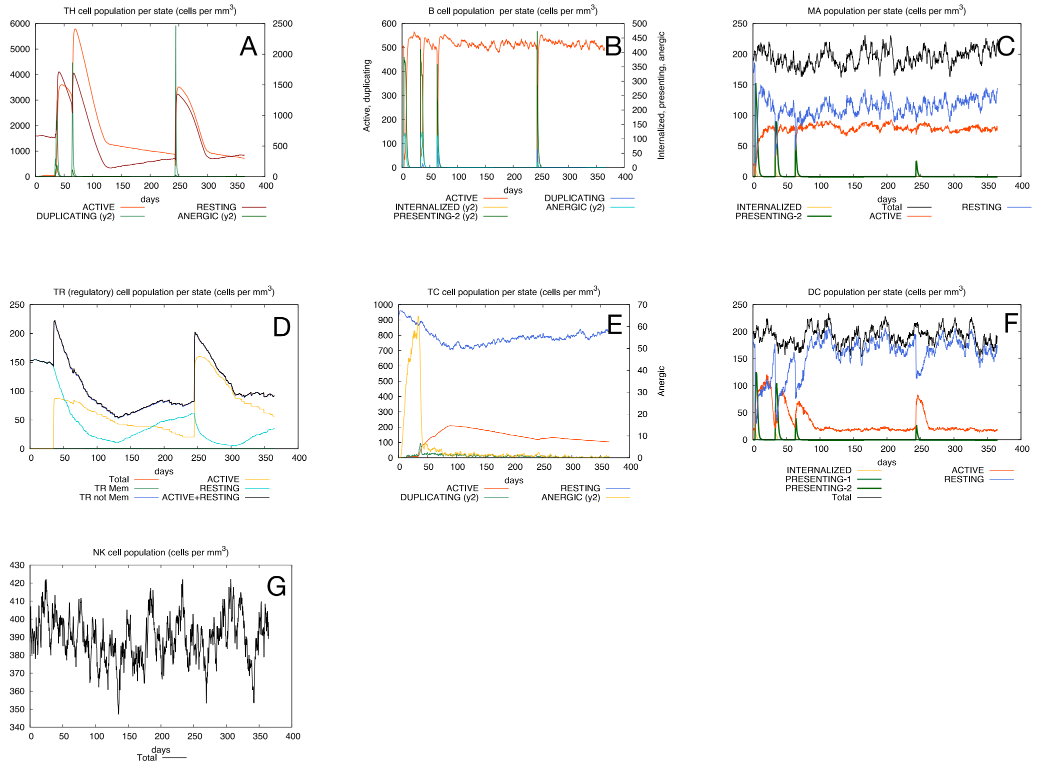

Supplementary Figure S17: Plot showing control study for immune simulation of the vaccine. Without the immune memory elicited by the vaccine, the immune response is weak and slow, as shown by the count difference from baseline of B and helper T cells on the right y-scale, and the bacteria can growth rapidly as shown by the exponential rise on the left y-scale.

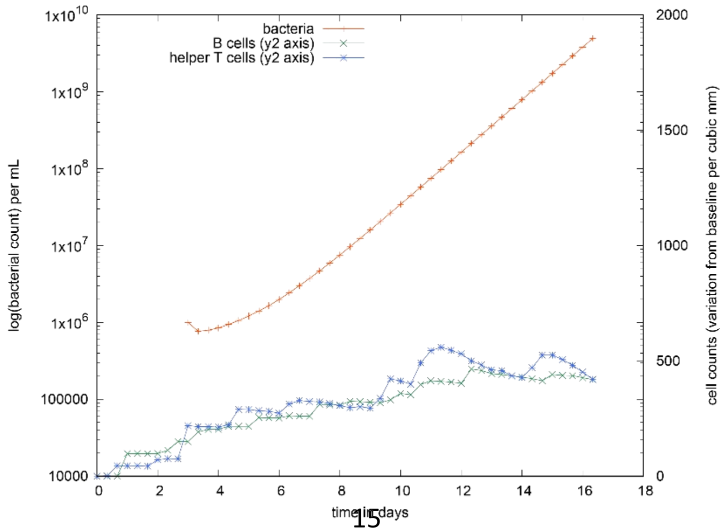

Supplementary materials

Supplementary material SM 1: Vaccine constructs

The underlined portion marks the CTB adjuvant, blue color denotes epitopes from CotE protein, orange color denotes epitopes from FliC protein and epitopes SlpA protein are marked with green. The GPGPG and EAAAK linkers are marked black.

1. **CotE → FliC → SlpA**
MTPQNITDLCAEYHNTQIHTLNDKIFSYTESLAGKREMAIITFKNGATFQVEVPGSQHIDSQKKAIERMKDTLRIAYLTEAKVEKLCVWNNKTPHAIAAISMANEAAAKNSSHLAWMYGPGPGSCMDWYLCFGPGPGLLDAVIFAFGPGPGYINKNGYEYGPGPGLSSGVRIKRGPGPGTTASIGSMKGPGPGMVSSLDAALGPGPGKSLNSSRAKGPGPGVLASAAPVFGPGPGAATTGTQGYGPGPGTAIELSSKYGPGPGRQATNAEVLGPGPGNNQILRTILYYPLTTGPGPGAQLLDAVIFAFAEIDGPGPGKDGDFAMSYDDALSIGPGPGMRVNTNVSALIANNQGPGPGNNNEIKIQLVNTASIGPGPGNNEIKIQLVNTASIMGPGPGRYETSLAIADEIGLDGPGPGETSLAIADEIGLDNDGPGPGIAGRFKESPAPIILAGPGPGKVIVAIGGWGAEGFSGPGPGTASIMASAGITTASIGPGPGDTPAKVVIKANKLKD
2. **CotE →SlpA →FliC**
MTPQNITDLCAEYHNTQIHTLNDKIFSYTESLAGKREMAIITFKNGATFQVEVPGSQHIDSQKKAIERMKDTLRIAYLTEAKVEKLCVWNNKTPHAIAAISMANEAAAKNSSHLAWMYGPGPGSCMDWYLCFGPGPGLLDAVIFAFGPGPGYINKNGYEYGPGPGVLASAAPVFGPGPGAATTGTQGYGPGPGTAIELSSKYGPGPGRQATNAEVLGPGPGLSSGVRIKRGPGPGTTASIGSMKGPGPGMVSSLDAALGPGPGKSLNSSRAKGPGPGNNQILRTILYYPLTTGPGPGAQLLDAVIFAFAEIDGPGPGKDGDFAMSYDDALSIGPGPGRYETSLAIADEIGLDGPGPGETSLAIADEIGLDNDGPGPGIAGRFKESPAPIILAGPGPGMRVNTNVSALIANNQGPGPGNNNEIKIQLVNTASIGPGPGNNEIKIQLVNTASIMGPGPGKVIVAIGGWGAEGFSGPGPGDTPAKVVIKANKLKDGPGPGTASIMASAGITTASI
3. **FliC →SlpA →CotE**
MTPQNITDLCAEYHNTQIHTLNDKIFSYTESLAGKREMAIITFKNGATFQVEVPGSQHIDSQKKAIERMKDTLRIAYLTEAKVEKLCVWNNKTPHAIAAISMANEAAAKLSSGVRIKRGPGPGTTASIGSMKGPGPGMVSSLDAALGPGPGKSLNSSRAKGPGPGVLASAAPVFGPGPGAATTGTQGYGPGPGTAIELSSKYGPGPGRQATNAEVLGPGPGNSSHLAWMYGPGPGSCMDWYLCFGPGPGLLDAVIFAFGPGPGYINKNGYEYGPGPGMRVNTNVSALIANNQGPGPGNNNEIKIQLVNTASIGPGPGNNEIKIQLVNTASIMGPGPGRYETSLAIADEIGLDGPGPGETSLAIADEIGLDNDGPGPGIAGRFKESPAPIILAGPGPGNNQILRTILYYPLTTGPGPGAQLLDAVIFAFAEIDGPGPGKDGDFAMSYDDALSIGPGPGTASIMASAGITTASIGPGPGDTPAKVVIKANKLKDGPGPGKVIVAIGGWGAEGFS
4. **FliC →CotE →SlpA** ***(selected for further studies)***
MTPQNITDLCAEYHNTQIHTLNDKIFSYTESLAGKREMAIITFKNGATFQVEVPGSQHIDSQKKAIERMKDTLRIAYLTEAKVEKLCVWNNKTPHAIAAISMANEAAAKLSSGVRIKRGPGPGTTASIGSMKGPGPGMVSSLDAALGPGPGKSLNSSRAKGPGPGNSSHLAWMYGPGPGSCMDWYLCFGPGPGLLDAVIFAFGPGPGYINKNGYEYGPGPGVLASAAPVFGPGPGAAT

TGTQGYGPGPGTAIELSSKYGPGPGRQATNAEVLGPGPGMRVNTNVSALIANNQGPGPG NNNEIKIQLVNTASIGPGPGNNEIKIQLVNTASIMGPGPGNNQILRTILYYPLTTGPGPGAQ LLDAVIFAFAEIDGPGPGKDGDFAMSYDDALSIGPGPGRYETSLAIADEIGLDGPGPGETS LAIADEIGLDNDGPGPGIAGRFKESPAPIILAGPGPGTASIMASAGITTASIGPGPGKVIVAI GGWGAEGFSGPGPGDTPAKVVIKANKLKD

5. **SlpA →FliC →CotE**
MTPQNITDLCAEYHNTQIHTLNDKIFSYTESLAGKREMAIITFKNGATFQVEVPGSQHIDS QKKAIERMKDTLRIAYLTEAKVEKLCVWNNKTPHAIAAISMANEAAAKVLASAAPVFGP GPGAATTGTQGYGPGPGTAIELSSKYGPGPGRQATNAEVLGPGPGLSSGVRIKRGPGPGT TASIGSMKGPGPGMVSSLDAALGPGPGKSLNSSRAKGPGPGNSSHLAWMYGPGPGSCM DWYLCFGPGPGLLDAVIFAFGPGPGYINKNGYEYGPGPGRYETSLAIADEIGLDGPGPGE TSLAIADEIGLDNDGPGPGIAGRFKESPAPIILAGPGPGMRVNTNVSALIANNQGPGPGNN NEIKIQLVNTASIGPGPGNNEIKIQLVNTASIMGPGPGNNQILRTILYYPLTTGPGPGAQLL DAVIFAFAEIDGPGPGKDGDFAMSYDDALSIGPGPGDTPAKVVIKANKLKDGPGPGTASI MASAGITTASIGPGPGKVIVAIGGWGAEGFS

6. **SlpA →CotE →FliC**
MTPQNITDLCAEYHNTQIHTLNDKIFSYTESLAGKREMAIITFKNGATFQVEVPGSQHIDS QKKAIERMKDTLRIAYLTEAKVEKLCVWNNKTPHAIAAISMANEAAAKVLASAAPVFGP GPGAATTGTQGYGPGPGTAIELSSKYGPGPGRQATNAEVLGPGPGNSSHLAWMYGPGP GSCMDWYLCFGPGPGLLDAVIFAFGPGPGYINKNGYEYGPGPGLSSGVRIKRGPGPGTTA SIGSMKGPGPGMVSSLDAALGPGPGKSLNSSRAKGPGPGRYETSLAIADEIGLDGPGPGE TSLAIADEIGLDNDGPGPGIAGRFKESPAPIILAGPGPGNNQILRTILYYPLTTGPGPGAQLL DAVIFAFAEIDGPGPGKDGDFAMSYDDALSIGPGPGMRVNTNVSALIANNQGPGPGNNN EIKIQLVNTASIGPGPGNNEIKIQLVNTASIMGPGPGDTPAKVVIKANKLKDGPGPGKVIV AIGGWGAEGFSGPGPGTASIMASAGITTASI

Supplementary material SM 2: Physicochemical Properties

Number of amino acids: 512

Molecular weight: 51649.46

Theoretical pI: 5.83

Amino acid composition:

Ala (A) 54 10.5%

Arg (R) 11 2.1%

Asn (N) 27 5.3%

Asp (D) 20 3.9%

Cys (C) 4 0.8%

Gln (Q) 12 2.3%

Glu (E)	20	3.9%
Gly (G)	86	16.8%
His (H)	5	1.0%
Ile (I)	40	7.8%
Leu (L)	32	6.2%
Lys (K)	25	4.9%
Met (M)	12	2.3%
Phe (F)	12	2.3%
Pro (P)	54	10.5%
Ser (S)	32	6.2%
Thr (T)	29	5.7%
Trp (W)	4	0.8%
Tyr (Y)	14	2.7%
Val (V)	19	3.7%
Pyl (O)	0	0.0%
Sec (U)	0	0.0%
(B)	0	0.0%
(Z)	0	0.0%
(X)	0	0.0%

Total number of negatively charged residues (Asp + Glu): 40

Total number of positively charged residues (Arg + Lys): 36

Atomic composition:

Carbon	C	2287
Hydrogen	H	3601
Nitrogen	N	623
Oxygen	O	707
Sulfur	S	16

Formula: $C_{2287}H_{3601}N_{623}O_{707}S_{16}$

Total number of atoms: 7234

Extinction coefficients:

Extinction coefficients are in units of M^{-1} cm^{-1}, at 280 nm measured in water.

Ext. coefficient 43110

Abs 0.1% (=1 g/l) 0.835, assuming all pairs of Cys residues form cystines

Ext. coefficient 42860

Abs 0.1% (=1 g/l) 0.830, assuming all Cys residues are reduced

Estimated half-life:

The N-terminal of the sequence considered is M (Met).

The estimated half-life is: 30 hours (mammalian reticulocytes, in vitro).

>20 hours (yeast, in vivo).

>10 hours (Escherichia coli, in vivo).

Instability index:

The instability index (II) is computed to be 22.16

This classifies the protein as stable.

Aliphatic index: 76.15

Grand average of hydropathicity (GRAVY): -0.162

Supplementary material SM 3:

Vaccine-TLR 4/MD2 PDBsum interacting molecules

Hydrogen bonds

	<----- A T O M 1 ----->						<----- A T O M 2 ----->					
	Atom no.	Atom name	Res name	Res no.	Chain		Atom no.	Atom name	Res name	Res no.	Chain	Distance
1.	274	OG	SER	29	A	<-->	15262	OE2	GLU	84	B	2.68
2.	294	NZ	LYS	31	A	<-->	15254	O	VAL	83	B	2.86
3.	294	NZ	LYS	31	A	<-->	15261	OE1	GLU	84	B	2.58
4.	534	OE1	GLN	55	A	<-->	15287	N	CYS	87	B	3.22

5. 534 OE1 GLN 55 A <--> 15291 SG CYS 87 B 3.33

6. 4327 OE2 GLU 447 A <--> 16577 NE2 GLN 242 B 2.91

7. 4539 NH1 ARG 469 A <--> 18624 OE1 GLU 489 B 2.63

8. 4542 NH2 ARG 469 A <--> 18625 OE2 GLU 489 B 2.75

9. 4552 OD1 ASN 470 A <--> 17217 ND2 ASN 318 B 2.83

10. 4553 ND2 ASN 470 A <--> 17239 OE1 GLU 320 B 2.96

11. 4553 ND2 ASN 470 A <--> 17504 OH TYR 348 B 3.11

12. 4714 OG1 THR 487 A <--> 18699 O THR 499 B 2.68

13. 4742 OD1 ASN 490 A <--> 18612 N ALA 488 B 2.92

14. 4796 OE1 GLN 496 A <--> 16877 NE ARG 279 B 3.33

15. 4796 OE1 GLN 496 A <--> 16883 NH2 ARG 279 B 2.92

16. 5009 ND2 ASN 517 A <--> 16939 OG SER 285 B 3.03

17. 5038 NE2 GLN 520 A <--> 16421 O PRO 220 B 3.25

18. 13727 SD MET 1408 A <--> 18720 NZ LYS 502 B 3.03

19. 14173 NZ LYS 1453 A <--> 18522 O PRO 476 B 2.72

Non-bonded contacts

<----- A T O M 1 -----> <----- A T O M 2 ----->

Atom Atom Res Res Atom Atom Res Res

no. name name no. Chain no. name name no. Chain Distance

1. 55 CG1 VAL 7 A <--> 15378 NE2 HIS 95 B 3.76

2. 56 CG2 VAL 7 A <--> 15378 NE2 HIS 95 B 3.29

3. 269 O PHE 28 A <--> 15259 CG GLU 84 B 3.74

4. 264 CD2 PHE 28 A <--> 15196 OH TYR 77 B 3.64

5.	266	CE2	PHE	28	A	<-->	14865	CG	LYS	44	B	3.73
6.	272	CA	SER	29	A	<-->	15259	CG	GLU	84	B	3.65
7.	272	CA	SER	29	A	<-->	15260	CD	GLU	84	B	3.38
8.	272	CA	SER	29	A	<-->	15261	OE1	GLU	84	B	3.89
9.	272	CA	SER	29	A	<-->	15262	OE2	GLU	84	B	3.34
10.	273	CB	SER	29	A	<-->	15260	CD	GLU	84	B	3.48
11.	273	CB	SER	29	A	<-->	15261	OE1	GLU	84	B	3.65
12.	273	CB	SER	29	A	<-->	15262	OE2	GLU	84	B	3.27
13.	274	OG	SER	29	A	<-->	15260	CD	GLU	84	B	3.36
14.	274	OG	SER	29	A	<-->	15261	OE1	GLU	84	B	3.70
15.	274	OG	SER	29	A	<-->	15262	OE2	GLU	84	B	2.68
16.	291	CG	LYS	31	A	<-->	15264	O	GLU	84	B	3.63
17.	292	CD	LYS	31	A	<-->	15254	O	VAL	83	B	3.90
18.	292	CD	LYS	31	A	<-->	15257	CA	GLU	84	B	3.76
19.	292	CD	LYS	31	A	<-->	15264	O	GLU	84	B	3.32
20.	293	CE	LYS	31	A	<-->	15254	O	VAL	83	B	3.84
21.	293	CE	LYS	31	A	<-->	15261	OE1	GLU	84	B	3.55
22.	294	NZ	LYS	31	A	<-->	15253	C	VAL	83	B	3.46
23.	294	NZ	LYS	31	A	<-->	15254	O	VAL	83	B	2.86
24.	294	NZ	LYS	31	A	<-->	15255	N	GLU	84	B	3.76
25.	294	NZ	LYS	31	A	<-->	15257	CA	GLU	84	B	3.54
26.	294	NZ	LYS	31	A	<-->	15260	CD	GLU	84	B	3.73
27.	294	NZ	LYS	31	A	<-->	15261	OE1	GLU	84	B	2.58
28.	512	CB	GLU	53	A	<-->	15258	CB	GLU	84	B	3.69
29.	512	CB	GLU	53	A	<-->	15259	CG	GLU	84	B	3.75

30. 513 CG GLU 53 A <--> 15258 CB GLU 84 B 3.62

31. 532 CG GLN 55 A <--> 15264 O GLU 84 B 3.33

32. 532 CG GLN 55 A <--> 15291 SG CYS 87 B 3.71

33. 533 CD GLN 55 A <--> 15264 O GLU 84 B 3.29

34. 533 CD GLN 55 A <--> 15267 CA LYS 85 B 3.71

35. 534 OE1 GLN 55 A <--> 15264 O GLU 84 B 3.26

36. 534 OE1 GLN 55 A <--> 15267 CA LYS 85 B 3.43

37. 534 OE1 GLN 55 A <--> 15276 C LYS 85 B 3.69

38. 534 OE1 GLN 55 A <--> 15278 N LEU 86 B 3.50

39. 534 OE1 GLN 55 A <--> 15287 N CYS 87 B 3.22

40. 534 OE1 GLN 55 A <--> 15289 CA CYS 87 B 3.79

41. 534 OE1 GLN 55 A <--> 15290 CB CYS 87 B 3.39

42. 534 OE1 GLN 55 A <--> 15291 SG CYS 87 B 3.33

43. 535 NE2 GLN 55 A <--> 15267 CA LYS 85 B 3.78

44. 741 CB HIS 77 A <--> 15268 CB LYS 85 B 3.85

45. 743 ND1 HIS 77 A <--> 15270 CD LYS 85 B 3.85

46. 745 CE1 HIS 77 A <--> 15270 CD LYS 85 B 3.80

47. 746 NE2 HIS 77 A <--> 15270 CD LYS 85 B 3.87

48. 771 OG1 THR 80 A <--> 15335 OD1 ASN 91 B 3.78

49. 4069 O ARG 420 A <--> 17205 O PRO 316 B 3.46

50. 4069 O ARG 420 A <--> 17201 CB PRO 316 B 3.40

51. 4069 O ARG 420 A <--> 17202 CG PRO 316 B 3.46

52. 4072 CA ASN 421 A <--> 17205 O PRO 316 B 3.35

53. 4074 CG ASN 421 A <--> 17204 C PRO 316 B 3.69

54. 4074 CG ASN 421 A <--> 17205 O PRO 316 B 3.45

55. 4075 OD1 ASN 421 A <--> 17200 CA PRO 316 B 3.54

56. 4075 OD1 ASN 421 A <--> 17204 C PRO 316 B 3.63

57. 4075 OD1 ASN 421 A <--> 17205 O PRO 316 B 3.72

58. 4075 OD1 ASN 421 A <--> 17201 CB PRO 316 B 3.41

59. 4076 ND2 ASN 421 A <--> 17204 C PRO 316 B 3.62

60. 4076 ND2 ASN 421 A <--> 17205 O PRO 316 B 3.47

61. 4076 ND2 ASN 421 A <--> 17206 N GLY 317 B 3.87

62. 4096 CD1 ILE 423 A <--> 16575 CD GLN 242 B 3.81

63. 4096 CD1 ILE 423 A <--> 16577 NE2 GLN 242 B 3.31

64. 4096 CD1 ILE 423 A <--> 17210 O GLY 317 B 3.53

65. 4298 CB SER 444 A <--> 17537 OG1 THR 352 B 3.59

66. 4298 CB SER 444 A <--> 17539 CG2 THR 352 B 3.83

67. 4305 CA SER 445 A <--> 17214 CB ASN 318 B 3.86

68. 4305 CA SER 445 A <--> 17215 CG ASN 318 B 3.84

69. 4306 CB SER 445 A <--> 17205 O PRO 316 B 3.45

70. 4306 CB SER 445 A <--> 17214 CB ASN 318 B 3.79

71. 4306 CB SER 445 A <--> 17215 CG ASN 318 B 3.68

72. 4306 CB SER 445 A <--> 17216 OD1 ASN 318 B 3.70

73. 4307 OG SER 445 A <--> 17205 O PRO 316 B 3.50

74. 4307 OG SER 445 A <--> 17539 CG2 THR 352 B 3.69

75. 4325 CD GLU 447 A <--> 16577 NE2 GLN 242 B 3.25

76. 4326 OE1 GLU 447 A <--> 16574 CG GLN 242 B 3.19

77. 4326 OE1 GLU 447 A <--> 16575 CD GLN 242 B 3.65

78. 4326 OE1 GLU 447 A <--> 16577 NE2 GLN 242 B 3.25

79. 4327 OE2 GLU 447 A <--> 16577 NE2 GLN 242 B 2.91

80. 4335 CG2 VAL 448 A <--> 16564 CB THR 241 B 3.65

81. 4335 CG2 VAL 448 A <--> 16565 OG1 THR 241 B 3.65

82. 4536 NE ARG 469 A <--> 17511 CG PRO 349 B 3.87

83. 4538 CZ ARG 469 A <--> 17511 CG PRO 349 B 3.76

84. 4538 CZ ARG 469 A <--> 18624 OE1 GLU 489 B 3.56

85. 4538 CZ ARG 469 A <--> 18625 OE2 GLU 489 B 3.46

86. 4539 NH1 ARG 469 A <--> 18623 CD GLU 489 B 3.34

87. 4539 NH1 ARG 469 A <--> 18624 OE1 GLU 489 B 2.63

88. 4539 NH1 ARG 469 A <--> 18625 OE2 GLU 489 B 3.31

89. 4542 NH2 ARG 469 A <--> 17511 CG PRO 349 B 3.64

90. 4542 NH2 ARG 469 A <--> 18623 CD GLU 489 B 3.54

91. 4542 NH2 ARG 469 A <--> 18624 OE1 GLU 489 B 3.64

92. 4542 NH2 ARG 469 A <--> 18625 OE2 GLU 489 B 2.75

93. 4551 CG ASN 470 A <--> 17217 ND2 ASN 318 B 3.41

94. 4551 CG ASN 470 A <--> 17239 OE1 GLU 320 B 3.68

95. 4552 OD1 ASN 470 A <--> 17215 CG ASN 318 B 3.90

96. 4552 OD1 ASN 470 A <--> 17217 ND2 ASN 318 B 2.83

97. 4552 OD1 ASN 470 A <--> 17239 OE1 GLU 320 B 3.55

98. 4553 ND2 ASN 470 A <--> 17217 ND2 ASN 318 B 3.70

99. 4553 ND2 ASN 470 A <--> 17239 OE1 GLU 320 B 2.96

100. 4553 ND2 ASN 470 A <--> 17504 OH TYR 348 B 3.11

101. 4579 CB PHE 473 A <--> 16567 CG2 THR 241 B 3.82

102. 4698 CB SER 485 A <--> 18695 OG1 THR 499 B 3.67

103. 4706 CG PRO 486 A <--> 18687 OD1 ASP 498 B 3.23

104. 4707 CD PRO 486 A <--> 18695 OG1 THR 499 B 3.55

105. 4713 CB THR 487 A <--> 18699 O THR 499 B 3.67

106. 4714 OG1 THR 487 A <--> 18693 CA THR 499 B 3.80

107. 4714 OG1 THR 487 A <--> 18698 C THR 499 B 3.44

108. 4714 OG1 THR 487 A <--> 18699 O THR 499 B 2.68

109. 4714 OG1 THR 487 A <--> 18694 CB THR 499 B 3.08

110. 4714 OG1 THR 487 A <--> 18697 CG2 THR 499 B 3.75

111. 4716 CG2 THR 487 A <--> 18699 O THR 499 B 3.51

112. 4716 CG2 THR 487 A <--> 18710 CB ALA 501 B 3.80

113. 4741 CG ASN 490 A <--> 18612 N ALA 488 B 3.72

114. 4742 OD1 ASN 490 A <--> 18609 CA GLY 487 B 3.80

115. 4742 OD1 ASN 490 A <--> 18610 C GLY 487 B 3.82

116. 4742 OD1 ASN 490 A <--> 18612 N ALA 488 B 2.92

117. 4742 OD1 ASN 490 A <--> 18614 CA ALA 488 B 3.75

118. 4743 ND2 ASN 490 A <--> 18612 N ALA 488 B 3.84

119. 4743 ND2 ASN 490 A <--> 18616 C ALA 488 B 3.31

120. 4743 ND2 ASN 490 A <--> 18617 O ALA 488 B 3.52

121. 4743 ND2 ASN 490 A <--> 18618 N GLU 489 B 3.32

122. 4743 ND2 ASN 490 A <--> 18620 CA GLU 489 B 3.57

123. 4743 ND2 ASN 490 A <--> 18622 CG GLU 489 B 3.70

124. 4768 CB SER 493 A <--> 16922 ND2 ASN 283 B 3.88

125. 4795 CD GLN 496 A <--> 16883 NH2 ARG 279 B 3.55

126. 4796 OE1 GLN 496 A <--> 16877 NE ARG 279 B 3.33

127. 4796 OE1 GLN 496 A <--> 16879 CZ ARG 279 B 3.52

128. 4796 OE1 GLN 496 A <--> 16883 NH2 ARG 279 B 2.92

129. 4797 NE2 GLN 496 A <--> 16883 NH2 ARG 279 B 3.57

130. 4948 CE2 PHE 511 A <--> 18687 OD1 ASP 498 B 3.83

131. 4985 O LYS 514 A <--> 18603 CZ3 TRP 486 B 3.11

132. 4985 O LYS 514 A <--> 18604 CH2 TRP 486 B 3.74

133. 5006 CB ASN 517 A <--> 16921 OD1 ASN 283 B 3.09

134. 5007 CG ASN 517 A <--> 16921 OD1 ASN 283 B 3.87

135. 5009 ND2 ASN 517 A <--> 16921 OD1 ASN 283 B 3.58

136. 5009 ND2 ASN 517 A <--> 16934 O VAL 284 B 3.86

137. 5009 ND2 ASN 517 A <--> 16938 CB SER 285 B 3.66

138. 5009 ND2 ASN 517 A <--> 16939 OG SER 285 B 3.03

139. 5036 CD GLN 520 A <--> 16416 CA PRO 220 B 3.79

140. 5036 CD GLN 520 A <--> 16420 C PRO 220 B 3.89

141. 5036 CD GLN 520 A <--> 16421 O PRO 220 B 3.56

142. 5037 OE1 GLN 520 A <--> 16414 O GLY 219 B 3.47

143. 5037 OE1 GLN 520 A <--> 16416 CA PRO 220 B 3.31

144. 5037 OE1 GLN 520 A <--> 16420 C PRO 220 B 3.34

145. 5037 OE1 GLN 520 A <--> 16421 O PRO 220 B 3.31

146. 5038 NE2 GLN 520 A <--> 16421 O PRO 220 B 3.25

147. 5226 O GLN 538 A <--> 16751 NE ARG 264 B 3.72

148. 5226 O GLN 538 A <--> 16753 CZ ARG 264 B 3.64

149. 5226 O GLN 538 A <--> 16754 NH1 ARG 264 B 3.79

150. 5221 OE1 GLN 538 A <--> 16737 CD PRO 262 B 3.53

151. 5229 CA HIS 539 A <--> 16754 NH1 ARG 264 B 3.54

152. 5231 CG HIS 539 A <--> 16757 NH2 ARG 264 B 3.79

153. 5232 ND1 HIS 539 A <--> 16754 NH1 ARG 264 B 3.55

154. 5232 ND1 HIS 539 A <--> 16757 NH2 ARG 264 B 3.35

155. 5234 CE1 HIS 539 A <--> 16757 NH2 ARG 264 B 3.36

156. 5235 NE2 HIS 539 A <--> 16757 NH2 ARG 264 B 3.80

157. 5250 O PHE 540 A <--> 16727 O PRO 260 B 3.68

158. 5242 CB PHE 540 A <--> 16724 CG PRO 260 B 3.86

159. 5265 O SER 542 A <--> 16045 CG PRO 176 B 3.26

160. 5265 O SER 542 A <--> 16046 CD PRO 176 B 3.70

161. 5261 CB SER 542 A <--> 16684 CB SER 256 B 3.48

162. 5261 CB SER 542 A <--> 16685 OG SER 256 B 3.87

163. 5262 OG SER 542 A <--> 16777 CB ALA 266 B 3.25

164. 12210 CG GLN 1256 A <--> 16508 O PRO 232 B 3.86

165. 12213 NE2 GLN 1256 A <--> 16514 N PRO 234 B 3.69

166. 12213 NE2 GLN 1256 A <--> 16515 CA PRO 234 B 3.81

167. 12213 NE2 GLN 1256 A <--> 16516 CB PRO 234 B 3.49

168. 12213 NE2 GLN 1256 A <--> 16517 CG PRO 234 B 3.33

169. 12213 NE2 GLN 1256 A <--> 16518 CD PRO 234 B 3.89

170. 12443 O CYS 1278 A <--> 16504 CB PRO 232 B 3.84

171. 12462 O ARG 1279 A <--> 16503 CA PRO 232 B 3.43

172. 12462 O ARG 1279 A <--> 16507 C PRO 232 B 3.67

173. 12462 O ARG 1279 A <--> 16509 N GLY 233 B 3.78

174. 12877 C GLY 1323 A <--> 16517 CG PRO 234 B 3.80

175. 12878 O GLY 1323 A <--> 16517 CG PRO 234 B 3.75

176. 12879 N SER 1324 A <--> 16517 CG PRO 234 B 3.73

177. 12881 CA SER 1324 A <--> 16517 CG PRO 234 B 3.61

178. 12882 CB SER 1324 A <--> 16520 O PRO 234 B 3.62

179. 12883 OG SER 1324 A <--> 16520 O PRO 234 B 3.60

180. 12883 OG SER 1324   A   <--> 16516 CB PRO 234   B   3.50

181. 12883 OG SER 1324   A   <--> 16517 CG PRO 234   B   3.67

182. 13712 O   ASN 1406   A   <--> 18719 CE LYS 502   B   3.27

183. 13712 O   ASN 1406   A   <--> 18720 NZ LYS 502   B   3.78

184. 13717 OG1 THR 1407   A   <--> 18718 CD LYS 502   B   3.69

185. 13727 SD MET 1408   A   <--> 18719 CE LYS 502   B   3.78

186. 13727 SD MET 1408   A   <--> 18720 NZ LYS 502   B   3.03

187. 14172 CE LYS 1453   A   <--> 18522 O   PRO 476   B   3.49

188. 14173 NZ LYS 1453   A   <--> 18521 C   PRO 476   B   3.85

189. 14173 NZ LYS 1453   A   <--> 18522 O   PRO 476   B   2.72

190. 14173 NZ LYS 1453   A   <--> 18520 CD PRO 476   B   3.87

Salt bridges

<----- A T O M   1 ----->   <----- A T O M   2 ----->

Atom Atom Res Res   Atom Atom Res Res

no. name name no. Chain   no. name name no. Chain Distance

1.   294 NZ LYS   31   A   <--> 15261 OE1 GLU   84   B   2.58

2.   4539 NH1 ARG 469   A   <--> 18625 OE2 GLU 489   B   2.63

Number of salt bridges: 2

Number of hydrogen bonds: 19

Number of non-bonded contacts: 190

Supplementary material SM 4:
Vaccine TLR 5 PDBsum interacting molecules

Hydrogen bonds

	<----- A T O M 1 ----->						<----- A T O M 2 ----->					
	Atom	Atom	Res	Res			Atom	Atom	Res	Res		
	no.	name	name	no.	Chain		no.	name	name	no.	Chain	Distance
1.	36	O	PHE	4	A	<-->	14810	NZ	LYS	85	B	3.32
2.	43	OD2	ASP	5	A	<-->	14810	NZ	LYS	85	B	2.65
3.	451	O	PRO	45	A	<-->	15721	N	GLY	193	B	3.09
4.	6030	O	LEU	622	A	<-->	14009	NE2	GLN	4	B	2.76
5.	6127	NH1	ARG	632	A	<-->	14198	OD1	ASP	23	B	2.75
6.	6130	NH2	ARG	632	A	<-->	14199	OD2	ASP	23	B	2.91
7.	6245	NZ	LYS	644	A	<-->	14824	O	LEU	86	B	3.09
8.	6245	NZ	LYS	644	A	<-->	14829	SG	CYS	87	B	3.28
9.	6580	NH1	ARG	676	A	<-->	14800	OE2	GLU	84	B	2.91
10.	6583	NH2	ARG	676	A	<-->	14799	OE1	GLU	84	B	2.62
11.	6734	ND2	ASN	691	A	<-->	16022	O	VAL	229	B	2.74
12.	6764	O	ILE	693	A	<-->	16059	N	GLY	235	B	3.24
13.	6809	OD2	ASP	698	A	<-->	14896	OG1	THR	93	B	2.75
14.	6849	ND2	ASN	702	A	<-->	14891	O	LYS	92	B	3.30
15.	13096	NE2	GLN	1334	A	<-->	14551	OD1	ASP	60	B	2.81
16.	13603	OD1	ASP	1389	A	<-->	14138	NE2	GLN	17	B	2.84
17.	13614	O	SER	1390	A	<-->	14117	ND2	ASN	15	B	3.03
18.	13761	O	LEU	1407	A	<-->	14117	ND2	ASN	15	B	3.17

Non-bonded contacts

	<----- A T O M 1 ----->						<----- A T O M 2 ----->					
	Atom no.	Atom name	Res name	Res no.	Chain		Atom no.	Atom name	Res name	Res no.	Chain	Distance
1.	36	O	PHE	4	A	<-->	14810	NZ	LYS	85	B	3.32
2.	40	CB	ASP	5	A	<-->	14810	NZ	LYS	85	B	3.44
3.	41	CG	ASP	5	A	<-->	14810	NZ	LYS	85	B	3.42
4.	43	OD2	ASP	5	A	<-->	14808	CD	LYS	85	B	3.71
5.	43	OD2	ASP	5	A	<-->	14809	CE	LYS	85	B	3.48
6.	43	OD2	ASP	5	A	<-->	14810	NZ	LYS	85	B	2.65
7.	248	CG2	THR	25	A	<-->	14822	CD2	LEU	86	B	3.83
8.	451	O	PRO	45	A	<-->	15711	CA	GLY	191	B	3.63
9.	451	O	PRO	45	A	<-->	15712	C	GLY	191	B	3.76
10.	451	O	PRO	45	A	<-->	15714	N	PRO	192	B	3.86
11.	451	O	PRO	45	A	<-->	15721	N	GLY	193	B	3.09
12.	451	O	PRO	45	A	<-->	15723	CA	GLY	193	B	3.39
13.	452	N	PHE	46	A	<-->	15708	O	PRO	190	B	3.89
14.	454	CA	PHE	46	A	<-->	15708	O	PRO	190	B	3.44
15.	454	CA	PHE	46	A	<-->	15711	CA	GLY	191	B	3.31
16.	455	CB	PHE	46	A	<-->	15707	C	PRO	190	B	3.65
17.	455	CB	PHE	46	A	<-->	15708	O	PRO	190	B	3.20
18.	455	CB	PHE	46	A	<-->	15711	CA	GLY	191	B	3.81
19.	480	OE2	GLU	48	A	<-->	15721	N	GLY	193	B	3.49
20.	480	OE2	GLU	48	A	<-->	15723	CA	GLY	193	B	3.16
21.	487	CG	GLN	49	A	<-->	14852	CZ2	TRP	89	B	3.55

22. 708 ND2 ASN 71 A <--> 15723 CA GLY 193 B 3.47

23. 5955 C CYS 614 A <--> 14106 CE1 HIS 14 B 3.62

24. 5956 O CYS 614 A <--> 14106 CE1 HIS 14 B 2.89

25. 5956 O CYS 614 A <--> 14107 NE2 HIS 14 B 3.47

26. 5980 CG2 THR 617 A <--> 14107 NE2 HIS 14 B 3.72

27. 5983 N LEU 618 A <--> 14107 NE2 HIS 14 B 3.46

28. 5985 CA LEU 618 A <--> 14107 NE2 HIS 14 B 3.58

29. 5986 CB LEU 618 A <--> 14107 NE2 HIS 14 B 3.61

30. 5989 CD2 LEU 618 A <--> 14105 CD2 HIS 14 B 3.61

31. 6021 O PHE 621 A <--> 14040 CG2 THR 7 B 3.52

32. 6016 CD2 PHE 621 A <--> 14151 O ILE 18 B 3.53

33. 6018 CE2 PHE 621 A <--> 14151 O ILE 18 B 3.45

34. 6029 C LEU 622 A <--> 14007 CD GLN 4 B 3.85

35. 6029 C LEU 622 A <--> 14008 OE1 GLN 4 B 3.27

36. 6029 C LEU 622 A <--> 14009 NE2 GLN 4 B 3.59

37. 6030 O LEU 622 A <--> 14007 CD GLN 4 B 3.32

38. 6030 O LEU 622 A <--> 14008 OE1 GLN 4 B 3.09

39. 6030 O LEU 622 A <--> 14009 NE2 GLN 4 B 2.76

40. 6026 CG LEU 622 A <--> 14008 OE1 GLN 4 B 3.89

41. 6027 CD1 LEU 622 A <--> 14008 OE1 GLN 4 B 3.33

42. 6028 CD2 LEU 622 A <--> 14008 OE1 GLN 4 B 3.48

43. 6031 N MET 623 A <--> 14008 OE1 GLN 4 B 3.42

44. 6031 N MET 623 A <--> 14009 NE2 GLN 4 B 3.84

45. 6033 CA MET 623 A <--> 14007 CD GLN 4 B 3.58

46. 6033 CA MET 623 A <--> 14008 OE1 GLN 4 B 3.36

47. 6033 CA MET 623 A <--> 14009 NE2 GLN 4 B 3.29
48. 6038 C MET 623 A <--> 14009 NE2 GLN 4 B 3.78
49. 6035 CG MET 623 A <--> 14008 OE1 GLN 4 B 3.49
50. 6040 N THR 624 A <--> 14009 NE2 GLN 4 B 3.83
51. 6049 N ILE 625 A <--> 14009 NE2 GLN 4 B 3.86
52. 6056 C ILE 625 A <--> 14009 NE2 GLN 4 B 3.80
53. 6057 O ILE 625 A <--> 14002 N GLN 4 B 3.90
54. 6053 CG1 ILE 625 A <--> 14009 NE2 GLN 4 B 3.87
55. 6053 CG1 ILE 625 A <--> 14038 OG1 THR 7 B 3.62
56. 6054 CG2 ILE 625 A <--> 14168 OG1 THR 20 B 3.81
57. 6054 CG2 ILE 625 A <--> 14170 CG2 THR 20 B 3.90
58. 6054 CG2 ILE 625 A <--> 14219 CG1 ILE 25 B 3.88
59. 6055 CD1 ILE 625 A <--> 14000 C PRO 3 B 3.85
60. 6055 CD1 ILE 625 A <--> 14001 O PRO 3 B 3.61
61. 6055 CD1 ILE 625 A <--> 13997 CB PRO 3 B 3.77
62. 6055 CD1 ILE 625 A <--> 14038 OG1 THR 7 B 3.14
63. 6055 CD1 ILE 625 A <--> 14219 CG1 ILE 25 B 3.60
64. 6055 CD1 ILE 625 A <--> 14220 CG2 ILE 25 B 3.66
65. 6058 N LEU 626 A <--> 14009 NE2 GLN 4 B 3.66
66. 6061 CB LEU 626 A <--> 14006 CG GLN 4 B 3.86
67. 6061 CB LEU 626 A <--> 14009 NE2 GLN 4 B 3.53
68. 6063 CD1 LEU 626 A <--> 14005 CB GLN 4 B 3.65
69. 6063 CD1 LEU 626 A <--> 14006 CG GLN 4 B 3.72
70. 6088 OG1 THR 629 A <--> 13998 CG PRO 3 B 3.84
71. 6090 CG2 THR 629 A <--> 13998 CG PRO 3 B 3.74

72. 6100 NZ LYS 630 A <--> 13992 CG2 THR 2 B 3.43

73. 6126 CZ ARG 632 A <--> 14198 OD1 ASP 23 B 3.69

74. 6126 CZ ARG 632 A <--> 14199 OD2 ASP 23 B 3.41

75. 6127 NH1 ARG 632 A <--> 14197 CG ASP 23 B 3.21

76. 6127 NH1 ARG 632 A <--> 14198 OD1 ASP 23 B 2.75

77. 6127 NH1 ARG 632 A <--> 14199 OD2 ASP 23 B 3.11

78. 6127 NH1 ARG 632 A <--> 14405 CE LYS 44 B 3.75

79. 6127 NH1 ARG 632 A <--> 14406 NZ LYS 44 B 3.90

80. 6130 NH2 ARG 632 A <--> 14197 CG ASP 23 B 3.74

81. 6130 NH2 ARG 632 A <--> 14198 OD1 ASP 23 B 3.77

82. 6130 NH2 ARG 632 A <--> 14199 OD2 ASP 23 B 2.91

83. 6130 NH2 ARG 632 A <--> 14406 NZ LYS 44 B 3.90

84. 6211 OD1 ASP 641 A <--> 14915 CE1 HIS 95 B 3.74

85. 6222 C MET 642 A <--> 14904 CG PRO 94 B 3.74

86. 6223 O MET 642 A <--> 14904 CG PRO 94 B 3.26

87. 6223 O MET 642 A <--> 14914 CD2 HIS 95 B 3.55

88. 6223 O MET 642 A <--> 14916 NE2 HIS 95 B 3.15

89. 6220 SD MET 642 A <--> 14938 CB ALA 98 B 3.68

90. 6221 CE MET 642 A <--> 14913 ND1 HIS 95 B 3.51

91. 6226 CA TYR 643 A <--> 14904 CG PRO 94 B 3.64

92. 6238 N LYS 644 A <--> 14904 CG PRO 94 B 3.84

93. 6243 CD LYS 644 A <--> 14829 SG CYS 87 B 3.69

94. 6244 CE LYS 644 A <--> 14832 O CYS 87 B 3.26

95. 6244 CE LYS 644 A <--> 14829 SG CYS 87 B 3.76

96. 6245 NZ LYS 644 A <--> 14824 O LEU 86 B 3.09

97. 6245 NZ LYS 644 A <--> 14832 O CYS 87 B 3.05
98. 6245 NZ LYS 644 A <--> 14829 SG CYS 87 B 3.28
99. 6259 CE2 TYR 645 A <--> 14873 OD1 ASN 91 B 3.90
100. 6579 CZ ARG 676 A <--> 14799 OE1 GLU 84 B 3.56
101. 6579 CZ ARG 676 A <--> 14800 OE2 GLU 84 B 3.63
102. 6580 NH1 ARG 676 A <--> 14798 CD GLU 84 B 3.60
103. 6580 NH1 ARG 676 A <--> 14799 OE1 GLU 84 B 3.65
104. 6580 NH1 ARG 676 A <--> 14800 OE2 GLU 84 B 2.91
105. 6583 NH2 ARG 676 A <--> 14798 CD GLU 84 B 3.31
106. 6583 NH2 ARG 676 A <--> 14799 OE1 GLU 84 B 2.62
107. 6583 NH2 ARG 676 A <--> 14800 OE2 GLU 84 B 3.45
108. 6679 CA ASP 685 A <--> 14874 ND2 ASN 91 B 3.83
109. 6684 C ASP 685 A <--> 14874 ND2 ASN 91 B 3.70
110. 6680 CB ASP 685 A <--> 14874 ND2 ASN 91 B 3.44
111. 6686 N PHE 686 A <--> 14874 ND2 ASN 91 B 3.72
112. 6697 O PHE 686 A <--> 14866 C ASN 90 B 3.83
113. 6697 O PHE 686 A <--> 14867 O ASN 90 B 3.40
114. 6697 O PHE 686 A <--> 14871 CB ASN 91 B 3.74
115. 6700 CA VAL 687 A <--> 14867 O ASN 90 B 3.87
116. 6700 CA VAL 687 A <--> 14862 OD1 ASN 90 B 3.79
117. 6701 CB VAL 687 A <--> 14862 OD1 ASN 90 B 3.86
118. 6702 CG1 VAL 687 A <--> 14862 OD1 ASN 90 B 3.14
119. 6703 CG2 VAL 687 A <--> 14867 O ASN 90 B 3.57
120. 6703 CG2 VAL 687 A <--> 14878 O ASN 91 B 3.55
121. 6709 CG PRO 688 A <--> 14863 ND2 ASN 90 B 3.49

122. 6710 CD PRO 688 A <--> 14861 CG ASN 90 B 3.61

123. 6710 CD PRO 688 A <--> 14862 OD1 ASN 90 B 3.65

124. 6710 CD PRO 688 A <--> 14863 ND2 ASN 90 B 3.29

125. 6713 N GLY 689 A <--> 14863 ND2 ASN 90 B 3.88

126. 6723 CD GLU 690 A <--> 16080 OG1 THR 238 B 3.54

127. 6724 OE1 GLU 690 A <--> 16080 OG1 THR 238 B 3.66

128. 6724 OE1 GLU 690 A <--> 16082 CG2 THR 238 B 3.77

129. 6725 OE2 GLU 690 A <--> 16080 OG1 THR 238 B 3.28

130. 6725 OE2 GLU 690 A <--> 16082 CG2 THR 238 B 3.58

131. 6732 CG ASN 691 A <--> 16022 O VAL 229 B 3.54

132. 6732 CG ASN 691 A <--> 16028 CD1 PHE 230 B 3.74

133. 6733 OD1 ASN 691 A <--> 16022 O VAL 229 B 3.52

134. 6733 OD1 ASN 691 A <--> 16025 CA PHE 230 B 3.36

135. 6733 OD1 ASN 691 A <--> 16027 CG PHE 230 B 3.47

136. 6733 OD1 ASN 691 A <--> 16028 CD1 PHE 230 B 3.15

137. 6733 OD1 ASN 691 A <--> 16030 CE1 PHE 230 B 3.58

138. 6733 OD1 ASN 691 A <--> 16035 N GLY 231 B 3.53

139. 6733 OD1 ASN 691 A <--> 16067 CB ALA 236 B 3.69

140. 6734 ND2 ASN 691 A <--> 16022 O VAL 229 B 2.74

141. 6734 ND2 ASN 691 A <--> 16069 O ALA 236 B 3.80

142. 6734 ND2 ASN 691 A <--> 16082 CG2 THR 238 B 3.86

143. 6739 N ARG 692 A <--> 16030 CE1 PHE 230 B 3.50

144. 6739 N ARG 692 A <--> 16032 CZ PHE 230 B 3.85

145. 6742 CB ARG 692 A <--> 16032 CZ PHE 230 B 3.79

146. 6763 C ILE 693 A <--> 16059 N GLY 235 B 3.78

147. 6764 O ILE 693 A <--> 16053 CA PRO 234 B 3.06

148. 6764 O ILE 693 A <--> 16057 C PRO 234 B 3.54

149. 6764 O ILE 693 A <--> 16054 CB PRO 234 B 3.87

150. 6764 O ILE 693 A <--> 16059 N GLY 235 B 3.24

151. 6759 CB ILE 693 A <--> 16051 O GLY 233 B 3.74

152. 6761 CG2 ILE 693 A <--> 16050 C GLY 233 B 3.71

153. 6761 CG2 ILE 693 A <--> 16051 O GLY 233 B 3.70

154. 6761 CG2 ILE 693 A <--> 16052 N PRO 234 B 3.66

155. 6761 CG2 ILE 693 A <--> 16053 CA PRO 234 B 3.68

156. 6762 CD1 ILE 693 A <--> 16039 O GLY 231 B 3.56

157. 6762 CD1 ILE 693 A <--> 16041 CA PRO 232 B 3.52

158. 6762 CD1 ILE 693 A <--> 16045 C PRO 232 B 3.68

159. 6762 CD1 ILE 693 A <--> 16046 O PRO 232 B 3.82

160. 6765 N ALA 694 A <--> 16059 N GLY 235 B 3.87

161. 6767 CA ALA 694 A <--> 16059 N GLY 235 B 3.38

162. 6767 CA ALA 694 A <--> 16061 CA GLY 235 B 3.57

163. 6785 CB ILE 696 A <--> 16054 CB PRO 234 B 3.34

164. 6787 CG2 ILE 696 A <--> 16054 CB PRO 234 B 3.62

165. 6788 CD1 ILE 696 A <--> 16054 CB PRO 234 B 3.69

166. 6788 CD1 ILE 696 A <--> 16055 CG PRO 234 B 3.71

167. 6794 CB GLN 697 A <--> 16057 C PRO 234 B 3.70

168. 6794 CB GLN 697 A <--> 16058 O PRO 234 B 3.29

169. 6794 CB GLN 697 A <--> 16059 N GLY 235 B 3.72

170. 6794 CB GLN 697 A <--> 16061 CA GLY 235 B 3.27

171. 6795 CG GLN 697 A <--> 16058 O PRO 234 B 3.79

172. 6795 CG GLN 697 A <--> 16061 CA GLY 235 B 3.84

173. 6796 CD GLN 697 A <--> 16061 CA GLY 235 B 3.63

174. 6797 OE1 GLN 697 A <--> 16061 CA GLY 235 B 3.51

175. 6807 CG ASP 698 A <--> 14896 OG1 THR 93 B 3.44

176. 6808 OD1 ASP 698 A <--> 14896 OG1 THR 93 B 3.44

177. 6809 OD2 ASP 698 A <--> 14892 N THR 93 B 3.70

178. 6809 OD2 ASP 698 A <--> 14895 CB THR 93 B 3.67

179. 6809 OD2 ASP 698 A <--> 14896 OG1 THR 93 B 2.75

180. 6809 OD2 ASP 698 A <--> 14898 CG2 THR 93 B 3.74

181. 6842 O TRP 701 A <--> 14898 CG2 THR 93 B 3.76

182. 6834 NE1 TRP 701 A <--> 14916 NE2 HIS 95 B 3.60

183. 6836 CE2 TRP 701 A <--> 14914 CD2 HIS 95 B 3.76

184. 6836 CE2 TRP 701 A <--> 14916 NE2 HIS 95 B 3.71

185. 6837 CE3 TRP 701 A <--> 14898 CG2 THR 93 B 3.86

186. 6838 CZ2 TRP 701 A <--> 14912 CG HIS 95 B 3.71

187. 6838 CZ2 TRP 701 A <--> 14913 ND1 HIS 95 B 3.77

188. 6838 CZ2 TRP 701 A <--> 14914 CD2 HIS 95 B 3.81

189. 6838 CZ2 TRP 701 A <--> 14915 CE1 HIS 95 B 3.86

190. 6838 CZ2 TRP 701 A <--> 14916 NE2 HIS 95 B 3.90

191. 6847 CG ASN 702 A <--> 14905 CD PRO 94 B 3.64

192. 6848 OD1 ASN 702 A <--> 14904 CG PRO 94 B 3.77

193. 6848 OD1 ASN 702 A <--> 14905 CD PRO 94 B 3.03

194. 6849 ND2 ASN 702 A <--> 14873 OD1 ASN 91 B 3.88

195. 6849 ND2 ASN 702 A <--> 14890 C LYS 92 B 3.84

196. 6849 ND2 ASN 702 A <--> 14891 O LYS 92 B 3.30

197. 6849 ND2 ASN 702 A <--> 14894 CA THR 93 B 3.82

198. 7055 O LEU 721 A <--> 16055 CG PRO 234 B 3.55

199. 7055 O LEU 721 A <--> 16056 CD PRO 234 B 3.58

200. 7075 CB PHE 724 A <--> 16046 O PRO 232 B 3.58

201. 7076 CG PHE 724 A <--> 16046 O PRO 232 B 3.41

202. 7077 CD1 PHE 724 A <--> 16045 C PRO 232 B 3.68

203. 7077 CD1 PHE 724 A <--> 16046 O PRO 232 B 3.53

204. 7077 CD1 PHE 724 A <--> 16047 N GLY 233 B 3.68

205. 7077 CD1 PHE 724 A <--> 16049 CA GLY 233 B 3.55

206. 7081 CZ PHE 724 A <--> 16042 CB PRO 232 B 3.89

207. 7081 CZ PHE 724 A <--> 16043 CG PRO 232 B 3.70

208. 7087 CB SER 725 A <--> 16055 CG PRO 234 B 3.75

209. 7087 CB SER 725 A <--> 16056 CD PRO 234 B 3.74

210. 7088 OG SER 725 A <--> 16055 CG PRO 234 B 3.04

211. 7088 OG SER 725 A <--> 16056 CD PRO 234 B 3.33

212. 13094 CD GLN 1334 A <--> 14551 OD1 ASP 60 B 3.55

213. 13095 OE1 GLN 1334 A <--> 14551 OD1 ASP 60 B 3.48

214. 13095 OE1 GLN 1334 A <--> 14582 NZ LYS 63 B 3.45

215. 13096 NE2 GLN 1334 A <--> 14550 CG ASP 60 B 3.69

216. 13096 NE2 GLN 1334 A <--> 14551 OD1 ASP 60 B 2.81

217. 13115 CD1 LEU 1336 A <--> 14533 NE2 HIS 58 B 3.46

218. 13590 O TYR 1387 A <--> 14272 CD GLU 30 B 3.79

219. 13590 O TYR 1387 A <--> 14273 OE1 GLU 30 B 3.65

220. 13590 O TYR 1387 A <--> 14274 OE2 GLU 30 B 3.19

221. 13583 CD2 TYR 1387 A <--> 14540 CB ILE 59 B 3.64

222. 13583 CD2 TYR 1387 A <--> 14541 CG1 ILE 59 B 3.87
223. 13585 CE2 TYR 1387 A <--> 14532 CE1 HIS 58 B 3.84
224. 13585 CE2 TYR 1387 A <--> 14540 CB ILE 59 B 3.50
225. 13585 CE2 TYR 1387 A <--> 14542 CG2 ILE 59 B 3.76
226. 13587 OH TYR 1387 A <--> 14532 CE1 HIS 58 B 3.46
227. 13598 N ASP 1389 A <--> 14291 CD2 LEU 32 B 3.63
228. 13606 O ASP 1389 A <--> 14121 O ASN 15 B 3.64
229. 13606 O ASP 1389 A <--> 14114 CB ASN 15 B 3.59
230. 13602 CG ASP 1389 A <--> 14135 CG GLN 17 B 3.66
231. 13602 CG ASP 1389 A <--> 14138 NE2 GLN 17 B 3.28
232. 13603 OD1 ASP 1389 A <--> 14136 CD GLN 17 B 3.82
233. 13603 OD1 ASP 1389 A <--> 14138 NE2 GLN 17 B 2.84
234. 13604 OD2 ASP 1389 A <--> 14120 C ASN 15 B 3.46
235. 13604 OD2 ASP 1389 A <--> 14121 O ASN 15 B 3.45
236. 13604 OD2 ASP 1389 A <--> 14122 N THR 16 B 3.45
237. 13604 OD2 ASP 1389 A <--> 14124 CA THR 16 B 3.44
238. 13604 OD2 ASP 1389 A <--> 14129 C THR 16 B 3.26
239. 13604 OD2 ASP 1389 A <--> 14130 O THR 16 B 3.51
240. 13604 OD2 ASP 1389 A <--> 14131 N GLN 17 B 3.64
241. 13604 OD2 ASP 1389 A <--> 14135 CG GLN 17 B 3.40
242. 13604 OD2 ASP 1389 A <--> 14136 CD GLN 17 B 3.73
243. 13604 OD2 ASP 1389 A <--> 14138 NE2 GLN 17 B 3.19
244. 13607 N SER 1390 A <--> 14291 CD2 LEU 32 B 3.70
245. 13613 C SER 1390 A <--> 14117 ND2 ASN 15 B 3.78
246. 13614 O SER 1390 A <--> 14117 ND2 ASN 15 B 3.03

247. 13610 CB SER 1390 A <--> 14290 CD1 LEU 32 B 3.60

248. 13611 OG SER 1390 A <--> 14290 CD1 LEU 32 B 3.75

249. 13611 OG SER 1390 A <--> 14291 CD2 LEU 32 B 3.54

250. 13761 O LEU 1407 A <--> 14117 ND2 ASN 15 B 3.17

251. 13790 O VAL 1410 A <--> 14102 CB HIS 14 B 3.57

252. 13790 O VAL 1410 A <--> 14104 ND1 HIS 14 B 3.85

253. 13788 CG2 VAL 1410 A <--> 14116 OD1 ASN 15 B 3.45

254. 13791 N CYS 1411 A <--> 14116 OD1 ASN 15 B 3.78

255. 13793 CA CYS 1411 A <--> 14116 OD1 ASN 15 B 3.77

256. 13798 O CYS 1411 A <--> 14084 O GLU 12 B 3.77

257. 13794 CB CYS 1411 A <--> 14115 CG ASN 15 B 3.58

258. 13794 CB CYS 1411 A <--> 14116 OD1 ASN 15 B 3.19

259. 13795 SG CYS 1411 A <--> 14087 CA TYR 13 B 3.57

260. 13795 SG CYS 1411 A <--> 14097 C TYR 13 B 3.80

261. 13795 SG CYS 1411 A <--> 14088 CB TYR 13 B 3.69

262. 13795 SG CYS 1411 A <--> 14090 CD1 TYR 13 B 3.59

263. 13795 SG CYS 1411 A <--> 14099 N HIS 14 B 3.41

264. 13795 SG CYS 1411 A <--> 14111 N ASN 15 B 3.52

265. 13795 SG CYS 1411 A <--> 14114 CB ASN 15 B 3.87

266. 13795 SG CYS 1411 A <--> 14116 OD1 ASN 15 B 3.85

267. 13823 C THR 1414 A <--> 14084 O GLU 12 B 3.41

268. 13824 O THR 1414 A <--> 14084 O GLU 12 B 3.71

269. 13819 CB THR 1414 A <--> 14102 CB HIS 14 B 3.76

270. 13820 OG1 THR 1414 A <--> 14102 CB HIS 14 B 3.41

271. 13820 OG1 THR 1414 A <--> 14103 CG HIS 14 B 3.71

272. 13820 OG1 THR 1414 A <--> 14104 ND1 HIS 14 B 3.85

273. 13822 CG2 THR 1414 A <--> 14074 O ALA 11 B 3.74

274. 13825 N LEU 1415 A <--> 14084 O GLU 12 B 3.01

275. 13827 CA LEU 1415 A <--> 14083 C GLU 12 B 3.89

276. 13827 CA LEU 1415 A <--> 14084 O GLU 12 B 2.95

277. 13833 O LEU 1415 A <--> 14079 CG GLU 12 B 3.71

278. 13828 CB LEU 1415 A <--> 14084 O GLU 12 B 3.54

279. 13830 CD1 LEU 1415 A <--> 14084 O GLU 12 B 3.84

280. 13830 CD1 LEU 1415 A <--> 14078 CB GLU 12 B 3.53

281. 13830 CD1 LEU 1415 A <--> 14092 CE1 TYR 13 B 3.84

282. 13830 CD1 LEU 1415 A <--> 14094 CZ TYR 13 B 3.84

283. 13855 CB PHE 1418 A <--> 14079 CG GLU 12 B 3.62

284. 13857 CD1 PHE 1418 A <--> 14048 OD1 ASP 8 B 3.23

285. 13857 CD1 PHE 1418 A <--> 14072 CB ALA 11 B 3.88

286. 13858 CD2 PHE 1418 A <--> 14073 C ALA 11 B 3.59

287. 13858 CD2 PHE 1418 A <--> 14074 O ALA 11 B 3.24

288. 13859 CE1 PHE 1418 A <--> 14048 OD1 ASP 8 B 3.72

289. 13859 CE1 PHE 1418 A <--> 14072 CB ALA 11 B 3.62

290. 13860 CE2 PHE 1418 A <--> 14073 C ALA 11 B 3.81

291. 13860 CE2 PHE 1418 A <--> 14074 O ALA 11 B 3.29

292. 13860 CE2 PHE 1418 A <--> 14072 CB ALA 11 B 3.71

293. 13861 CZ PHE 1418 A <--> 14072 CB ALA 11 B 3.52

294. 13868 CG LEU 1419 A <--> 14081 OE1 GLU 12 B 3.77

295. 13870 CD2 LEU 1419 A <--> 14080 CD GLU 12 B 3.61

296. 13870 CD2 LEU 1419 A <--> 14081 OE1 GLU 12 B 3.22

```
297.  13895  CG1 ILE 1422    A   <--> 14048  OD1 ASP    8    B      3.59
298.  13895  CG1 ILE 1422    A   <--> 14049  OD2 ASP    8    B      3.77
299.  13897  CD1 ILE 1422    A   <--> 14048  OD1 ASP    8    B      3.41
```

Salt bridges

```
       Atom Atom Res  Res              Atom Atom Res  Res
        no. name name no.  Chain        no. name name no.  Chain  Distance
  1.     43  OD2 ASP    5    A   <--> 14810  NZ  LYS   85    B      2.65
  2.   6127  NH1 ARG  632    A   <--> 14198  OD1 ASP   23    B      2.75
  3.   6583  NH2 ARG  676    A   <--> 14799  OE1 GLU   84    B      2.62
```

Number of salt bridges: 3

Number of hydrogen bonds: 18

Number of non-bonded contacts: 299

Supplementary material SM 5:
Vaccine MHC Class I receptor PDBsum interacting molecules

Hydrogen bonds

```
       <----- A T O M   1 ----->       <----- A T O M   2 ----->
       Atom Atom Res  Res              Atom Atom Res  Res
        no. name name no.  Chain        no. name name no.  Chain  Distance
  1.   1082  NH1 ARG  108    A   <-->  4584  O   ALA  201    B      3.11
  2.   1082  NH1 ARG  108    A   <-->  4723  OH  TYR  216    B      2.80
```

3. 1085 NH2 ARG 108 A <--> 4584 O ALA 201 B 2.86

4. 1085 NH2 ARG 108 A <--> 4601 O GLY 203 B 2.96

5. 1110 O LEU 110 A <--> 4411 N MET 182 B 3.13

6. 1120 NH1 ARG 111 A <--> 3915 O ARG 118 B 2.77

7. 1298 OE1 GLU 128 A <--> 4390 N GLY 179 B 2.69

8. 1627 OE1 GLU 161 A <--> 4609 N GLY 205 B 3.15

9. 1669 O GLU 166 A <--> 3707 NE2 HIS 95 B 2.92

10. 1704 NH1 ARG 169 A <--> 3710 O HIS 95 B 2.85

11. 1785 NZ LYS 176 A <--> 3208 OD1 ASN 45 B 2.72

12. 1785 NZ LYS 176 A <--> 3553 OE1 GLU 80 B 2.64

13. 1863 OD2 ASP 183 A <--> 3229 OG1 THR 48 B 2.68

14. 1892 N THR 187 A <--> 3273 OE2 GLU 52 B 2.76

15. 1900 O THR 187 A <--> 3118 NH1 ARG 36 B 2.86

16. 1896 OG1 THR 187 A <--> 3273 OE2 GLU 52 B 2.72

17. 1918 SD MET 189 A <--> 3121 NH2 ARG 36 B 3.06

Non-bonded contacts

<----- A T O M 1 -----> <----- A T O M 2 ----->

Atom Atom Res Res Atom Atom Res Res

no. name name no. Chain no. name name no. Chain Distance

1. 1 N GLY 1 A <--> 3760 SD MET 102 B 3.63

2. 1047 O SER 105 A <--> 3737 O ALA 99 B 3.68

3. 1047 O SER 105 A <--> 3757 CA MET 102 B 3.84

4. 1047 O SER 105 A <--> 3762 C MET 102 B 3.32

5. 1047 O SER 105 A <--> 3763 O MET 102 B 3.71

6. 1047 O SER 105 A <--> 3758 CB MET 102 B 3.28

7. 1047 O SER 105 A <--> 3759 CG MET 102 B 3.43

8. 1047 O SER 105 A <--> 3764 N ALA 103 B 3.21

9. 1047 O SER 105 A <--> 3766 CA ALA 103 B 3.61

10. 1043 CB SER 105 A <--> 3794 CB ALA 106 B 3.63

11. 1044 OG SER 105 A <--> 3794 CB ALA 106 B 3.56

12. 1056 O ASP 106 A <--> 3734 CA ALA 99 B 3.83

13. 1056 O ASP 106 A <--> 3737 O ALA 99 B 3.87

14. 1056 O ASP 106 A <--> 3735 CB ALA 99 B 3.20

15. 1060 CB TRP 107 A <--> 3734 CA ALA 99 B 3.66

16. 1060 CB TRP 107 A <--> 3735 CB ALA 99 B 3.43

17. 1061 CG TRP 107 A <--> 3734 CA ALA 99 B 3.74

18. 1062 CD1 TRP 107 A <--> 3760 SD MET 102 B 3.84

19. 1064 NE1 TRP 107 A <--> 3758 CB MET 102 B 3.56

20. 1064 NE1 TRP 107 A <--> 3760 SD MET 102 B 3.07

21. 1064 NE1 TRP 107 A <--> 3761 CE MET 102 B 3.51

22. 1069 CZ3 TRP 107 A <--> 3729 CB ALA 98 B 3.69

23. 1076 CB ARG 108 A <--> 4636 OH TYR 208 B 3.89

24. 1077 CG ARG 108 A <--> 4633 CE1 TYR 208 B 3.84

25. 1079 NE ARG 108 A <--> 4633 CE1 TYR 208 B 3.59

26. 1079 NE ARG 108 A <--> 4635 CZ TYR 208 B 3.84

27. 1081 CZ ARG 108 A <--> 4584 O ALA 201 B 3.24

28. 1081 CZ ARG 108 A <--> 4631 CD1 TYR 208 B 3.79

29. 1081 CZ ARG 108 A <--> 4633 CE1 TYR 208 B 3.84

30. 1081 CZ ARG 108 A <--> 4721 CE2 TYR 216 B 3.89

31. 1081 CZ ARG 108 A <--> 4723 OH TYR 216 B 3.77

32. 1082 NH1 ARG 108 A <--> 4584 O ALA 201 B 3.11

33. 1082 NH1 ARG 108 A <--> 4721 CE2 TYR 216 B 3.67

34. 1082 NH1 ARG 108 A <--> 4722 CZ TYR 216 B 3.67

35. 1082 NH1 ARG 108 A <--> 4723 OH TYR 216 B 2.80

36. 1085 NH2 ARG 108 A <--> 4584 O ALA 201 B 2.86

37. 1085 NH2 ARG 108 A <--> 4597 N GLY 203 B 3.11

38. 1085 NH2 ARG 108 A <--> 4599 CA GLY 203 B 3.52

39. 1085 NH2 ARG 108 A <--> 4600 C GLY 203 B 3.61

40. 1085 NH2 ARG 108 A <--> 4601 O GLY 203 B 2.96

41. 1085 NH2 ARG 108 A <--> 4630 CG TYR 208 B 3.75

42. 1085 NH2 ARG 108 A <--> 4632 CD2 TYR 208 B 3.64

43. 1085 NH2 ARG 108 A <--> 4634 CE2 TYR 208 B 3.74

44. 1085 NH2 ARG 108 A <--> 4721 CE2 TYR 216 B 3.21

45. 1090 N PHE 109 A <--> 4636 OH TYR 208 B 3.56

46. 1101 O PHE 109 A <--> 4414 CB MET 182 B 3.76

47. 1093 CB PHE 109 A <--> 4608 O PRO 204 B 3.31

48. 1096 CD2 PHE 109 A <--> 4605 CG PRO 204 B 3.47

49. 1110 O LEU 110 A <--> 4402 O SER 180 B 2.99

50. 1110 O LEU 110 A <--> 4405 CA CYS 181 B 3.60

51. 1110 O LEU 110 A <--> 4409 C CYS 181 B 3.85

52. 1110 O LEU 110 A <--> 4411 N MET 182 B 3.13

53. 1106 CG LEU 110 A <--> 4419 O MET 182 B 3.13

54. 1107 CD1 LEU 110 A <--> 4419 O MET 182 B 3.39

55. 1114 CB ARG 111 A <--> 4402 O SER 180 B 3.73

56. 1119 CZ ARG 111 A <--> 3915 O ARG 118 B 3.75

57. 1119 CZ ARG 111 A <--> 3918 CA GLY 119 B 3.84

58. 1120 NH1 ARG 111 A <--> 3914 C ARG 118 B 3.77

59. 1120 NH1 ARG 111 A <--> 3915 O ARG 118 B 2.77

60. 1120 NH1 ARG 111 A <--> 3918 CA GLY 119 B 3.39

61. 1120 NH1 ARG 111 A <--> 3925 CD PRO 120 B 3.87

62. 1120 NH1 ARG 111 A <--> 4406 CB CYS 181 B 3.52

63. 1123 NH2 ARG 111 A <--> 3915 O ARG 118 B 3.86

64. 1123 NH2 ARG 111 A <--> 3918 CA GLY 119 B 3.72

65. 1143 OH TYR 113 A <--> 3924 CG PRO 120 B 3.56

66. 1301 O GLU 128 A <--> 4398 CB SER 180 B 3.49

67. 1301 O GLU 128 A <--> 4399 OG SER 180 B 3.63

68. 1295 CB GLU 128 A <--> 4382 O GLY 177 B 3.41

69. 1297 CD GLU 128 A <--> 4390 N GLY 179 B 3.76

70. 1298 OE1 GLU 128 A <--> 4384 CA PRO 178 B 3.82

71. 1298 OE1 GLU 128 A <--> 4388 C PRO 178 B 3.69

72. 1298 OE1 GLU 128 A <--> 4390 N GLY 179 B 2.69

73. 1298 OE1 GLU 128 A <--> 4392 CA GLY 179 B 3.42

74. 1298 OE1 GLU 128 A <--> 4395 N SER 180 B 3.78

75. 1304 CA ASP 129 A <--> 4378 N GLY 177 B 3.89

76. 1305 CB ASP 129 A <--> 4373 CB PRO 176 B 3.90

77. 1305 CB ASP 129 A <--> 4378 N GLY 177 B 3.60

78. 1587 NH2 ARG 157 A <--> 4733 CA PRO 218 B 3.80

79. 1587 NH2 ARG 157 A <--> 4737 C PRO 218 B 3.79

80. 1587 NH2 ARG 157 A <--> 4734 CB PRO 218 B 3.46

81. 1587 NH2 ARG 157 A <--> 4739 N GLY 219 B 3.45

82. 1629 C GLU 161 A <--> 4611 CA GLY 205 B 3.53

83. 1630 O GLU 161 A <--> 4611 CA GLY 205 B 3.19

84. 1630 O GLU 161 A <--> 4618 CD PRO 206 B 3.51

85. 1624 CB GLU 161 A <--> 4609 N GLY 205 B 3.86

86. 1624 CB GLU 161 A <--> 4611 CA GLY 205 B 3.60

87. 1626 CD GLU 161 A <--> 4604 CB PRO 204 B 3.41

88. 1626 CD GLU 161 A <--> 4609 N GLY 205 B 3.79

89. 1627 OE1 GLU 161 A <--> 4604 CB PRO 204 B 3.47

90. 1627 OE1 GLU 161 A <--> 4609 N GLY 205 B 3.15

91. 1627 OE1 GLU 161 A <--> 4611 CA GLY 205 B 3.80

92. 1627 OE1 GLU 161 A <--> 4613 O GLY 205 B 3.82

93. 1627 OE1 GLU 161 A <--> 4733 CA PRO 218 B 3.88

94. 1627 OE1 GLU 161 A <--> 4734 CB PRO 218 B 3.34

95. 1628 OE2 GLU 161 A <--> 4604 CB PRO 204 B 3.42

96. 1633 CA GLY 162 A <--> 4614 N PRO 206 B 3.88

97. 1633 CA GLY 162 A <--> 4618 CD PRO 206 B 3.68

98. 1662 CA GLU 166 A <--> 3706 CE1 HIS 95 B 3.75

99. 1662 CA GLU 166 A <--> 3707 NE2 HIS 95 B 3.81

100. 1668 C GLU 166 A <--> 3707 NE2 HIS 95 B 3.73

101. 1669 O GLU 166 A <--> 3706 CE1 HIS 95 B 3.70

102. 1669 O GLU 166 A <--> 3707 NE2 HIS 95 B 2.92

103. 1664 CG GLU 166 A <--> 3706 CE1 HIS 95 B 3.75

104. 1667 OE2 GLU 166 A <--> 4616 CB PRO 206 B 3.70

105. 1698 CB ARG 169 A <--> 3705 CD2 HIS 95 B 3.86

106. 1698 CB ARG 169 A <--> 3707 NE2 HIS 95 B 3.43

107. 1700 CD ARG 169 A <--> 3710 O HIS 95 B 3.25

108. 1700 CD ARG 169 A <--> 3706 CE1 HIS 95 B 3.71

109. 1701 NE ARG 169 A <--> 3710 O HIS 95 B 3.87

110. 1703 CZ ARG 169 A <--> 3710 O HIS 95 B 3.72

111. 1703 CZ ARG 169 A <--> 3735 CB ALA 99 B 3.79

112. 1704 NH1 ARG 169 A <--> 3709 C HIS 95 B 3.75

113. 1704 NH1 ARG 169 A <--> 3710 O HIS 95 B 2.85

114. 1704 NH1 ARG 169 A <--> 3713 CA ALA 96 B 3.57

115. 1707 NH2 ARG 169 A <--> 3735 CB ALA 99 B 3.61

116. 1759 OE2 GLU 173 A <--> 3694 CB PRO 94 B 3.81

117. 1783 CD LYS 176 A <--> 3208 OD1 ASN 45 B 3.77

118. 1784 CE LYS 176 A <--> 3208 OD1 ASN 45 B 3.67

119. 1785 NZ LYS 176 A <--> 3207 CG ASN 45 B 3.47

120. 1785 NZ LYS 176 A <--> 3208 OD1 ASN 45 B 2.72

121. 1785 NZ LYS 176 A <--> 3209 ND2 ASN 45 B 3.50

122. 1785 NZ LYS 176 A <--> 3552 CD GLU 80 B 3.83

123. 1785 NZ LYS 176 A <--> 3553 OE1 GLU 80 B 2.64

124. 1793 CA GLU 177 A <--> 3213 O ASN 45 B 3.83

125. 1800 O GLU 177 A <--> 3218 O GLY 46 B 3.28

126. 1794 CB GLU 177 A <--> 3213 O ASN 45 B 3.25

127. 1794 CB GLU 177 A <--> 3216 CA GLY 46 B 3.61

128. 1824 CD GLN 180 A <--> 3553 OE1 GLU 80 B 3.30

129. 1825 OE1 GLN 180 A <--> 3553 OE1 GLU 80 B 3.28

130. 1826 NE2 GLN 180 A <--> 3552 CD GLU 80 B 3.74

131. 1826 NE2 GLN 180 A <--> 3553 OE1 GLU 80 B 3.26

132. 1826 NE2 GLN 180 A <--> 3761 CE MET 102 B 3.28

133. 1834 CB ARG 181 A <--> 3218 O GLY 46 B 3.47

134. 1834 CB ARG 181 A <--> 3222 CB ALA 47 B 3.66

135. 1836 CD ARG 181 A <--> 3218 O GLY 46 B 3.38

136. 1837 NE ARG 181 A <--> 3218 O GLY 46 B 3.47

137. 1839 CZ ARG 181 A <--> 3218 O GLY 46 B 3.45

138. 1840 NH1 ARG 181 A <--> 3218 O GLY 46 B 3.28

139. 1843 NH2 ARG 181 A <--> 3229 OG1 THR 48 B 3.28

140. 1859 CA ASP 183 A <--> 3233 O THR 48 B 3.36

141. 1860 CB ASP 183 A <--> 3233 O THR 48 B 3.85

142. 1861 CG ASP 183 A <--> 3233 O THR 48 B 3.76

143. 1861 CG ASP 183 A <--> 3229 OG1 THR 48 B 3.86

144. 1863 OD2 ASP 183 A <--> 3225 N THR 48 B 3.36

145. 1863 OD2 ASP 183 A <--> 3227 CA THR 48 B 3.73

146. 1863 OD2 ASP 183 A <--> 3233 O THR 48 B 3.58

147. 1863 OD2 ASP 183 A <--> 3228 CB THR 48 B 3.26

148. 1863 OD2 ASP 183 A <--> 3229 OG1 THR 48 B 2.68

149. 1866 N ALA 184 A <--> 3233 O THR 48 B 3.84

150. 1870 C ALA 184 A <--> 3249 CB GLN 50 B 3.82

151. 1871 O ALA 184 A <--> 3249 CB GLN 50 B 3.25

152. 1871 O ALA 184 A <--> 3250 CG GLN 50 B 3.84

153.	1869	CB	ALA	184	A	<-->	3246	N	GLN	50	B	3.60
154.	1869	CB	ALA	184	A	<-->	3257	O	GLN	50	B	3.30
155.	1869	CB	ALA	184	A	<-->	3249	CB	GLN	50	B	3.83
156.	1877	C	PRO	185	A	<-->	3250	CG	GLN	50	B	3.71
157.	1878	O	PRO	185	A	<-->	3250	CG	GLN	50	B	3.17
158.	1878	O	PRO	185	A	<-->	3273	OE2	GLU	52	B	3.53
159.	1890	C	LYS	186	A	<-->	3273	OE2	GLU	52	B	3.81
160.	1882	CB	LYS	186	A	<-->	3141	SD	MET	38	B	3.77
161.	1883	CG	LYS	186	A	<-->	3250	CG	GLN	50	B	3.74
162.	1883	CG	LYS	186	A	<-->	3251	CD	GLN	50	B	3.62
163.	1883	CG	LYS	186	A	<-->	3252	OE1	GLN	50	B	3.78
164.	1885	CE	LYS	186	A	<-->	3141	SD	MET	38	B	3.65
165.	1885	CE	LYS	186	A	<-->	3252	OE1	GLN	50	B	3.56
166.	1892	N	THR	187	A	<-->	3270	CG	GLU	52	B	3.85
167.	1892	N	THR	187	A	<-->	3271	CD	GLU	52	B	3.70
168.	1892	N	THR	187	A	<-->	3273	OE2	GLU	52	B	2.76
169.	1894	CA	THR	187	A	<-->	3273	OE2	GLU	52	B	3.41
170.	1899	C	THR	187	A	<-->	3118	NH1	ARG	36	B	3.75
171.	1900	O	THR	187	A	<-->	3114	CD	ARG	36	B	3.23
172.	1900	O	THR	187	A	<-->	3117	CZ	ARG	36	B	3.84
173.	1900	O	THR	187	A	<-->	3118	NH1	ARG	36	B	2.86
174.	1900	O	THR	187	A	<-->	3273	OE2	GLU	52	B	3.71
175.	1895	CB	THR	187	A	<-->	3273	OE2	GLU	52	B	3.20
176.	1896	OG1	THR	187	A	<-->	3273	OE2	GLU	52	B	2.72
177.	1903	CA	HIS	188	A	<-->	3118	NH1	ARG	36	B	3.85

178. 1904 CB HIS 188 A <--> 3142 CE MET 38 B 3.70

179. 1917 CG MET 189 A <--> 3118 NH1 ARG 36 B 3.61

180. 1917 CG MET 189 A <--> 3121 NH2 ARG 36 B 3.79

181. 1918 SD MET 189 A <--> 3117 CZ ARG 36 B 3.77

182. 1918 SD MET 189 A <--> 3118 NH1 ARG 36 B 3.60

183. 1918 SD MET 189 A <--> 3121 NH2 ARG 36 B 3.06

184. 2661 O GLY 265 A <--> 3241 CE1 PHE 49 B 3.45

185. 2703 CD1 LEU 270 A <--> 3272 OE1 GLU 52 B 3.56

186. 2703 CD1 LEU 270 A <--> 3273 OE2 GLU 52 B 3.77

187. 2719 CB LEU 272 A <--> 3118 NH1 ARG 36 B 3.82

188. 2720 CG LEU 272 A <--> 3118 NH1 ARG 36 B 3.83

189. 2721 CD1 LEU 272 A <--> 3118 NH1 ARG 36 B 3.36

190. 2722 CD2 LEU 272 A <--> 3118 NH1 ARG 36 B 3.79

191. 2752 CE3 TRP 274 A <--> 3121 NH2 ARG 36 B 3.57

192. 2753 CZ2 TRP 274 A <--> 3108 O LYS 35 B 3.81

193. 2754 CZ3 TRP 274 A <--> 3108 O LYS 35 B 3.84

194. 2754 CZ3 TRP 274 A <--> 3121 NH2 ARG 36 B 3.66

195. 2755 CH2 TRP 274 A <--> 3098 CA LYS 35 B 3.70

196. 2755 CH2 TRP 274 A <--> 3107 C LYS 35 B 3.74

197. 2755 CH2 TRP 274 A <--> 3108 O LYS 35 B 3.28

198. 2767 O GLU 275 A <--> 3299 CB SER 56 B 3.46

199. 2767 O GLU 275 A <--> 3300 OG SER 56 B 3.45

Salt bridges

\------------

<----- A T O M 1 -----> <----- A T O M 2 ----->

Atom Atom Res Res Atom Atom Res Res

no. name name no. Chain no. name name no. Chain Distance

1. 1785 NZ LYS 176 A <--> 3554 OE2 GLU 80 B 2.64

Number of salt bridges: 1

Number of hydrogen bonds: 17

Number of non-bonded contacts: 199

Supplementary material SM 6:
Vaccine MHC Class II receptor PDBsum interacting molecules
Hydrogen bonds

<----- A T O M 1 -----> <----- A T O M 2 ----->

Atom Atom Res Res Atom Atom Res Res

no. name name no. Chain no. name name no. Chain Distance

1. 725 O ARG 74 A <--> 3696 N GLN 4 B 3.26

2. 739 OD1 ASN 76 A <--> 3696 N GLN 4 B 3.14

3. 788 OG1 THR 81 A <--> 4111 OD1 ASN 45 B 3.09

4. 799 ND2 ASN 82 A <--> 4494 OE2 GLU 84 B 2.72

5. 1736 OE2 GLU 177 A <--> 5764 N ALA 237 B 2.88

6. 1757 OD2 ASP 179 A <--> 5783 OG1 THR 239 B 2.63

7. 2306 O GLU 230 A <--> 3714 ND2 ASN 5 B 2.97

8. 2330 NH1 ARG 233 A <--> 3789 OH TYR 13 B 2.96

9. 2850 NZ LYS 283 A <--> 5613 OE2 GLU 215 B 2.88

10. 2900 NE2 GLN 288 A <--> 7857 OE2 GLU 489 B 2.83

11.	2922	ND1	HIS	290	A	<-->	6134	ND2	ASN	281	B	2.95
12.	3174	O	GLU	315	A	<-->	5960	N	GLY	261	B	2.91
13.	3438	NH1	ARG	344	A	<-->	7838	O	TRP	486	B	2.91

Non-bonded contacts

	<----- A T O M 1 ----->						<----- A T O M 2 ----->					
	Atom no.	Atom name	Res name	Res no.	Chain		Atom no.	Atom name	Res name	Res no.	Chain	Distance
1.	686	O	MET	71	A	<-->	3686	CG2	THR	2	B	3.71
2.	683	SD	MET	71	A	<-->	3686	CG2	THR	2	B	3.87
3.	724	C	ARG	74	A	<-->	3686	CG2	THR	2	B	3.73
4.	725	O	ARG	74	A	<-->	3682	CA	THR	2	B	3.39
5.	725	O	ARG	74	A	<-->	3687	C	THR	2	B	3.44
6.	725	O	ARG	74	A	<-->	3683	CB	THR	2	B	3.37
7.	725	O	ARG	74	A	<-->	3686	CG2	THR	2	B	3.57
8.	725	O	ARG	74	A	<-->	3689	N	PRO	3	B	3.62
9.	725	O	ARG	74	A	<-->	3693	CD	PRO	3	B	3.84
10.	725	O	ARG	74	A	<-->	3696	N	GLN	4	B	3.26
11.	725	O	ARG	74	A	<-->	3698	CA	GLN	4	B	3.75
12.	725	O	ARG	74	A	<-->	3699	CB	GLN	4	B	3.39
13.	725	O	ARG	74	A	<-->	3708	N	ASN	5	B	3.57
14.	712	CB	ARG	74	A	<-->	3702	OE1	GLN	4	B	3.82
15.	713	CG	ARG	74	A	<-->	3686	CG2	THR	2	B	3.80

16. 726 N SER 75 A <--> 3686 CG2 THR 2 B 3.77
17. 728 CA SER 75 A <--> 3679 O MET 1 B 3.47
18. 728 CA SER 75 A <--> 3682 CA THR 2 B 3.67
19. 728 CA SER 75 A <--> 3686 CG2 THR 2 B 3.67
20. 728 CA SER 75 A <--> 3693 CD PRO 3 B 3.64
21. 732 C SER 75 A <--> 3693 CD PRO 3 B 3.47
22. 733 O SER 75 A <--> 3679 O MET 1 B 3.83
23. 733 O SER 75 A <--> 3692 CG PRO 3 B 3.84
24. 733 O SER 75 A <--> 3693 CD PRO 3 B 3.27
25. 729 CB SER 75 A <--> 3679 O MET 1 B 3.09
26. 729 CB SER 75 A <--> 3686 CG2 THR 2 B 3.70
27. 738 CG ASN 76 A <--> 3691 CB PRO 3 B 3.65
28. 738 CG ASN 76 A <--> 3692 CG PRO 3 B 3.81
29. 739 OD1 ASN 76 A <--> 3691 CB PRO 3 B 3.70
30. 739 OD1 ASN 76 A <--> 3696 N GLN 4 B 3.14
31. 739 OD1 ASN 76 A <--> 3698 CA GLN 4 B 3.72
32. 739 OD1 ASN 76 A <--> 3699 CB GLN 4 B 3.27
33. 740 ND2 ASN 76 A <--> 3691 CB PRO 3 B 3.66
34. 765 CG2 THR 78 A <--> 4099 CE LYS 44 B 3.90
35. 765 CG2 THR 78 A <--> 4100 NZ LYS 44 B 3.70
36. 787 CB THR 81 A <--> 4111 OD1 ASN 45 B 3.84
37. 788 OG1 THR 81 A <--> 4108 CA ASN 45 B 3.88
38. 788 OG1 THR 81 A <--> 4110 CG ASN 45 B 3.83
39. 788 OG1 THR 81 A <--> 4111 OD1 ASN 45 B 3.09
40. 790 CG2 THR 81 A <--> 4428 OH TYR 77 B 3.27

41. 796 CB ASN 82 A <--> 4494 OE2 GLU 84 B 3.28

42. 797 CG ASN 82 A <--> 4494 OE2 GLU 84 B 3.45

43. 799 ND2 ASN 82 A <--> 4494 OE2 GLU 84 B 2.72

44. 825 O PRO 85 A <--> 4597 CB PRO 94 B 3.78

45. 825 O PRO 85 A <--> 4598 CG PRO 94 B 3.62

46. 829 CB GLU 86 A <--> 4605 CB HIS 95 B 3.70

47. 830 CG GLU 86 A <--> 4605 CB HIS 95 B 3.56

48. 833 OE2 GLU 86 A <--> 4605 CB HIS 95 B 3.55

49. 843 O VAL 87 A <--> 4608 CD2 HIS 95 B 3.40

50. 843 O VAL 87 A <--> 4610 NE2 HIS 95 B 3.36

51. 1533 CG1 VAL 158 A <--> 5738 CD PRO 232 B 3.81

52. 1534 CG2 VAL 158 A <--> 5740 O PRO 232 B 3.22

53. 1556 OD1 ASP 160 A <--> 5750 CD PRO 234 B 3.25

54. 1662 O GLU 170 A <--> 4568 ND2 ASN 91 B 3.79

55. 1657 CG GLU 170 A <--> 4566 CG ASN 91 B 3.57

56. 1657 CG GLU 170 A <--> 4568 ND2 ASN 91 B 3.35

57. 1658 CD GLU 170 A <--> 4565 CB ASN 91 B 3.83

58. 1658 CD GLU 170 A <--> 4566 CG ASN 91 B 3.84

59. 1658 CD GLU 170 A <--> 4568 ND2 ASN 91 B 3.57

60. 1659 OE1 GLU 170 A <--> 4568 ND2 ASN 91 B 3.57

61. 1660 OE2 GLU 170 A <--> 4565 CB ASN 91 B 3.88

62. 1669 O PRO 171 A <--> 4568 ND2 ASN 91 B 3.62

63. 1675 CD1 LEU 172 A <--> 4572 O ASN 91 B 3.62

64. 1675 CD1 LEU 172 A <--> 4568 ND2 ASN 91 B 3.87

65. 1676 CD2 LEU 172 A <--> 4599 CD PRO 94 B 3.82

66. 1704 CB HIS 175 A <--> 5752 O PRO 234 B 3.58

67. 1704 CB HIS 175 A <--> 5750 CD PRO 234 B 3.87

68. 1704 CB HIS 175 A <--> 5757 O GLY 235 B 3.33

69. 1705 CG HIS 175 A <--> 5756 C GLY 235 B 3.86

70. 1705 CG HIS 175 A <--> 5757 O GLY 235 B 3.26

71. 1706 ND1 HIS 175 A <--> 5743 CA GLY 233 B 3.26

72. 1706 ND1 HIS 175 A <--> 5744 C GLY 233 B 3.57

73. 1706 ND1 HIS 175 A <--> 5746 N PRO 234 B 3.62

74. 1706 ND1 HIS 175 A <--> 5750 CD PRO 234 B 3.49

75. 1707 CD2 HIS 175 A <--> 5757 O GLY 235 B 3.26

76. 1707 CD2 HIS 175 A <--> 5760 CA ALA 236 B 3.61

77. 1707 CD2 HIS 175 A <--> 5762 C ALA 236 B 3.82

78. 1707 CD2 HIS 175 A <--> 5764 N ALA 237 B 3.54

79. 1708 CE1 HIS 175 A <--> 5741 N GLY 233 B 3.75

80. 1708 CE1 HIS 175 A <--> 5743 CA GLY 233 B 3.57

81. 1709 NE2 HIS 175 A <--> 5760 CA ALA 236 B 3.40

82. 1709 NE2 HIS 175 A <--> 5764 N ALA 237 B 3.62

83. 1733 CG GLU 177 A <--> 5767 CB ALA 237 B 3.65

84. 1734 CD GLU 177 A <--> 5764 N ALA 237 B 3.82

85. 1735 OE1 GLU 177 A <--> 5713 CG1 VAL 229 B 3.58

86. 1736 OE2 GLU 177 A <--> 5731 CA GLY 231 B 3.70

87. 1736 OE2 GLU 177 A <--> 5760 CA ALA 236 B 3.52

88. 1736 OE2 GLU 177 A <--> 5762 C ALA 236 B 3.67

89. 1736 OE2 GLU 177 A <--> 5761 CB ALA 236 B 3.60

90. 1736 OE2 GLU 177 A <--> 5764 N ALA 237 B 2.88

91. 1736 OE2 GLU 177 A <--> 5766 CA ALA 237 B 3.83

92. 1736 OE2 GLU 177 A <--> 5767 CB ALA 237 B 3.76

93. 1755 CG ASP 179 A <--> 5783 OG1 THR 239 B 3.73

94. 1757 OD2 ASP 179 A <--> 5782 CB THR 239 B 3.48

95. 1757 OD2 ASP 179 A <--> 5783 OG1 THR 239 B 2.63

96. 1757 OD2 ASP 179 A <--> 5785 CG2 THR 239 B 3.51

97. 2120 CB GLN 212 A <--> 4116 O ASN 45 B 3.70

98. 2120 CB GLN 212 A <--> 4112 ND2 ASN 45 B 3.89

99. 2122 CD GLN 212 A <--> 4111 OD1 ASN 45 B 3.79

100. 2123 OE1 GLN 212 A <--> 4110 CG ASN 45 B 3.71

101. 2123 OE1 GLN 212 A <--> 4111 OD1 ASN 45 B 3.12

102. 2132 CB GLU 213 A <--> 4121 O GLY 46 B 3.33

103. 2133 CG GLU 213 A <--> 4121 O GLY 46 B 3.60

104. 2291 CB THR 229 A <--> 4132 OG1 THR 48 B 3.64

105. 2294 CG2 THR 229 A <--> 4131 CB THR 48 B 3.58

106. 2294 CG2 THR 229 A <--> 4132 OG1 THR 48 B 3.46

107. 2306 O GLU 230 A <--> 3714 ND2 ASN 5 B 2.97

108. 2301 CG GLU 230 A <--> 4059 CG2 ILE 40 B 3.88

109. 2302 CD GLU 230 A <--> 4060 CD1 ILE 40 B 3.58

110. 2303 OE1 GLU 230 A <--> 3751 CD1 LEU 9 B 3.80

111. 2303 OE1 GLU 230 A <--> 4060 CD1 ILE 40 B 3.62

112. 2304 OE2 GLU 230 A <--> 4060 CD1 ILE 40 B 3.79

113. 2307 N LEU 231 A <--> 3677 CE MET 1 B 3.42

114. 2309 CA LEU 231 A <--> 3677 CE MET 1 B 3.89

115. 2309 CA LEU 231 A <--> 3684 OG1 THR 2 B 3.80

116. 2309 CA LEU 231 A <--> 3714 ND2 ASN 5 B 3.78

117. 2314 C LEU 231 A <--> 3714 ND2 ASN 5 B 3.68

118. 2315 O LEU 231 A <--> 3684 OG1 THR 2 B 3.79

119. 2315 O LEU 231 A <--> 3714 ND2 ASN 5 B 3.21

120. 2311 CG LEU 231 A <--> 3676 SD MET 1 B 3.61

121. 2311 CG LEU 231 A <--> 3684 OG1 THR 2 B 3.79

122. 2312 CD1 LEU 231 A <--> 3673 CA MET 1 B 3.83

123. 2313 CD2 LEU 231 A <--> 3684 OG1 THR 2 B 3.49

124. 2326 CD ARG 233 A <--> 3752 CD2 LEU 9 B 3.74

125. 2326 CD ARG 233 A <--> 3789 OH TYR 13 B 3.33

126. 2329 CZ ARG 233 A <--> 3789 OH TYR 13 B 3.84

127. 2330 NH1 ARG 233 A <--> 3787 CE2 TYR 13 B 3.57

128. 2330 NH1 ARG 233 A <--> 3788 CZ TYR 13 B 3.45

129. 2330 NH1 ARG 233 A <--> 3789 OH TYR 13 B 2.96

130. 2341 CG PRO 234 A <--> 3713 OD1 ASN 5 B 3.14

131. 2342 CD PRO 234 A <--> 3713 OD1 ASN 5 B 3.69

132. 2342 CD PRO 234 A <--> 3752 CD2 LEU 9 B 3.85

133. 2367 OE2 GLU 237 A <--> 3789 OH TYR 13 B 3.43

134. 2846 CB LYS 283 A <--> 5797 OG1 THR 241 B 3.64

135. 2847 CG LYS 283 A <--> 5797 OG1 THR 241 B 3.78

136. 2848 CD LYS 283 A <--> 5797 OG1 THR 241 B 3.65

137. 2848 CD LYS 283 A <--> 5799 CG2 THR 241 B 3.75

138. 2849 CE LYS 283 A <--> 5613 OE2 GLU 215 B 3.65

139. 2850 NZ LYS 283 A <--> 5611 CD GLU 215 B 3.32

140. 2850 NZ LYS 283 A <--> 5612 OE1 GLU 215 B 3.00

141. 2850 NZ LYS 283 A <--> 5613 OE2 GLU 215 B 2.88

142. 2850 NZ LYS 283 A <--> 5792 O GLY 240 B 3.46

143. 2869 CG GLN 285 A <--> 5799 CG2 THR 241 B 3.78

144. 2898 CD GLN 288 A <--> 7857 OE2 GLU 489 B 3.61

145. 2899 OE1 GLN 288 A <--> 7857 OE2 GLU 489 B 3.57

146. 2900 NE2 GLN 288 A <--> 7855 CD GLU 489 B 3.24

147. 2900 NE2 GLN 288 A <--> 7856 OE1 GLU 489 B 3.41

148. 2900 NE2 GLN 288 A <--> 7857 OE2 GLU 489 B 2.83

149. 2920 CB HIS 290 A <--> 6134 ND2 ASN 281 B 3.84

150. 2921 CG HIS 290 A <--> 6134 ND2 ASN 281 B 3.67

151. 2922 ND1 HIS 290 A <--> 6132 CG ASN 281 B 3.74

152. 2922 ND1 HIS 290 A <--> 6133 OD1 ASN 281 B 3.69

153. 2922 ND1 HIS 290 A <--> 6134 ND2 ASN 281 B 2.95

154. 2923 CD2 HIS 290 A <--> 6109 NE ARG 279 B 3.83

155. 2923 CD2 HIS 290 A <--> 6111 CZ ARG 279 B 3.67

156. 2923 CD2 HIS 290 A <--> 6115 NH2 ARG 279 B 3.65

157. 2924 CE1 HIS 290 A <--> 6134 ND2 ASN 281 B 3.80

158. 2925 NE2 HIS 290 A <--> 5801 O THR 241 B 3.65

159. 2925 NE2 HIS 290 A <--> 6109 NE ARG 279 B 3.69

160. 2925 NE2 HIS 290 A <--> 6111 CZ ARG 279 B 3.90

161. 2945 CD1 LEU 292 A <--> 5664 CG2 VAL 222 B 3.70

162. 3157 CG GLN 314 A <--> 5985 CZ ARG 264 B 3.82

163. 3157 CG GLN 314 A <--> 5989 NH2 ARG 264 B 3.76

164. 3159 OE1 GLN 314 A <--> 5962 CA GLY 261 B 3.26

165. 3159 OE1 GLN 314 A <--> 5963 C GLY 261 B 3.80

166. 3159 OE1 GLN 314 A <--> 5965 N PRO 262 B 3.77

167. 3159 OE1 GLN 314 A <--> 5969 CD PRO 262 B 3.31

168. 3159 OE1 GLN 314 A <--> 5983 NE ARG 264 B 3.90

169. 3174 O GLU 315 A <--> 5954 CA PRO 260 B 3.10

170. 3174 O GLU 315 A <--> 5958 C PRO 260 B 3.49

171. 3174 O GLU 315 A <--> 5955 CB PRO 260 B 3.58

172. 3174 O GLU 315 A <--> 5960 N GLY 261 B 2.91

173. 3177 CA GLU 316 A <--> 5955 CB PRO 260 B 3.82

174. 3180 CD GLU 316 A <--> 5955 CB PRO 260 B 3.48

175. 3181 OE1 GLU 316 A <--> 5955 CB PRO 260 B 3.40

176. 3181 OE1 GLU 316 A <--> 5956 CG PRO 260 B 3.86

177. 3182 OE2 GLU 316 A <--> 5955 CB PRO 260 B 3.27

178. 3196 C LYS 317 A <--> 5280 O PRO 176 B 3.35

179. 3197 O LYS 317 A <--> 5279 C PRO 176 B 3.64

180. 3197 O LYS 317 A <--> 5280 O PRO 176 B 3.20

181. 3197 O LYS 317 A <--> 5277 CG PRO 176 B 3.84

182. 3188 CB LYS 317 A <--> 5950 CA GLY 259 B 3.88

183. 3188 CB LYS 317 A <--> 5951 C GLY 259 B 3.58

184. 3188 CB LYS 317 A <--> 5952 O GLY 259 B 3.58

185. 3189 CG LYS 317 A <--> 5952 O GLY 259 B 3.70

186. 3190 CD LYS 317 A <--> 5283 CA GLY 177 B 3.88

187. 3198 N ALA 318 A <--> 5280 O PRO 176 B 3.43

188. 3198 N ALA 318 A <--> 5957 CD PRO 260 B 3.90

189. 3200 CA ALA 318 A <--> 5280 O PRO 176 B 3.36

190. 3201 CB ALA 318 A <--> 5280 O PRO 176 B 3.86

191. 3201 CB ALA 318 A <--> 5916 CB SER 256 B 3.84

192. 3201 CB ALA 318 A <--> 5917 OG SER 256 B 3.63

193. 3201 CB ALA 318 A <--> 5957 CD PRO 260 B 3.90

194. 3204 N GLY 319 A <--> 5277 CG PRO 176 B 3.46

195. 3204 N GLY 319 A <--> 5278 CD PRO 176 B 3.71

196. 3216 O VAL 320 A <--> 5277 CG PRO 176 B 3.53

197. 3221 CG1 VAL 321 A <--> 5504 O GLY 203 B 3.57

198. 3221 CG1 VAL 321 A <--> 5642 N GLY 219 B 3.82

199. 3222 CG2 VAL 321 A <--> 5642 N GLY 219 B 3.59

200. 3222 CG2 VAL 321 A <--> 5646 O GLY 219 B 3.85

201. 3232 O SER 322 A <--> 5506 CA PRO 204 B 3.21

202. 3232 O SER 322 A <--> 5510 C PRO 204 B 3.77

203. 3232 O SER 322 A <--> 5511 O PRO 204 B 3.68

204. 3232 O SER 322 A <--> 5507 CB PRO 204 B 3.41

205. 3239 CG2 THR 323 A <--> 5520 CG PRO 206 B 3.80

206. 3400 CD GLU 340 A <--> 5648 CA PRO 220 B 3.60

207. 3401 OE1 GLU 340 A <--> 5648 CA PRO 220 B 3.27

208. 3401 OE1 GLU 340 A <--> 5649 CB PRO 220 B 3.59

209. 3402 OE2 GLU 340 A <--> 5648 CA PRO 220 B 3.64

210. 3402 OE2 GLU 340 A <--> 5652 C PRO 220 B 3.70

211. 3435 NE ARG 344 A <--> 7833 CE3 TRP 486 B 3.68

212. 3437 CZ ARG 344 A <--> 7838 O TRP 486 B 3.84

213. 3437 CZ ARG 344 A <--> 7833 CE3 TRP 486 B 3.74

214. 3438 NH1 ARG 344 A <--> 7837 C TRP 486 B 3.71

215. 3438 NH1 ARG 344 A <--> 7838 O TRP 486 B 2.91

216.   3441 NH2 ARG 344   A   <--> 6154 ND2 ASN 283   B   3.43

Salt bridges

\------------

<----- A T O M   1 ----->   <----- A T O M   2 ----->

Atom Atom Res Res   Atom Atom Res Res

no. name name no. Chain   no. name name no. Chain Distance

1.   2850 NZ LYS 283   A   <--> 5612 OE1 GLU 215   B   2.88

Number of salt bridges: 1

Number of hydrogen bonds: 13

Number of non-bonded contacts: 216
