# Supplementary material for: In silico designing of vaccine candidate against Clostridium difficile
Source: Sci Rep. 2021 Jul 9;11:14215. doi: 10.1038/s41598-021-93305-6 (PMC8271013; doi:10.1038/s41598-021-93305-6)
Supplement: Supplementary file 3 — Supplementary Information 3. [file 41598_2021_93305_MOESM3_ESM.pdf]

In silico designing of vaccine candidate against *Clostridium difficile*

Srijita Basak[1*], Debashrito Deb[1*], Utkarsh Narsaria[1*], Tamalika Kar[1*], Filippo Castiglione[3], Indraneel Sanyal[1], Pratap D. Bade[1†], Anurag P. Srivastava[1†]

[1]Biopharmaceutical Development Department

Syngene International Limited, Bangalore

India

[2]Institute for Applied Computing (IAC),

National Research Council of Italy,

Rome, Italy

*These authors contributed equally to this work.

†Corresponding Author

Anurag P. Srivastava: Anurag.Srivastava@syngeneintl.com, anuiitkgp@gmail.com

Phone: +91-9108720555

ORCID ID: 0000-0003-3493-1375

Pratap D. Bade: Pratap.Bade@syngeneintl.com

Phone: +91-8884522554

Supplementary Tables

Supplementary Table S1:

All the CTL epitopes of FliC as predicted by NetCTL 1.2

CTL Epitope prediction by NetCTL-1.2 Server					
**MHC Super type**	**Epitopes**	**Position**	**Prediction Score**	**Immunogenic Score**	**Antigenicity Score**
MHC supertype A1. Threshold 0.750000	DTDVASEMV	251	1.7931	-0.08699	1.0392
	GTDAAKTMV	196	1.5102	-0.18817	0.8325
	TSSTIRLQV	139	0.9164	0.08664	1.5090
	GTSNNNNEI	157	0.9150	-0.01956	1.0474
	TTASIGSMK	182	0.7958	-0.23449	0.5482
	ASEMVNLSK	255	0.7846	-0.21611	-0.5103
MHC supertype A2. Threshold 0.750000	KLLDGTSST	134	1.1834	-0.14802	1.1140
	KIADELTQL	109	1.1417	0.08716	0.1042
	NLSKMNILV	260	1.1094	-0.34195	0.3060
	SLDAALKSL	206	1.0206	-0.20443	0.5903
	NLNNTLENV	234	0.9200	0.09947	0.1055
	LLDGTSSTI	135	0.8934	-0.19357	0.7046
	QLKDEIERI	116	0.8467	0.28984	-0.0886
	SMEKLSSGV	26	0.8025	-0.47085	0.5353
MHC supertype A3. Threshold 0.750000	KLSSGVRIK	29	1.4160	-0.02687	1.7450
	TTASIGSMK	182	1.3955	-0.23449	0.5482
	KSLNSSRAK	212	1.3136	-0.2604	1.2987
	VSSLDAALK	204	1.0599	0.02011	0.7290
	ALKSLNSSR	210	0.9532	-0.48964	0.4458
	LIANNQMGR	10	0.8831	-0.23755	0.6154
	IADELTQLK	110	0.8594	0.02945	-0.0526
	LSSGVRIKR	30	0.8417	0.05564	1.0298
	ASEMVNLSK	255	0.8184	-0.21611	-0.5103
MHC supertype A24. Threshold 0.750000	IQLVNTASI	167	0.7619	0.00454	0.3822
MHC supertype A26. Threshold 0.750000	ETGNILQRM	82	1.9134	0.05613	-0.0994
	DVASEMVNL	253	1.0353	-0.19051	0.6327
	ITTASIGSM	181	0.7617	-0.05191	0.3786
MHC supertype B7. Threshold 0.750000	RVNTNVSAL	2	1.5911	-0.04724	1.0338
	LQRMRTLSL	87	1.2268	-0.17898	-0.3935
	AAKTMVSSL	199	1.0971	-0.39936	0.1802
	MVSSLDAAL	203	0.9625	-0.15421	0.6392
	LVQASQSML	267	0.9582	-0.51059	-0.3902
	RAADDAAGL	38	0.8838	0.14627	1.0819
	VVQTAEGSL	72	0.8458	0.06575	0.4709
	SAGITTASI	178	0.7699	0.15562	0.9998
MHC supertype B8. Threshold 0.750000	NILQRMRTL	85	1.2415	-0.1687	-1.1378
	MVSSLDAAL	203	1.1410	-0.15421	0.6392
	LQRMRTLSL	87	1.0792	-0.17898	-0.3935
	KMRAQIKGL	51	0.7771	-0.09355	0.4497
MHC supertype B27. Threshold 0.750000	IRLQVGASY	143	1.7376	-0.1117	1.3203
	GRNVNGQSK	17	1.3369	-0.12938	2.6255
	ERISSSTEF	122	1.1820	-0.34884	0.2455
	MRVNTNVSA	1	0.9137	-0.02322	1.3220
	SRAKLGAQQ	217	0.7922	-0.21786	1.0019
MHC supertype B39. Threshold 0.750000	SLEETGNIL	79	1.5235	0.27525	-0.3372
	TQNNLNNTL	231	1.3949	-0.00828	0.2517
	EREKIADEL	106	1.2476	0.05915	0.3808
	QQPQGVLQL	280	0.7734	-0.12534	0.0078
MHC supertype B44. Threshold 0.750000	TEFNGKKLL	128	1.5768	-0.32699	0.0550
	SEMVNLSKM	256	1.1005	-0.29782	-0.4596
	QQPQGVLQL	280	0.7638	-0.12534	0.0078
MHC supertype B58. Threshold 0.750000	No epitopes above threshold				
MHC supertype B62. Threshold 0.750000	LQRMRTLSL	87	1.3158	-0.17898	-0.3935
	TQNNLNNTL	231	1.1772	-0.00828	0.2517

	KMRAQIKGL	51	1.1109	-0.09355	0.4497
	ILVQASQSM	266	1.1098	-0.41521	-0.0851
	IQLVNTASI	167	1.0074	0.00454	0.3822
	RVNTNVSAL	2	0.9749	-0.04724	1.0338
	QQPQGVLQL	280	0.9673	-0.12534	0.0078
	ITTASIGSM	181	0.8395	-0.05191	0.3786
	MVSSLDAAL	203	0.8390	-0.15421	0.6392
	LVQASQSML	267	0.8084	-0.51059	-0.3902
	QLVNTASIM	168	0.8024	0.01966	-0.0148

Supplementary Table S2:

All the CTL epitopes of SlpA as predicted by NetCTL 1.2

CTL Epitope prediction by NetCTL-1.2 Server					
**Mhc Super Type**	**Epitopes**	**Position**	**Prediction Score**	**Immunogenic Score**	**Antigenicity Score**
MHC supertype A1. Threshold 0.750000	FTDGEVVNY	630	3.6033	0.20872	0.6083
	SIDIDASSY	278	2.6686	-0.03673	1.5509
	YTSAENLAK	286	1.5848	0.09058	0.3438
	LVQLVNGKY	322	1.5163	-0.11205	0.0726
	SAENLAKRY	288	1.4999	-0.09974	0.6828
	STSIVDGLV	427	1.4457	0.16342	0.0608
	NLPSTGLEY	214	1.4286	-0.05123	1.0625
	DLKTYNNTY	375	1.3522	-0.02341	1.1115
	ATDTLSSDQ	674	1.2105	-0.24693	0.9880
	AIELSSKYY	398	1.1372	-0.47965	0.6886
	ITDKAVNDI	414	1.1301	-0.12534	0.4536
	ISDDAKSFL	570	1.0422	-0.2066	-1.1217
	AATTGTQGY	24	0.9743	0.04324	1.7427
	TAIELSSKY	397	0.9237	-0.2882	0.5584
	NSDDKNAIT	407	0.9124	-0.07579	0.8846
	LSSDQNVAV	678	0.8940	-0.09257	0.7414
	DPDEISEAY	299	0.7776	0.18918	0.3803
MHC supertype A2. Threshold 0.750000	YLAGGVNSI	482	1.3616	0.01654	0.3105
	AMSGLTVLA	9	1.0875	0.03450	0.0335
	QLVDALAAA	650	1.0082	0.11926	0.2580
	GLDNDKAFV	525	0.9888	-0.07929	-0.5982
	NTYSNVVTV	381	0.9776	-0.07759	0.5359
	VLVGSTSIV	423	0.9600	-0.13892	0.0026
	KVGDSTAGI	203	0.9266	-0.03842	1.6998
	GLADAMSIA	538	0.9234	-0.15404	0.5045
	TVLASAAPV	14	0.9175	-0.06196	0.2992
	FLGTSDVDI	577	0.9172	-0.04236	0.6371
	KAVNDIVLV	417	0.9079	0.18213	0.4303
	KLKDLKDYV	365	0.8072	-0.24492	-0.3634
	QLVNGKYQV	324	0.7849	-0.23391	0.3268
	AIAMSGLTV	7	0.7665	-0.27988	0.6039
MHC supertype A3. Threshold 0.750000	GIASSVINK	704	1.4898	-0.16747	0.7174
	KTLIDIATK	137	1.2176	0.3329	-0.4065
	FVAKDGSTK	639	1.1970	-0.26774	1.4372
	GTQGYTVVK	28	1.1390	0.0884	1.3806
	GTNLVQVGK	695	1.1020	-0.02746	-0.5405
	TIDFNKTLK	228	1.0015	-0.05802	0.2423
	NVAATKALK	161	0.9733	-0.08659	0.9675
	EVAPKSANK	69	0.9697	-0.33495	0.9964
	GLKDNSIGK	49	0.9225	-0.07759	1.3748
	VAVSGFVTK	248	0.9047	0.04765	0.5721
	NLVNTQLDK	90	0.8988	-0.06075	0.7009
	EIKRVMNLK	462	0.8863	-0.15496	0.5236
	VVIKANKLK	359	0.8799	-0.33027	0.3399

	VASPLASEK	435	0.8149	-0.11995	0.0941
	GVVGEVAPK	65	0.7946	0.2104	1.3072
	VIFYPEGKR	332	0.7931	0.02033	-0.6383
	VAVSKAVPK	684	0.7876	-0.29788	0.3379
	YTSAENLAK	286	0.7839	0.09058	0.3438
	KANKLKDLK	362	0.7797	-0.42066	0.5159
MHC supertype A24. Threshold 0.750000	EYAGKGTTI	221	1.5361	-0.07586	1.2510
	RYVFDPDEI	295	1.4691	0.21958	0.2827
	LYNLVNTQL	88	1.2853	-0.01407	0.2855
	TYNNTYSNV	378	1.2318	-0.11769	0.6751
	TYSNVVTVA	382	0.8766	0.07573	0.4597
	VLASAAPVF	15	0.8543	-0.06408	0.4683
	RFKESPAPI	663	0.8496	-0.11425	0.4980
	KYQVIFYPE	329	0.7581	0.23414	0.1128
MHC supertype A26. Threshold 0.750000	TAIELSSKY	397	1.7680	-0.2882	0.5584
	EISDDAKSF	569	1.4740	-0.25161	-0.1677
	TTIDFNKTL	227	1.4190	0.01411	0.0864
	DLKTYNNTY	375	1.3774	-0.02341	1.1115
	DIIGGKNSV	584	1.2679	-0.19482	1.0952
	DYVDDLKTY	371	1.1966	-0.11244	-0.5096
	SVINKMKDL	708	1.1285	-0.50765	0.9856
	NLPSTGLEY	214	1.0754	-0.05123	1.0625
	EIKRVMNLK	462	1.0722	-0.15496	0.5236
	EVLKEDDYF	622	1.0677	-0.08566	-0.2596
	FTDGEVVNY	630	1.0351	0.20872	0.6083
	ATKDTFGMV	143	1.0219	0.02632	0.6508
	YLAGGVNSI	482	1.0139	0.01654	0.3105
	DVENELKNM	493	0.9868	-0.07273	0.1360
	NIAIAMSGL	5	0.9726	-0.1004	0.5072
	ESIDSATGK	598	0.8903	-0.00619	1.6517
	ELKNMGLKV	497	0.8898	-0.35097	1.1408
	ESIDIDASS	277	0.8256	0.15236	1.4668
	EVVNYFVAK	634	0.8176	0.17119	0.2881
	YVVEMKAGA	188	0.8096	-0.20703	0.9927
	AIVTKLNSL	126	0.8000	-0.2701	0.1185
	NGKYQVIFY	327	0.7866	0.03306	0.5193
	EAYKAIVAL	305	0.7757	0.00288	-0.2211
	AATTGTQGY	24	0.7704	0.04324	1.7427
	EISEAYKAI	302	0.7602	-0.07747	-0.2584
	LVQLVNGKY	322	0.7562	-0.11205	0.0726
MHC supertype B7. Threshold 0.750000	VPKDGGTNL	690	1.3403	0.0462	0.5384
	LASEKTAPL	439	1.3368	-0.09987	-0.1832
	SPAPIILAT	667	1.1086	0.26992	0.3663
	IAMSGLTVL	8	1.0646	-0.14403	0.0799
	YPEGKRLET	335	0.9178	-0.04554	0.4349
	EAYKAIVAL	305	0.8390	0.00288	-0.2211
	APVFAATTG	20	0.8115	0.26157	0.5182
	APKSANKKA	71	0.8082	-0.51246	1.2415
	AIVTKLNSL	126	0.8046	-0.2701	0.1185
MHC supertype B8. Threshold 0.750000	EAYKAIVAL	305	1.3875	0.00288	-0.2211
	ALKVKDVAT	167	1.0994	-0.15988	1.1447
	LASEKTAPL	439	0.8617	-0.09987	-0.1832
	ENLAKRYVF	290	0.7507	-0.10451	0.2515
MHC supertype B27. Threshold 0.750000	DRYETSLAI	512	1.0085	-0.00488	0.5333
	KRLETKSAN	339	0.9737	-0.18481	1.8284
	TRLSGEDRY	506	0.8530	0.00614	0.6544
	GRFKESPAP	662	0.8501	-0.22373	0.1423
	RQATNAEVL	616	0.8271	0.19091	0.9416
MHC supertype B39. Threshold 0.750000	EAYKAIVAL	305	1.5346	0.00288	-0.2211
	RQATNAEVL	616	1.3617	0.19091	0.9416
	VKDVATFGL	170	1.3050	0.24198	0.3753

	YLAGGVNSI	482	1.1058	0.01654	0.3105
	DRYETSLAI	512	1.0214	-0.00488	0.5333
	GEDRYETSL	510	1.0053	0.08603	1.0563
	YNSDDKNAI	406	0.9949	-0.19538	0.2333
	KESPAPIIL	665	0.9906	0.15288	0.6213
	DRIETAIEL	393	0.9392	0.3894	0.3506
	ISDDAKSFL	570	0.9246	-0.2066	-1.1217
	KEDQLVDAL	647	0.9069	-0.03972	0.3141
	DKAVNDIVL	416	0.8936	0.20526	0.8956
	TTIDFNKTL	227	0.7876	0.01411	0.0864
	IAMSGLTVL	8	0.7794	-0.14403	0.0799
	DRDAAAEKL	80	0.7603	0.08	1.1257
MHC supertype B44. Threshold 0.750000	GEDRYETSL	510	1.8982	0.08603	1.0563
	KESPAPIIL	665	1.8321	0.15288	0.6213
	SEIKRVMNL	461	1.6392	-0.23652	0.0042
	SEKTAPLLL	441	1.5953	-0.01912	0.5717
	KEDQLVDAL	647	1.4746	-0.03972	0.3141
	GEDRIETAI	391	1.4513	0.33875	0.5819
	GEVVNYFVA	633	1.0910	0.16808	0.2019
	AENLAKRYV	289	0.9496	-0.13664	0.3151
	RQATNAEVL	616	0.7850	0.19091	0.9416
	VDFSVDYNL	105	0.7729	-0.07429	1.4656
MHC supertype B58. Threshold 0.750000	VLASAAPVF	15	1.4598	-0.06408	0.4683
	KAGAVEDKY	193	1.2802	0.07754	1.3773
	AAAPIAGRF	656	1.2227	0.22681	0.3841
	IASSVINKM	705	1.1396	-0.18615	0.2396
	IAMSGLTVL	8	1.0785	-0.14403	0.0799
	TAIELSSKY	397	0.9902	-0.2882	0.5584
	SSYTSAENL	284	0.9179	-0.00569	0.7926
	IATKDTFGM	142	0.9010	-0.02766	0.9089
MHC supertype B62. Threshold 0.750000	VLASAAPVF	15	1.2544	-0.06408	0.4683
	RQATNAEVL	616	1.1875	0.19091	0.9416
	YLAGGVNSI	482	1.1802	0.01654	0.3105
	TAIELSSKY	397	1.1535	-0.2882	0.5584
	LVQLVNGKY	322	1.1225	-0.11205	0.0726
	AATTGTQGY	24	1.0140	0.04324	1.7427
	NLPSTGLEY	214	0.9643	-0.05123	1.0625
	AGKGTTIDF	223	0.9015	0.16372	1.8658
	DLKTYNNTY	375	0.9011	-0.02341	1.1115
	AIELSSKYY	398	0.8885	-0.47965	0.6886
	SIGKITVSF	54	0.8773	-0.10168	0.0067
	GINTSKKVY	474	0.8571	-0.48502	0.4002
	AAAPIAGRF	656	0.8160	0.22681	0.3841
	EISDDAKSF	569	0.8080	-0.25161	-0.1677
	IGLDNDKAF	524	0.7951	-0.12584	-0.2466
	LASEKTAPL	439	0.7766	-0.09987	-0.1832

Supplementary Table S3:

All the CTL epitopes of CotE as predicted by NetCTL 1.2

CTL Epitope prediction by NetCTL-1.2 Server					
**MHC Super Type**	**Epitopes**	**Position**	**Prediction Score**	**Immunogenic Score**	**Antigenicity Score**
MHC supertype A1. Threshold 0.750000	NSSHLAWMY	78	3.5343	0.08922	0.4680
	SCDEMNFNY	297	2.4247	0.02588	1.1077
	SCDKINSSY	310	2.2773	-0.32257	1.1141
	DSDLSLPGY	572	2.1847	-0.16506	0.4628
	QVNQMINEY	466	2.0260	-0.11134	0.1882
	YINKNGYEY	624	2.0209	-0.13812	0.6808

	QSGNLFIPY	411	1.8710	0.20973	0.1626
	ITDPYNPIV	363	1.7376	0.05475	-0.6523
	YEDFYKHNY	325	1.6011	-0.05808	-0.1520
	NCEHEHHDY	245	1.4661	0.23326	0.6951
	YLCFVPDNY	209	1.3612	0.145	0.7059
	GVFSWTSTY	673	1.2102	0.00683	-0.0798
	NTELIGLSV	67	1.1892	0.07682	0.6588
	LLDAVIFAF	398	1.1808	0.33371	1.1032
	LSIFLKTQY	657	1.1245	-0.08772	0.2189
	MSDTSTVRS	119	1.1070	-0.01322	1.1240
	LSDYKGNWI	28	1.1019	-0.05066	-0.7022
	EIDQSGNLF	408	1.0957	-0.2505	-0.0939
	DINPIVAEY	378	1.0789	0.24672	0.7673
	CMDWYLCFV	205	1.0144	0.23895	0.8632
	NTAQVPYLV	636	0.9787	-0.0837	0.4056
	YTSEKNTKK	338	0.9709	-0.26228	1.9344
	QILRTILYY	135	0.9559	0.20004	-0.1314
	QTSDRDNIV	162	0.9556	0.1122	0.4116
	FTPVCTTEF	45	0.9346	0.11324	0.6215
	SVDSNSSHL	74	0.8683	-0.44202	1.4844
	AALTPTSRY	453	0.8640	-0.04818	0.0364
	RVNSCNKKY	195	0.8406	-0.53516	1.0919
	DTSTVRSVF	121	0.7834	-0.04122	0.7777
	NQILRTILY	134	0.7681	0.22482	-0.2645
MHC supertype A2. Threshold 0.750000	NLFDSDLSL	569	1.3816	- 0.18592	0.6150
	NLMSYDFTA	548	1.3311	- 0.08471	1.3517
	QLLDAVIFA	397	1.2876	0.2764	0.3951
	CMDWYLCFV	205	1.2713	0.23895	0.8632
	FIIDNNQIL	129	1.1458	0.03313	-0.5082
	LAWMYNISL	82	1.1425	- 0.09883	1.6984
	SIFLKTQYV	658	1.0989	- 0.24654	0.068
	FAMSYDDAL	649	1.0022	-0.16461	0.6789
	NILARTMSI	685	0.9948	-0.12215	0.4416
	LLLTAIRDV	505	0.9575	0.25548	-0.2705
	ITDPYNPIV	363	0.9511	0.05475	-0.6523
	YVLGNPTNV	386	0.9465	0.04274	0.6107
	WMYNISLLT	84	0.8280	-0.04968	0.8686
	YLVKDGDFA	642	0.8098	- 0.06298	-0.1204
	VLKEELEGI	700	0.8089	0.22211	0.1502
	NTAQVPYLV	636	0.7921	-0.0837	0.4056
	LLDAVIFAF	398	0.7811	0.33371	1.1032
	AVIFAFAEI	401	0.7800	0.40082	0.1476
	GMPSEKILL	593	0.7548	-0.16973	-0.0838
MHC supertype A3. Threshold 0.750000	FLNQLLALK	422	1.5420	-0.11336	0.1113
	KILLGIPFY	598	1.4308	0.20256	1.5875
	QILRTILYY	135	1.3639	0.20004	-0.1314
	GVFSWTSTY	673	1.3132	0.00683	-0.0798
	RVNSCNKKY	195	1.3020	-0.53516	1.0919
	KVHNSKQDK	284	1.2718	-0.44491	1.7887
	KIDNTCSWK	225	1.2165	-0.02246	0.9367
	GINDPEVLK	694	1.1511	0.13203	0.4362
	ILPYPKNYK	182	1.1281	-0.22874	-0.4241
	KNYDYTSEK	334	1.0257	-0.02706	1.1977
	QLLALKGEK	425	1.0121	-0.09093	0.2956
	NTNKVHNSK	281	0.9697	-0.25057	0.9979
	SSASGITSR	488	0.9574	-0.05939	1.8226
	FLKTQYVLR	660	0.9240	-0.11886	0.1619
	YTSEKNTKK	338	0.8745	-0.26228	1.9344
	QSGNLFIPY	411	0.8245	0.20973	0.1626
	CSWKKEHTK	230	0.8084	-0.21087	0.8598
	IDYFNLMSY	544	0.7808	-0.145	0.5893
	TAQVPYLVK	637	0.7639	0.00442	0.0646

	TTEFLCFAK	50	0.7541	0.21041	-1.4691
MHC supertype A24. Threshold 0.750000	IYMPNLPSL	2	1.8774	-0.19092	1.1101
	AWMYNISLL	83	1.6562	-0.08784	1.0571
	FYGRLGATI	605	1.6241	0.13988	0.5042
	YFNLMSYDF	546	1.5611	-0.33015	1.8486
	PYPRFLNQL	418	1.5499	0.0789	0.6056
	GYINSSAEI	529	1.4787	-0.18862	-0.1802
	PYPKNYKEL	184	1.4364	-0.35388	0.3433
	SYDDALSIF	652	1.4317	-0.00468	-0.2280
	IFLKTQYVL	659	1.4210	-0.27084	-0.0149
	KYSCMDWYL	202	1.4176	-0.07329	1.0323
	CFAKYYDEF	55	1.3305	-0.13416	-0.1900
	DYKGNWIVL	30	1.2517	0.30275	0.3193
	RFLNQLLAL	421	1.1576	-0.11985	-0.0141
	EYALDGIDI	473	1.1567	0.18032	0.9329
	PYLVKDGDF	641	1.1563	-0.10962	0.3676
	WFPGMPVIL	175	1.1395	-0.03834	1.1307
	VFIIDNNQI	128	1.0072	0.11949	-0.1841
	STVRSVFII	123	0.9105	0.1198	0.4749
	LFIPYPRFL	415	0.8710	0.13008	1.5582
	LTPTSRYNF	455	0.8648	-0.08382	0.5183
	FTPVCTTEF	45	0.8079	0.11324	0.6215
	NWFPGMPVI	174	0.7535	-0.0907	0.5678
	FYKHNYKNY	328	0.7514	-0.23301	-0.1985
	SCMDWYLCF	204	0.7504	0.13668	0.9475
MHC supertype A26. Threshold 0.750000	DGIDIDWEY	477	1.8545	0.46144	1.8238
	YINKNGYEY	624	1.8236	-0.13812	0.6808
	DINPIVAEY	378	1.7001	0.24672	0.7673
	PIIDYFNLM	542	1.6846	0.16018	0.5773
	QVNQMINEY	466	1.5413	-0.11134	0.1882
	FIIDNNQIL	129	1.5285	0.03313	-0.5082
	QILRTILYY	135	1.3584	0.20004	-0.1314
	KILLGIPFY	598	1.2898	0.20256	1.5875
	DVIGDDKWL	512	1.1094	0.0672	0.6141
	GVFSWTSTY	673	1.0279	0.00683	-0.0798
	EIDQSGNLF	408	1.0003	-0.2505	-0.0939
	LVKDGDFAM	643	0.9053	0.12786	-0.0994
	STYDQANIL	679	0.8546	0.01745	-0.5455
	DSDLSLPGY	572	0.8519	-0.16506	0.4628
	NQILRTILY	134	0.8475	0.22482	-0.2645
	NSSHLAWMY	78	0.8265	0.08922	0.4680
	EKILLGIPF	597	0.8254	0.15898	0.9047
	NIPEILRIV	150	0.7917	0.33775	-1.0091
	EILRIVDAL	153	0.7826	0.25852	-0.4384
	SCMDWYLCF	204	0.7811	0.13668	0.9475
	ELEGIYGQF	704	0.7521	0.15364	0.4373
MHC supertype B7. Threshold 0.750000	KPMSDTSTV	117	1.5892	-0.28227	0.2715
	YPRFLNQLL	419	1.4420	0.01347	0.5173
	LPGYSVDAM	577	1.3890	-0.07338	1.3214
	TPVCTTEFL	46	1.2551	0.18639	-0.0702
	YPGSSASGI	485	1.2163	-0.39956	1.0460
	KPSLKVIVA	433	1.0954	-0.09956	0.5670
	NPTNVDAQL	390	1.0186	0.03251	1.4221
	YPLTTGRNI	143	1.0100	0.14506	0.2755
	IPFYGRLGA	603	0.9851	0.12644	-0.1090
	TPANWFPGM	171	0.9177	0.34253	0.5256
	NPIVAEYVL	380	0.8693	0.23809	0.8519
	GPNGRKHQA	560	0.8602	-0.16098	1.9922
	LAWMYNISL	82	0.8539	-0.09883	1.6984
	FARQVNQMI	463	0.8389	-0.26601	-0.0219
	APIIDYFNL	541	0.8327	0.29026	0.7179
	RPQDRENFT	496	0.8082	0.19231	0.4723

	APDFKANTT	14	0.7784	-0.03095	1.4259
	TPTSRYNFA	456	0.7695	-0.04401	0.9264
MHC supertype B8. Threshold 0.750000	EFKKRNTEL	62	1.9434	-0.15143	0.9330
	YPRFLNQLL	419	1.8264	0.01347	0.5173
	FARQVNQMI	463	1.1886	-0.26515	-0.0219
	NGRKHQANL	562	1.0444	-0.2485	1.5481
	IFLKTQYVL	659	0.9598	-0.27084	-0.0149
	FKKRNTELI	63	0.9444	0.09034	-0.0556
	NPIVAEYVL	380	0.9091	0.23809	0.8519
	NILARTMSI	685	0.7841	-0.12215	0.4416
	FAMSYDDAL	649	0.7717	-0.16461	0.6789
	EILRIVDAL	153	0.7675	0.25852	-0.4384
MHC supertype B27. Threshold 0.750000	GRLGATITR	607	1.8691	0.24014	0.7534
	MRIAKLYGM	106	1.4654	-0.12119	-0.0131
	YRFDNTAQV	632	1.4400	0.0559	0.6328
	KRNTELIGL	65	1.4393	0.25614	0.2879
	LRTILYYPL	137	1.3758	0.12264	0.9102
	SRYNFARQV	459	1.1253	0.11912	0.0878
	LRNCLGGVF	667	1.1153	0.01747	-0.2235
	VRNLENAGM	586	0.9680	0.13097	-0.1121
	NRVNSCNKK	194	0.9159	-0.33642	0.9913
	YKGNWIVLF	31	0.8875	0.37383	0.2922
	VRPQITDPY	359	0.8817	0.05822	0.9772
	GRKHQANLF	563	0.8547	-0.12536	0.4805
	KKIAMKTLK	345	0.8394	-0.26515	0.5793
	GRNIPEILR	148	0.8090	0.32111	-0.9083
	SRPQDRENF	495	0.7734	0.03088	0.3493
	NQILRTILY	134	0.7616	0.22482	-0.2645
MHC supertype B39. Threshold 0.750000	HHDYLNKAL	250	2.4576	-0.17255	-0.0791
	PQDRENFTL	497	1.8368	0.27217	0.2694
	TQYVLRNCL	663	1.6268	0.0413	0.2631
	NQMINEYAL	468	1.5546	0.18461	0.6284
	STYDQANIL	679	1.5072	0.01745	-0.5455
	DRENFTLLL	499	1.3227	0.16069	0.1061
	FIIDNNQIL	129	1.2344	0.03313	-0.5082
	YKGNWIVLF	31	1.1759	0.37383	0.2922
	YRFDNTAQV	632	1.1366	0.0559	0.6328
	LRTILYYPL	137	1.0827	0.12264	0.9102
	YDDALSIFL	653	1.0776	0.06076	0.0855
	IYMPNLPSL	2	1.0115	-0.19092	1.1101
	SHLAWMYNI	80	0.8748	0.07927	0.8317
	IFLKTQYVL	659	0.8545	-0.27084	-0.0149
	SYDDALSIF	652	0.7843	-0.00468	-0.2280
	DYKGNWIVL	30	0.7586	0.30275	0.3193
	FKDKSCDEM	293	0.7552	-0.34443	1.2388
MHC supertype B44. Threshold 0.750000	AEGFSDAAL	447	1.6588	0.04446	0.1913
	AEIDQSGNL	407	1.5757	-0.17819	0.4780
	CEHEHHDYL	246	1.5492	0.18976	0.1689
	KEDSSYEDF	320	1.2538	-0.22639	0.4554
	YEDFYKHNY	325	1.1051	-0.05808	-0.1520
	GEKPSLKVI	431	0.9314	-0.41058	1.4009
	AEIDKIAPI	535	0.8847	0.00734	-0.0697
	TEFLCFAKY	51	0.8436	-0.00844	-1.1705
	YEYRFDNTA	630	0.8289	0.20298	1.5906
	YDDALSIFL	653	0.8152	0.06076	0.0855
	VENANCPDI	371	0.7672	-0.01618	0.8590
	AQLLDAVIF	396	0.7657	0.15627	0.5018
	PQDRENFTL	497	0.7608	0.27217	0.2694
MHC supertype B58. Threshold 0.750000	KVIVAIGGW	437	1.8473	0.29652	-1.0937
	DSNSSHLAW	76	1.6964	-0.28572	0.8410
	KIAPIIDYF	539	1.5388	0.27298	0.5142

	KKIDNTCSW	224	1.4131	-0.0464	0.4854
	RLSDYKGNW	27	1.3700	-0.21316	0.2939
	LSIFLKTQY	657	1.3473	-0.08772	0.2189
	NSSHLAWMY	78	1.2483	0.08922	0.4680
	LLDAVIFAF	398	1.2093	0.33371	1.1032
	NCLGGVFSW	669	0.8573	0.1045	0.0896
	ALDGIDIDW	475	0.7763	0.31706	1.6523
	QVNQMINEY	466	0.7711	-0.11134	0.1882
	MSYDDALSI	651	0.7661	-0.02647	0.4449
	QILRTILYY	135	0.7630	0.20004	-0.1314
	LLTGVEIPF	90	0.7610	0.28699	0.3229
MHC supertype B62. Threshold 0.750000	GVFSWTSTY	673	1.4088	0.00683	-0.0798
	NQILRTILY	134	1.3629	0.22482	-0.2645
	QVNQMINEY	466	1.3561	-0.11134	0.1882
	YINKNGYEY	624	1.2912	-0.13812	0.6808
	AQLLDAVIF	396	1.2715	0.15627	0.5018
	LLTGVEIPF	90	1.2296	0.28699	0.3229
	QILRTILYY	135	1.1960	0.20004	-0.1314
	LVKDGDFAM	643	1.1429	0.12786	-0.0994
	LLDAVIFAF	398	1.1167	0.33371	1.1032
	QSGNLFIPY	411	1.1074	0.20973	0.1626
	RVNSCNKKY	195	1.0933	-0.53516	1.0919
	LGATITRTY	609	1.0220	0.28426	0.5934
	KILLGIPFY	598	1.0085	0.20256	1.5875
	LSIFLKTQY	657	0.9578	-0.08772	0.2189
	TQYVLRNCL	663	0.9492	0.0413	0.2631
	PGMPVILPY	177	0.9449	0.08148	1.0662
	NLFDSDLSL	569	0.9432	-0.18592	0.6150
	KIAPIIDYF	539	0.9429	0.27298	0.5142
	NLFIPYPRF	414	0.9185	0.17852	1.4534
	YFNLMSYDF	546	0.8994	-0.33015	1.8486
	FTPVCTTEF	45	0.8836	0.11324	0.6215
	SCMDWYLCF	204	0.8730	0.13668	0.9475
	VLFSHPGDF	37	0.8589	-0.06585	-0.8060
	YLCFVPDNY	209	0.8514	0.145	0.7059
	AALTPTSRY	453	0.8343	-0.04818	0.0364
	FDNTAQVPY	634	0.8329	-0.00562	-0.3244
	NQMINEYAL	468	0.8014	0.18461	0.6284
	FIIDNNQIL	129	0.7881	0.03313	-0.5082
	VAGTGDRGY	522	0.7553	0.16742	1.3790

Supplementary Table S4:

All the HTL epitopes of FliC protein as predicted by NetMHC II pan 3.2 where Sb indicates Strong binder

Epitopes (Sb)	HLA Alleles II	Core Sequences	Score	Immunogenicity	Antigenicity
AALKSLNSSRAKLGA (209)	DRB1*01:01	LKSLNSSRA	0.90	-0.65625	0.8274
	DRB1*04:01	LKSLNSSRA	1.20		
LDAALKSLNSSRAKL (207)	DRB1*01:01	LKSLNSSRA	0.90	-0.71339	0.7629
	DRB1*04:01	LKSLNSSRA	0.80		
	DRB1*04:05	LKSLNSSRA	1.40		
DAALKSLNSSRAKLGV (208)	DRB1*01:01	LKSLNSSRA	1.10	-0.81292	0.9414
	DRB1*04:01	LKSLNSSRA	1.10		
ALKSLNSSRAKLGAQ (210)	DRB1*01:01	LKSLNSSRA	1.70	-0.82506	1.0020
EIKIQLVNTASIMAS (164)	DRB1*01:01	IQLVNTASI	1.70	-0.11104	0.8191
	DRB1*04:01	IQLVNTASI	0.90		
	DRB1*04:05	IQLVNTASI	1.40		
	DRB1*07:01	IQLVNTASI	1.30		
	DRB1*08:02	IQLVNTASI	0.50		

	DRB1*12:01	IQLVNTASI	1.20		
	DRB1*13:02	IQLVNTASI	0.30		
	DRB3*02:02	QLVNTASIM	0.90		
	HLA-DQA1*01:02-DQB1*06:02	VNTASIMAS	0.60		
GNILQRMRTLSLQSA (84)	DRB1*01:01	LQRMRTLSL	1.90	-0.48992	-0.0850
	DRB1*04:01	LQRMRTLSL	1.50		
	DRB1*04:05	LQRMRTLSL	1.10		
	DRB1*08:02	LQRMRTLSL	1.10		
	DRB1*11:01	LQRMRTLSL	1.40		
	DRB1*12:01	LQRMRTLSL	0.25		
	DRB1*15:01	LQRMRTLSL	1.50		
	DRB4*01:01	LQRMRTLSL	0.03		
IKIQLVNTASIMASA (165)	DRB1*01:01	IQLVNTASI	1.90	-0.2348	0.6573
	DRB1*04:01	IQLVNTASI	1.20		
	DRB1*04:05	IQLVNTASI	1.90		
	DRB1*07:01	IQLVNTASI	1.40		
	DRB1*08:02	IQLVNTASI	0.50		
	DRB1*12:01	IQLVNTASI	1.50		
	DRB1*13:02	IQLVNTASI	0.40		
	DRB4*01:01	IQLVNTASI	0.80		
	HLA-DQA1*05:01-DQB1*03:01	VNTASIMAS	1.50		
	HLA-DQA1*01:02-DQB1*06:02	VNTASIMAS	0.01		
	DRB1*01:01	IQLVNTASI	1.90	-0.20401	0.7011
	DRB1*04:01	IQLVNTASI	0.90		
	DRB1*04:05	IQLVNTASI	1.30		
	DRB1*07:01	IQLVNTASI	1.40		
	DRB1*12:01	IQLVNTASI	1.30		
	DRB1*13:02	IQLVNTASI	0.40		
	DRB3*02:02	QLVNTASIM	1.30		
	DRB4*01:01	IKIQLVNTA	0.10		
NNEIKIQLVNTASIM (162)	DRB1*04:01	IKIQLVNTA	1.10	0.00909	0.7263
	DRB1*04:05	IKIQLVNTA	1.90		
	DRB1*08:02	IKIQLVNTA	0.90		
	DRB1*12:01	IQLVNTASI	1.80		
	DRB1*13:02	IQLVNTASI	0.50		
	DRB4*01:01	IKIQLVNTA	0.10		
SLDAALKSLNSSRAK (206)	DRB1*04:01	LKSLNSSRA	1.70	-0.56462	0.9694
TGNILQRMRTLSLQS (83)	DRB1*04:01	LQRMRTLSL	1.90	-0.26775	-0.0589
	DRB1*04:05	LQRMRTLSL	1.40		
	DRB1*08:02	LQRMRTLSL	1.60		
	DRB1*11:01	LQRMRTLSL	1.50		
	DRB1*12:01	LQRMRTLSL	0.30		
	DRB1*15:01	LQRMRTLSL	1.70		
	DRB4*01:01	LQRMRTLSL	0.04		
NNNEIKIQLVNTASI (161)	DRB1*04:01	IKIQLVNTA	2.00	0.03105	0.9979
	DRB1*08:02	IKIQLVNTA	1.90		
	DRB1*13:02	IQLVNTASI	1.50		
	DRB4*01:01	IKIQLVNTA	0.20		
ETGNILQRMRTLSLQ (82)	DRB1*04:05	LQRMRTLSL	1.90	-0.17845	-0.0162
	DRB1*11:01	LQRMRTLSL	1.80		
	DRB1*12:01	LQRMRTLSL	0.50		
	DRB4*01:01	LQRMRTLSL	0.08		
KIQLVNTASIMASAG (166)	DRB1*08:02	LVNTASIMA	0.90	-0.22139	0.3215
	DRB1*13:02	LVNTASIMA	0.70		
	DRB3*02:02	QLVNTASIM	1.20		
	HLA-	VNTASIMAS	0.90		

	DQA1*05:01-DQB1*03:01				
	HLA-DQA1*01:02-DQB1*06:02	VNTASIMAS	0.01		
NILQRMRTLSLQSAN (85)	DRB1*08:02	LQRMRTLSL	1.20	-0.56106	0.0209
	DRB1*12:01	LQRMRTLSL	0.50		
	DRB4*01:01	LQRMRTLSL	0.05		
	HLA-DQA1*01:02-DQB1*06:02	MRTLSLQSA	2.00		
ILQRMRTLSLQSANE (86)	DRB1*08:02	LQRMRTLSL	2.0	-0.49031	0.0155
	DRB1*12:01	LQRMRTLSL	1.10		
	DRB4*01:01	LQRMRTLSL	0.12		
MRVNTNVSALIANNQ (1)	DRB1*13:02	VNTNVSALI	0.50	0.10198	0.4654
	DRB3*02:02	VNTNVSALI	0.90		
	HLA-DQA1*01:02-DQB1*06:02	NTNVSALIA	0.60		
RVNTNVSALIANNQM (2)	DRB1*13:02	VNTNVSALI	1.20	0.02553	0.4418
	HLA-DQA1*01:02-DQB1*06:02	NTNVSALIA	0.60		
EETGNILQRMRTLSL (81)	DRB4*01:01	LQRMRTLSL	0.40	-0.09328	-0.1517
EIKIQLVNTASIMAS (164)	DRB4*01:01	IKIQLVNTA	0.17	-0.11104	0.8191
	HLA-DQA1*01:02-DQB1*06:02	VNTASIMAS	0.6		
LQRMRTLSLQSANEI (87)	DRB4*01:01	MRTLSLQSA	0.50	-0.43843	0.0734
	HLA-DQA1*01:02-DQB1*06:02	MRTLSLQSA	1.40		
NNNNEIKIQLVNTAS (160)	DRB4*01:01	IKIQLVNTA	0.60	0.10422	0.9595
KMNILVQASQSMLAQ (263)	DRB4*01:01	ILVQASQSM	0.80	-0.50028	0.1876
	HLA-DQA1*01:02-DQB1*06:02	VQASQSMLA	1.20		
SKMNILVQASQSMLA (262)	DRB4*01:01	ILVQASQSM	1.10	-0.56352	0.0626
	HLA-DQA1*01:02-DQB1*06:02	NILVQASQS	1.10		
MNILVQASQSMLAQA (264)	DRB4*01:01	ILVQASQSM	1.10	-0.65356	0.1867
	HLA-DQA1*01:02-DQB1*06:02	VQASQSMLA	0.60		
LSKMNILVQASQSML (261)	DRB4*01:01	ILVQASQSM	1.60	-0.68106	-0.0830
	HLA-DQA1*01:02-DQB1*06:02	NILVQASQS	1.70		
NILVQASQSMLAQAN (265)	DRB4*01:01	ILVQASQSM	1.90	-0.69163	0.1430
	HLA-DQA1*01:02-DQB1*06:02	VQASQSMLA	0.50		
ASIMASAGITTASIG (173)	HLA-DQA1*05:01-DQB1*03:01	ASAGITTAS	0.12	-0.00523	0.5784
	HLA-DQA1*01:02-DQB1*06:02	MASAGITTA	0.90		
TASIMASAGITTASI (172)	HLA-DQA1*05:01-DQB1*03:01	ASAGITTAS	0.12	-0.00238	0.5455
	HLA-	MASAGITTA	0.70		

	DQA1*01:02-DQB1*06:02				
SIMASAGITTASIGS (174)	HLA-DQA1*05:01-DQB1*03:01	ASAGITTAS	0.15	0.11206	0.6460
	HLA-DQA1*01:02-DQB1*06:02	MASAGITTA	1.00		
NTASIMASAGITTAS (171)	HLA-DQA1*05:01-DQB1*03:01	MASAGITTA	0.20	-0.02531	0.5184
	HLA-DQA1*01:02-DQB1*06:02	MASAGITTA	0.20		
IMASAGITTASIGSM (175)	HLA-DQA1*05:01-DQB1*03:01	ASAGITTAS	0.25	0.05675	0.5090
	HLA-DQA1*01:02-DQB1*06:02	MASAGITTA	1.20		
VNTASIMASAGITTA (170)	HLA-DQA1*05:01-DQB1*03:01	MASAGITTA	0.25	-0.02171	0.5887
	HLA-DQA1*01:02-DQB1*06:02	NTASIMASA	0.02		
LVNTASIMASAGITT (169)	HLA-DQA1*05:01-DQB1*03:01	VNTASIMAS	0.40	-0.04544	0.3233
	HLA-DQA1*01:02-DQB1*06:02	NTASIMASA	0.01		
MASAGITTASIGSMK (176)	HLA-DQA1*05:01-DQB1*03:01	ASAGITTAS	0.40	0.02015	0.6946
QLVNTASIMASAGIT (168)	HLA-DQA1*05:01-DQB1*03:01	VNTASIMAS	0.50	-0.09952	0.3266
	HLA-DQA1*01:02-DQB1*06:02	VNTASIMAS	0.01		
IQLVNTASIMASAGI (167)	HLA-DQA1*05:01-DQB1*03:01	VNTASIMAS	0.60	-0.15977	0.2463
	HLA-DQA1*01:02-DQB1*06:02	VNTASIMAS	0.01		
ASAGITTASIGSMKA (177)	HLA-DQA1*05:01-DQB1*03:01	ASAGITTAS	0.90	-0.13833	0.8134
SAGITTASIGSMKAG (178)	HLA-DQA1*05:01-DQB1*03:01	ITTASIGSM	1.90	-0.22542	0.9805
ILVQASQSMLAQANQ (266)	HLA-DQA1*01:02-DQB1*06:02	VQASQSMLA	0.60	-0.72356	0.0231
LVQASQSMLAQANQQ (267)	HLA-DQA1*01:02-DQB1*06:02	ASQSMLAQA	0.60	-0.72604	0.1068
VNTNVSALIANNQMG (3)	HLA-DQA1*01:02-DQB1*06:02	NVSALIANN	0.80	-0.13256	0.5622
VQASQSMLAQANQQP (268)	HLA-DQA1*01:02-	ASQSMLAQA	1.10	-0.8176	0.3584

	DQB1*06:02				
NTNVSALIANNQMGR (4)	HLA-DQA1*01:02-DQB1*06:02	NVSALIANN	1.50	-0.1767	0.5563
NLSKMNILVQASQSM (260)	HLA-DQA1*01:02-DQB1*06:02	KMNILVQAS	1.80	-0.71385	0.1443
QRMRTLSLQSANEIN (88)	HLA-DQA1*01:02-DQB1*06:02	MRTLSLQSA	1.90	-0.22945	0.5177
ILQRMRTLSLQSANE (86)	HLA-DQA1*01:02-DQB1*06:02	MRTLSLQSA	1.90	-0.49031	0.0155
VNLSKMNILVQASQS (259)	HLA-DQA1*01:02-DQB1*06:02	KMNILVQAS	2.00	-0.68164	0.3258

Supplementary Table S5:

All the HTL epitopes of SlpA as predicted by NetMHC II pan 3.2 where Sb indicates Strong binder

**Epitopes(Sb)**	**HLA Alleles II**	**Core Sequences**	**Score**	**Immunogenicity**	Antigenicity
SEAYKAIVALQNDGI (304)	DRB1*01:01	YKAIVALQN	0.50	-0.04619	-0.1185
	DRB1*04:01		0.20		
	DRB1*04:05		0.25		
	DRB1*08:02		0.50		
	DRB1*09:01		1.10		
	DPA1*02:01-DPB1*14:01		0.09		
EAYKAIVALQNDGIE (305)	DRB1*01:01	YKAIVALQN	0.70	0.02514	0.0340
	DRB1*04:01		0.50		
	DRB1*04:05		0.40		
	DRB1*08:02		0.70		
	DRB1*09:01		1.70		
	DPA1*02:01-DPB1*14:01		0.15		
ISEAYKAIVALQNDG (303)	DRB1*01:01	YKAIVALQN	0.70	-0.00807	-0.3044
	DRB1*04:01		0.25		
	DRB1*04:05		0.30		
	DRB1*08:02		0.60		
	DRB1*09:01		1.40		
	DPA1*02:01-DPB1*14:01		0.12		
	DQA1*01:02-DQB1*06:02		1.70		
	DQA1*01:02-DQB1*06:02		1.70		
EISEAYKAIVALQND (302)	DRB1*01:01	YKAIVALQN	0.80	0.00557	-0.2088
	DRB1*04:01		0.25		
	DRB1*04:05		0.25		
	DRB1*08:02		1.00		
	DRB1*09:01		1.20		
	DPA1*02:01-DPB1*14:01		0.15		
	DQA1*01:02-DQB1*06:02		1.20		
	DQA1*01:02-DQB1*06:02		1.20		
KKNIAIAMSGLTVLA (3)	DRB1*01:01	IAIAMSGLT	0.90	0.09262	0.1193
	DRB1*07:01		1.80		
	DRB1*08:02		1.80		
	DRB1*09:01		1.00		
	DRB1*12:01		1.60		
	DRB1*15:01		1.40		

	DPA1*02:01-DPB1*14:01		1.20		
	DQA1*01:02-DQB1*06:02		1.40		
AYKAIVALQNDGIES (306)	DRB1*01:01	YKAIVALQN	1.20	0.27169	0.1951
	DRB1*04:01		0.70		
	DRB1*04:05		0.70		
	DRB1*08:02		1.10		
	DPA1*02:01-DPB1*14:01		0.30		
SKKVYLAGGVNSISK (478)	DRB1*01:01	YLAGGVNSI	1.20	-0.04493	0.0498
	DRB1*09:01		1.20		
	DQA1*05:01-DQB1*03:01		1.30		
NKKNIAIAMSGLTVL (2)	DRB1*01:01	IAIAMSGLT	1.30	0.00623	0.1471
	DRB1*09:01		1.20		
	DPA1*02:01-DPB1*14:01		1.00		
KNIAIAMSGLTVLAS (4)	DRB1*01:01	IAIAMSGLT	1.40	0.03053	0.0776
	DRB1*09:01		1.50		
	DQA1*05:01-DQB1*03:01		1.90		
	DQA1*01:02-DQB1*06:02		0.25		
DEISEAYKAIVALQN (301)	DRB1*01:01	YKAIVALQN	1.50	-0.03358	-0.2523
	DRB1*04:01		0.60		
	DRB1*04:05		0.50		
	DRB1*08:02		1.70		
	DRB1*09:01		1.30		
	DPA1*02:01-DPB1*14:01		0.25		
	DQA1*01:02-DQB1*06:02		1.20		
SGLTVLASAAPVFAA (11)	DRB1*01:01	TVLASAAPV	1.50	0.16778	0.3077
	DRB1*09:01		1.20		
	DQA1*05:01-DQB1*03:01		0.10		
	DQA1*03:01-DQB1*03:02		1.60		
	DQA1*04:01-DQB1*04:02		1.00		
	DQA1*01:02-DQB1*06:02		0.06		
GLTVLASAAPVFAAT (12)	DRB1*01:01	TVLASAAPV	1.50	0.19202	0.2910
	DRB1*09:01		1.30		
	DPA1*02:01-DPB1*14:01		2.00		
	DQA1*05:01-DQB1*03:01		0.06		
	DQA1*03:01-DQB1*03:02		1.90		
	DQA1*04:01-DQB1*04:02		1.20		
	DQA1*01:02-DQB1*06:02		0.04		
KKVYLAGGVNSISKD (479)	DRB1*01:01	YLAGGVNSI	1.60	-0.16456	0.0904
	DRB1*09:01		1.80		
ATPIVVVDGKAKEIS (557)	DRB1*03:01	VVVDGKAKE	0.50	0.09288	0.9390
TPIVVVDGKAKEISD (558)	DRB1*03:01	VVVDGKAKE	0.60	-0.05225	0.8437
DATPIVVVDGKAKEI (556)	DRB1*03:01	VVVDGKAKE	0.70	0.00957	0.8429
PIVVVDGKAKEISDD (559)	DRB1*03:01	VVVDGKAKE	0.80	-0.13604	1.0012
IVVVDGKAKEISDDA (560)	DRB1*03:01	VVVDGKAKE	1.30	-0.16931	0.8012
ASSYTSAENLAKRYV (283)	DRB1*04:01	YTSAENLAK	2.00	-0.1856	0.5662
AEKLYNLVNTQLDKL (85)	DRB1*04:05	YNLVNTQLD	0.80	-0.26068	0.1690
EKLYNLVNTQLDKLG (86)	DRB1*04:05	YNLVNTQLD	0.90	-0.24392	0.2636
AAEKLYNLVNTQLDK (84)	DRB1*04:05	YNLVNTQLD	1.30	-0.24974	0.2274
KLYNLVNTQLDKLGD (87)	DRB1*04:05	YNLVNTQLD	1.60	-0.24137	0.4727
SEGKNVAATKALKVK (157)	DRB1*07:01	VAATKALKV	0.25	-0.39832	1.5186
	DRB1*09:01		0.70		

	DPA1*02:01-DPB1*14:01		0.90		
GKNVAATKALKVKDV (159)	DRB1*07:01	VAATKALKV	0.25	-0.36944	1.1795
	DRB1*09:01		0.80		
	DPA1*02:01-DPB1*14:01		0.90		
KNVAATKALKVKDVA (160)	DRB1*07:01	VAATKALKV	0.40	-0.38399	0.9365
	DRB1*09:01		1.10		
	DPA1*02:01-DPB1*14:01		1.20		
EGKNVAATKALKVKD (158)	DRB1*07:01	VAATKALKV	0.40	-0.39917	1.3823
	DRB1*09:01		1.10		
	DPA1*02:01-DPB1*14:01		1.20		
IAGRFKESPAPIILA (660)	DRB1*07:01	FKESPAPII	0.60	0.1511	0.4282
	DRB1*09:01		0.50		
	DRB3*02:02		1.80		
	DPA1*02:01-DPB1*14:01		1.40		
DSEGKNVAATKALKV (156)	DRB1*07:01	VAATKALKV	0.60	-0.30403	1.6653
	DRB1*09:01		1.80		
AGRFKESPAPIILAT (661)	DRB1*07:01	FKESPAPII	0.60	0.14588	0.2349
	DRB1*09:01		0.50		
	DRB3*02:02		1.70		
	DPA1*02:01-DPB1*14:01		1.30		
GRFKESPAPIILATD (662)	DRB1*07:01	FKESPAPII	0.90	0.07836	0.1879
	DRB1*09:01		0.70		
	DPA1*02:01-DPB1*14:01		1.70		
PIAGRFKESPAPIIL (659)	DRB1*07:01	FKESPAPII	1.10	0.14454	0.4524
	DRB1*09:01		0.90		
NVAATKALKVKDVAT (161)	DRB1*07:01	VAATKALKV	1.40	-0.39405	1.1342
RFKESPAPIILATDT (663)	DRB1*07:01	FKESPAPII	1.80	0.2171	0.4166
	DRB1*09:01		1.60		
KSEIKRVMNLKSDTG (460)	DRB1*08:02	IKRVMNLKS	1.40	-0.46541	0.7432
	DRB4*01:01		1.70		
VKSEIKRVMNLKSDT (459)	DRB1*08:02	IKRVMNLKS	1.40	-0.46051	0.6619
	DRB4*01:01		1.50		
TSKKVYLAGGVNSIS (477)	DRB1*09:01	YLAGGVNSI	1.60	-0.18485	0.3085
	DQA1*05:01-DQB1*03:01		1.60		
LTVLASAAPVFAATT (13)	DRB1*09:01	LASAAPVFA	1.80	0.19137	0.2554
	DQA1*05:01-DQB1*03:01		0.05		
	DQA1*04:01-DQB1*04:02		1.50		
	DQA1*01:02-DQB1*06:02		0.05		
MNKKNIAIAMSGLTV (1)	DRB1*09:01	IAIAMSGLT	2	-0.24478	0.3858
SNLVQLVNGKYQVIF (320)	DRB1*12:01	LVQLVNGKY	1.30	-0.2288	-0.0702
ESNLVQLVNGKYQVI (319)	DRB1*12:01	LVQLVNGKY	1.40	-0.31688	0.0519
NLVQLVNGKYQVIFY (321)	DRB1*12:01	LVQLVNGKY	2.00	-0.15388	0.0646
IESNLVQLVNGKYQV (318)	DRB1*12:01	LVQLVNGKY	2.00	-0.36131	0.2899
KGTTIDFNKTLKVDV (225)	DRB1*13:02	IDFNKTLKV	1.80	-0.02764	0.6933
LAKRYVFDPDEISEA (292)	DRB3*01:01	YVFDPDEIS	1.60	0.30674	0.1275
GTTIDFNKTLKVDVT (226)	DRB1*13:02	IDFNKTLKV	1.90	-0.03548	0.7535
IADEIGLDNDKAFVV (520)	DRB3*01:01	IGLDNDKAF	1.60	0.2464	0.0676
YVDFSVDYNLENKII (104)	DRB3*01:01	FSVDYNLEN	0.90	-0.00783	0.7761
AKRYVFDPDEISEAY (293)	DRB3*01:01	YVFDPDEIS	1.20	0.37841	0.1043
DYVDFSVDYNLENKI (103)	DRB3*01:01	FSVDYNLEN	1.10	-0.00749	0.6685
KRYVFDPDEISEAYK (294)	DRB3*01:01	YVFDPDEIS	1.50	0.37785	-0.0054
VDFSVDYNLENKIIT (105)	DRB3*01:01	FSVDYNLEN	1.40	-0.00909	0.8860
GDYVDFSVDYNLENK (102)	DRB3*01:01	FSVDYNLEN	1.70	0.13702	0.7298
ADEIGLDNDKAFVVG (521)	DRB3*01:01	IGLDNDKAF	1.80	0.22278	0.1384
RYETSLAIADEIGLD (513)	DQA10501-DQB10201	TSLAIADEI	0.25	0.37196	0.7434
	DQA1*03:01-DQB1*03:02		0.07		

	DQA1*04:01-DQB1*04:02		0.07		
DRYETSLAIADEIGL (512)	DQA10501-DQB10201	TSLAIADEI	0.25	0.41922	0.4522
	DQA1*03:01-DQB1*03:02		0.05		
	DQA1*04:01-DQB1*04:02		0.05		
GEDRYETSLAIADEI (510)	DQA10501-DQB10201	TSLAIADEI	0.40	0.30083	0.5738
	DQA1*03:01-DQB1*03:02		0.04		
	DQA1*04:01-DQB1*04:02		0.06		
YETSLAIADEIGLDN (514)	DQA1*05:01-DQB1*02:01	TSLAIADEI	0.50	0.32533	0.5443
	DQA1*03:01-DQB1*03:02		0.15		
	DQA1*04:01-DQB1*04:02		0.20		
EDRYETSLAIADEIG (511)	DQA1*05:01-DQB1*02:01	TSLAIADEI	0.50	0.36542	0.5417
	DQA1*03:01-DQB1*03:02		0.07		
	DQA1*04:01-DQB1*04:02		0.09		
ETSLAIADEIGLDND (515)	DQA10501-DQB10201	TSLAIADEI	1.30	0.42714	0.6268
	DQA1*03:01-DQB1*03:02		1.00		
	DQA1*04:01-DQB1*04:02		1.40		
VTGGSTPSAVAVSGF (239)	DQA1*05:01-DQB1*03:01	VTGGSTPSA	0.25	-0.21466	1.0751
TVLASAAPVFAATTG (14)	DQA1*05:01-DQB1*03:01	ALAAAPIAG	0.09	0.22464	0.3862
	DQA1*01:02-DQB1*06:02		0.08		
VDVTGGSTPSAVAVS (237)	DQA1*05:01-DQB1*03:01	VTGGSTPSA	0.15	-0.04264	1.4615
KVDVTGGSTPSAVAV (236)	DQA1*05:01-DQB1*03:01	VTGGSTPSA	0.17	-0.04813	1.5436
DVTGGSTPSAVAVSG (238)	DQA1*05:01-DQB1*03:01	VTGGSTPSA	0.20	-0.16219	1.5837
VLASAAPVFAATTGT (15)	DQA1*05:01-DQB1*03:01	ALAAAPIAG	0.20	0.23113	0.5622
	DQA1*01:02-DQB1*06:02		0.20		
LKVDVTGGSTPSAVA (235)	DQA1*05:01-DQB1*03:01	VTGGSTPSA	0.30	-0.05297	1.6816
LVDALAAAPIAGRFK (651)	DQA1*05:01-DQB1*03:01	ALAAAPIAG	0.40	0.45095	0.3253
	DQA1*01:02-DQB1*06:02		0.70		
QLVDALAAAPIAGRF (650)	DQA1*05:01-DQB1*03:01	ALAAAPIAG	0.40	0.39179	0.4022
	DQA1*01:02-DQB1*06:02		0.70		
LASAAPVFAATTGTQ (16)	DQA1*05:01-DQB1*03:01	ALAAAPIAG	0.40	0.36899	0.7669
	DQA1*01:02-DQB1*06:02		0.90		
MSGLTVLASAAPVFA (10)	DQA1*05:01-DQB1*03:01	TVLASAAPV	0.40	0.11304	0.2691
	DQA1*04:01-DQB1*04:02		1.70		
	DQA1*01:02-DQB1*06:02		0.09		
VDALAAAPIAGRFKE (652)	DQA1*05:01-DQB1*03:01	ALAAAPIAG	0.50	0.32726	0.3518
	DQA1*01:02-DQB1*06:02		1.10		
TGGSTPSAVAVSGFV (240)	DQA1*05:01-	STPSAVAVS	0.60	-0.1917	1.0400

	DQB1*03:01				
DQLVDALAAAPIAGR (649)	DQA1*05:01-DQB1*03:01	ALAAAPIAG	0.60	0.36844	0.4791
	DQA1*01:02-DQB1*06:02		1.20		
DALAAAPIAGRFKES (653)	DQA1*05:01-DQB1*03:01	ALAAAPIAG	0.70	0.33897	0.4174
AMSGLTVLASAAPVF (9)	DQA1*05:01-DQB1*03:01	TVLASAAPV	0.70	0.00349	0.2768
	DQA1*01:02-DQB1*06:02		0.04		
TLKVDVTGGSTPSAV (234)	DQA1*05:01-DQB1*03:01	VTGGSTPSA	0.80	-0.11336	1.7253
IAMSGLTVLASAAPV (8)	DQA1*05:01-DQB1*03:01	TVLASAAPV	0.90	-0.1937	0.2505
	DQA1*01:02-DQB1*06:02		0.03		
GGSTPSAVAVSGFVT (241)	DQA1*05:01-DQB1*03:01	STPSAVAVS	1.00	-0.03622	0.8122
EDQLVDALAAAPIAG (648)	DQA1*05:01-DQB1*03:01	ALAAAPIAG	1.20	0.27938	0.4662
	DQA1*04:01-DQB1*04:02		1.80		
	DQA1*01:02-DQB1*06:02		1.80		
ASAAPVFAATTGTQG (17)	DQA1*05:01-DQB1*03:01	ASAAPVFAA	1.20	0.34425	1.0950
IAIAMSGLTVLASAA (6)	DQA1*05:01-DQB1*03:01	AMSGLTVLA	1.20	-0.24006	0.2817
	DQA1*01:02-DQB1*06:02		0.04		
TNLVQVGKGIASSVI (696)	DQA1*05:01-DQB1*03:01	VGKGIASSV	1.30	-0.28219	-0.0655
NLVQVGKGIASSVIN (697)	DQA1*05:01-DQB1*03:01	VGKGIASSV	1.30	-0.24349	-0.0134
ALAAAPIAGRFKESP (654)	DQA1*05:01-DQB1*03:01	ALAAAPIAG	1.30	0.22931	0.4505
AIAMSGLTVLASAAP (7)	DQA1*05:01-DQB1*03:01	AMSGLTVLA	1.40	-0.29745	0.2499
	DQA1*01:02-DQB1*06:02		0.03		
GSTPSAVAVSGFVTK (242)	DQA1*05:01-DQB1*03:01	STPSAVAVS	1.40	0.03656	0.7348
LVQVGKGIASSVINK (698)	DQA1*05:01-DQB1*03:01	VGKGIASSV	1.40	-0.14716	-0.0382
KVYLAGGVNSISKDV (480)	DQA1*05:01-DQB1*03:01	YLAGGVNSI	1.40	-0.23063	0.2364
KKVYLAGGVNSISKD (479)	DQA1*05:01-DQB1*03:01	YLAGGVNSI	1.40	-0.16456	0.0904
NIAIAMSGLTVLASA (5)	DQA1*05:01-DQB1*03:01	AMSGLTVLA	1.60	-0.12126	0.3174
	DQA1*01:02-DQB1*06:02		0.07		
LADAMSIAPVASQLK (539)	DQA1*05:01-DQB1*03:01	AMSIAPVAS	1.60	-0.3103	0.3181
	DQA1*01:02-DQB1*06:02		0.30		
GLADAMSIAPVASQL (538)	DQA1*05:01-DQB1*03:01	AMSIAPVAS	1.60	-0.23235	0.3436
	DQA1*01:02-DQB1*06:02		0.40		
AFVVGGTGLADAMSI (531)	DQA1*05:01-DQB1*03:01	FVVGGTGLA	1.60	0.03261	0.5150
KAFVVGGTGLADAMS (530)	DQA1*05:01-DQB1*03:01	FVVGGTGLA	1.60	0.23514	0.1430
ADAMSIAPVASQLKD (540)	DQA1*05:01-DQB1*03:01	AMSIAPVAS	1.80	-0.4954	0.3666
	DQA1*01:02-DQB1*06:02		0.60		
VQVGKGIASSVINKM (699)	DQA1*05:01-DQB1*03:01	VGKGIASSV	1.80	-0.24998	0.3923
FVVGGTGLADAMSIA (532)	DQA1*05:01-	GGTGLADAM	1.90	0.01114	0.4973

	DQB1*03:01				
VYLAGGVNSISKDVE (481)	DQA1*05:01-DQB1*03:01	YLAGGVNSI	1.90	-0.21709	0.4423
DKAFVVGGTGLADAM (529)	DQA1*05:01-DQB1*03:01	FVVGGTGLA	2.00	0.41531	-0.0202
KVGDSTAGIAINLPS (203)	DQA1*05:01-DQB1*03:01	STAGIAINL	2.00	0.25649	1.7093
GTNLVQVGKGIASSV (695)	DQA1*05:01-DQB1*03:01	VQVGKGIAS	2.00	-0.25951	0.1243
SGEDRYETSLAIADE (509)	DQA1*04:01-DQB1*04:02	ETSLAIADE	1.00	0.27438	0.7836
	DQA1*03:01-DQB1*03:02		0.40		
KEDQLVDALAAAPIA (647)	DQA1*04:01-DQB1*04:02	DQLVDALAA	1.40	0.15037	0.4338
	DQA1*03:01-DQB1*03:02		1.60		

Supplementary Table S6:

All the HTL epitopes of CotE as predicted by NetMHC II pan 3.2 where Sb indicates Strong binder

Epitopes(Sb)	HLA Alleles II	Core Sequence	Score	Immunogenicity	Antigenicity
MRIAKLYGMISKPMS (106)	DRB1*01:01	LYGMISKPM	1.20	-0.62629	-0.3207
	DRB1*08:02		1.60		
	DRB1*11:01	YGMISKPMS	1.30		
	DRB1*12:01	IAKLYGMIS	1.50		
	DRB1*15:01		1.10		
IPFYGRLGATITRTY (603)	DRB1*01:01	YGRLGATIT	1.40	0.44828	0.4240
RIAKLYGMISKPMSD (107)	DRB1*01:01	LYGMISKPM	1.40	-0.8535	-0.5809
	DRB1*08:01	YGMISKPMS	2.00		
	DRB1*11:01		1.40		
IAKLYGMISKPMSDT (108)	DRB1*01:01	LYGMISKPM	1.60	-0.76702	-0.5425
	DRB1*11:01	YGMISKPMS	1.30		
GIPFYGRLGATITRT (602)	DRB1*01:01	YGRLGATIT	1.70	0.48038	0.2872
RENFTLLLTAIRDVI (500)	DRB1*01:01	FTLLLTAIR	2.00	0.41816	-0.2621
	DRB1*04:05		1.00		
RSVFIIDNNQILRTI (126)	DRB1*03:01	FIIDNNQIL	0.10	0.47212	-0.5525
	DRB1*04:01		0.17		
	DRB1*04:05		1.20		
	DRB1*13:02		0.20		
	DRB3*01:01		0.01		
	DRB3*02:02		0.50		
VRSVFIIDNNQILRT (125)	DRB1*03:01	FIIDNNQIL	0.15	0.4034	-0.2115
	DRB1*04:01		0.25		
	DRB1*04:05		1.20		
	DRB1*13:02		0.25		
	DRB3*01:01		0.01		
	DRB3*02:02		0.60		
SVFIIDNNQILRTIL (127)	DRB1*03:01	FIIDNNQIL	0.20	0.47576	-0.4724
	DRB1*04:01		0.50		
	DRB1*13:02		0.30		
	DRB3*01:01		0.01		
	DRB3*02:02		0.70		
TVRSVFIIDNNQILR (124)	DRB1*03:01	FIIDNNQIL	0.40	0.26953	-0.1963
	DRB1*04:01		0.40		
	DRB1*04:05		1.60		
	DRB1*13:02		0.40		

	DRB3*01:01		0.01		
	DRB3*02:02		0.90		
VFIIDNNQILRTILY (128)	DRB1*03:01	FIIDNNQIL	0.40	0.3849	-0.2242
	DRB1*04:01		1.20		
	DRB1*13:02		0.50		
	DRB3*01:01		0.05		
	DRB3*02:02		0.90		
FPIIEDRDMRIAKLY (98)	DRB1*03:01	IIEDRDMRI	0.40	0.22557	0.8929
PFPIIEDRDMRIAKL (97)	DRB1*03:01	IIEDRDMRI	0.40	0.36272	1.0457
	DRB3*01:01		1.90		
IPFPIIEDRDMRIAK (96)	DRB1*03:01	IIEDRDMRI	0.60	0.49004	1.3625
AIRDVIGDDKWLSVA (509)	DRB1*03:01	VIGDDKWLS	0.70	0.16488	0.2627
IRDVIGDDKWLSVAG (510)	DRB1*03:01	VIGDDKWLS	0.70	0.15141	0.2317
PIIEDRDMRIAKLYG (99)	DRB1*03:01	IIEDRDMRI	1.00	0.07013	0.4478
EIPFPIIEDRDMRIA (95)	DRB1*03:01	IIEDRDMRI	1.00	0.50984	1.3352
RDVIGDDKWLSVAGT (511)	DRB1*03:01	VIGDDKWLS	1.10	0.149	0.3064
TAIRDVIGDDKWLSV (508)	DRB1*03:01	VIGDDKWLS	1.10	0.25537	0.3497
QVPYLVKDGDFAMSY (639)	DRB1*03:01	LVKDGDFA M	1.40	-0.20575	0.2371
	DRB3*01:01		0.90		
STVRSVFIIDNNQIL (123)	DRB1*03:01	FIIDNNQIL	1.60	0.30699	-0.2661
	DRB1*04:01		1.60		
	DRB1*13:02		1.00		
	DRB3*01:01		0.03		
VPYLVKDGDFAMSYD (640)	DRB1*03:01	LVKDGDFA M	1.50	-0.26094	0.3963
	DRB3*01:01		1.10		
RYNFARQVNQMINEY (460)	DRB1*04:01	FARQVNQMI	0.50	0.01982	0.3414
	DRB1*04:05		0.80		
SRYNFARQVNQMINE (459)	DRB1*04:01	FARQVNQMI	0.60	-0.05367	0.1556
	DRB1*04:05		0.90		
TSRYNFARQVNQMIN (458)	DRB1*04:01	FARQVNQMI	0.70	-0.0491	0.2860
	DRB1*04:05		1.10		
YNFARQVNQMINEYA (461)	DRB1*04:01	FARQVNQMI	1.10	-0.02908	0.1282
	DRB1*04:05		1.60		
GYEYRFDNTAQVPYL (629)	DRB1*04:01	YRFDNTAQV	1.50	0.19892	1.0370
	DRB3*01:01		0.20		
PTSRYNFARQVNQMI (457)	DRB1*04:01	FARQVNQMI	1.90	-0.08807	0.2970
YEYRFDNTAQVPYLV (630)	DRB1*04:01	YRFDNTAQV	2.00	0.16344	0.9228
	DRB3*01:01		0.30		
DRENFTLLLTAIRDV (499)	DRB1*04:05	FTLLLTAIR	1.10	0.40321	-0.1794
QDRENFTLLLTAIRD (498)	DRB1*04:05	FTLLLTAIR	1.70	0.44784	-0.1388
AKLYGMISKPMSDTS (109)	DRB1*11:01	YGMISKPMS	1.40	-0.68295	-0.2951
KLYGMISKPMSDTST (110)	DRB1*11:01	YGMISKPMS	1.90	-0.76552	-0.1464
NNQILRTILYYPLTT (133)	DRB1*12:01	LRTILYYPL	1.00	0.2882	0.4123
	DPA1*02:01-DPB1*01:01		1.90		
	DPA1*01:03-DPB1*02:01		1.80		
	DPA1*01:03-DPB1*04:01		1.60		
	DPA1*03:01-DPB1*04:02		1.80		
IDNNQILRTILYYPL (131)	DRB1*12:01	LRTILYYPL	1.00	0.19131	0.5054
DNNQILRTILYYPLT (132)	DRB1*12:01	LRTILYYPL	1.00	0.18422	0.3297
	DPA1*01:03-DPB1*04:01		2.00		
DMRIAKLYGMISKPM (105)	DRB1*12:01	IAKLYGMIS	1.50	-0.38819	0.2233
	DRB1*15:01		1.60		
NQILRTILYYPLTTG (134)	DRB1*12:01	IAKLYGMIS	1.60	0.26604	0.2708
RDMRIAKLYGMISKP (104)	DRB1*12:01	IAKLYGMIS	2.00	-0.33996	0.0895
NGYEYRFDNTAQVPY	DRB3*01:01	YRFDNTAQV	0.30	0.27087	1.0784

(628)					
GDFAMSYDDALSIFL (647)	DRB3*01:01	MSYDDALSI	0.40	-0.16284	0.2606
	DQA1*05:01 -DQB1*02:01	FAMSYDDAL	0.05		
	DQA1*01:01 -DQB1*05:01		0.08		
DFAMSYDDALSIFLK (648)	DRB3*01:01	MSYDDALSI	0.50	-0.2017	0.3804
	DQA1*05:01 -DQB1*02:01	FAMSYDDAL	0.12		
	DQA1*01:01 -DQB1*05:01		0.12		
FAMSYDDALSIFLKT (649)	DRB3*01:01	MSYDDALSI	0.50	-0.21323	0.3082
	DQA1*05:01 -DQB1*02:01	FAMSYDDAL	0.90		
	DQA1*01:01 -DQB1*05:01	AMSYDDALS	0.50		
KNGYEYRFDNTAQVP (627)	DRB3*01:01	YRFDNTAQV	0.50	0.28187	1.2654
DGDFAMSYDDALSIF (646)	DRB3*01:01	MSYDDALSI	0.60	-0.1636	0.1667
	DQA1*05:01 -DQB1*02:01	FAMSYDDAL	0.04		
	DQA1*01:01 -DQB1*05:01		0.12		
	DQA1*03:01 -DQB1*03:02		1.70		
	DQA1*04:01 -DQB1*04:02		2.00		
EYRFDNTAQVPYLVK (631)	DRB3*01:01	YRFDNTAQV	0.80	0.15368	0.4992
NKNGYEYRFDNTAQV (626)	DRB3*01:01	YRFDNTAQV	0.80	0.30388	1.5192
AMSYDDALSIFLKTQ (650)	DRB3*01:01	MSYDDALSI	1.10	-0.07418	0.0504
FIIDNNQILRTILYY (129)	DRB3*01:01	FIIDNNQIL	1.20	0.26088	-0.0346
KDGDFAMSYDDALSI (645)	DRB3*01:01	MSYDDALSI	1.30	-0.17627	0.5761
	DQA1*03:01 -DQB1*03:02	FAMSYDDAL	1.70		
	DQA1*01:01 -DQB1*05:01		0.40		
	DQA1*05:01 -DQB1*02:01		0.10		
PYLVKDGDFAMSYDD (641)	DRB3*01:01	LVKDGDFA M	1.50	-0.26065	0.4173
AQVPYLVKDGDFAMS (638)	DRB3*01:01	LVKDGDFA M	1.50	-0.06514	0.2561
MIYMPNLPSLGSKAP (1)	DRB3*02:02	YMPNLPSLG	1.00	-0.67037	0.9912
APDFKANTTNGPIRL (14)	DRB3*02:02	FKANTTNGP	1.60	0.18122	1.3231
PDFKANTTNGPIRLS (15)	DRB3*02:02	FKANTTNGP	1.90	0.10678	1.2789
SHLAWMYNISLLTGV (80)	DRB3*02:02	WMYNISLLT	2.00	0.07039	0.3897
LSIFLKTQYVLRNCL (657)	DPA1*02:01-DPB1*05:01	FLKTQYVLR	1.40	-0.09724	0.2874
DALSIFLKTQYVLRN (655)	DPA1*02:01-DPB1*05:01	FLKTQYVLR	1.50	-0.16613	0.0043
ALSIFLKTQYVLRNC (656)	DPA1*02:01-DPB1*05:01	FLKTQYVLR	1.70	-0.03552	0.3422
DDALSIFLKTQYVLR (654)	DPA1*02:01-DPB1*05:01	FLKTQYVLR	1.80	-0.18688	0.0640
FIPYPRFLNQLLALK (416)	DPA1*02:01-DPB1*05:01	FLNQLLALK	1.80	0.02168	0.6508
LLDAVIFAFAEIDQS (398)	DQA1*05:01 -DQB1*02:01	FAFAEIDQS	0.17	0.69439	0.2247
	DQA1*03:01 -DQB1*03:02	DAVIFAFAE	0.12		
	DQA1*04:01 -DQB1*04:02		0.12		

	DQA1*01:01 -DQB1*05:01	IFAFAEIDQ	0.10		
LDAVIFAFAEIDQSG (399)	DQA1*05:01 -DQB1*02:01	FAFAEIDQS	0.25	0.5416	0.5300
	DQA1*01:01 -DQB1*05:01	IFAFAEIDQ	1.40		
	DQA1*03:01 -DQB1*03:02	DAVIFAFAE	0.20		
	DQA1*04:01 -DQB1*04:02		0.20		
VKDGDFAMSYDDALS (644)	DQA1*05:01 -DQB1*02:01	FAMSYDDAL	0.40	-0.05168	0.6370
DAVIFAFAEIDQSGN (400)	DQA1*05:01 -DQB1*02:01	FAFAEIDQS	0.40	0.47136	0.4094
	DQA1*03:01 -DQB1*03:02		0.40		
	DQA1*04:01 -DQB1*04:02		0.40		
	DQA1*01:01 -DQB1*05:01	IFAFAEIDQ	1.80		
AVIFAFAEIDQSGNL (401)	DQA1*05:01 -DQB1*02:01	FAFAEIDQS	0.50	0.35619	0.1970
	DQA1*03:01 -DQB1*03:02		1.60		
	DQA1*04:01 -DQB1*04:02		1.50		
	DQA1*01:01 -DQB1*05:01	IFAFAEIDQ	2.00		
INEYALDGIDIDWEY (471)	DQA1*05:01 -DQB1*02:01	YALDGIDID	0.50	0.6578	1.3271
LVKDGDFAMSYDDAL (643)	DQA1*05:01 -DQB1*02:01	FAMSYDDAL	0.60	-0.11422	0.1840
NEYALDGIDIDWEYP (472)	DQA1*05:01 -DQB1*02:01	LDGIDIDWE	0.70	0.67522	1.1911
MINEYALDGIDIDWE (470)	DQA1*05:01 -DQB1*02:01	YALDGIDID	0.70	0.60157	1.1689
VIFAFAEIDQSGNLF (402)	DQA1*05:01 -DQB1*02:01	FAFAEIDQS	0.80	0.22423	-0.0366
QLLDAVIFAFAEIDQ (397)	DQA1*05:01 -DQB1*02:01	VIFAFAEID	0.90	0.75035	0.2105
	DQA1*01:01 -DQB1*05:01		1.60		
	DQA1*03:01 -DQB1*03:02	DAVIFAFAE	0.30		
	DQA1*04:01 -DQB1*04:02		0.40		
AQLLDAVIFAFAEID (396)	DQA1*05:01 -DQB1*02:01	VIFAFAEID	1.00	0.67993	0.4179
	DQA1*03:01 -DQB1*03:02	DAVIFAFAE	0.40		
	DQA1*04:01 -DQB1*04:02		0.40		
EYALDGIDIDWEYPG (473)	DQA1*05:01 -DQB1*02:01	LDGIDIDWE	1.10	0.65889	1.0531
QMINEYALDGIDIDW (469)	DQA1*05:01 -DQB1*02:01	YALDGIDID	1.40	0.48825	1.0917
VDAQLLDAVIFAFAE (394)	DQA1*03:01 -DQB1*03:02	DAVIFAFAE	0.50	0.37653	0.7625
	DQA1*04:01 -DQB1*04:02		0.80		
DAQLLDAVIFAFAEI (395)	DQA1*03:01 -DQB1*03:02	DAVIFAFAE	0.70	0.52376	0.5770
	DQA1*04:01		0.80		

	-DQB1*04:02				
KIAPIIDYFNLMSYD (539)	DQA1*01:01 -DQB1*05:01	IIDYFNLMS	0.50	0.06856	0.5955
DKIAPIIDYFNLMSY (538)	DQA1*01:01 -DQB1*05:01	IIDYFNLMS	0.50	0.20137	0.1975
IAPIIDYFNLMSYDF (540)	DQA1*01:01 -DQB1*05:01	IIDYFNLMS	0.60	0.05793	0.8677

Supplementary Table S7:

Overlapping HTL and CTL epitopes

**Epitopes (HTL)**	**Overlapping CTL Epitopes**	**Class I Supertypes and Alleles**	**Class II Alleles**
MRVNTNVSALIANNQ	RVNTNVSAL	B7, B62, HLA-B*07:02, HLA-A*32:01, HLA-A*02:03	DRB1*13:02, DRB3*02:02, DQA1*01:02-DQB1*06:02
	MRVNTNVSA	B27	
NNNEIKIQLVNTASI	IQLVNTASI	A24, B62, HLA-A*32:01, HLA-A*02:06, HLA-B*15:01	DRB1*04:01, DRB1*08:02, DRB1*13:02, DRB4*01:01
NNEIKIQLVNTASIM	IQLVNTASI	A24, B62, HLA-A*32:01, HLA-A*02:06, HLA-B*15:01	DRB1*04:01, DRB1*04:05, DRB1*08:02, DRB1*12:01, DRB1*13:02, DRB4*01:01
RYETSLAIADEIGLD	TSLAIADEI	HLA-B*58:01	DQA1*05:01-DQB1*02:01, DQA1*03:01-DQB1*03:02, DQA1*04:01-DQB1*04:02,
	YETSLAIAD	HLA-B*40:01	
ETSLAIADEIGLDND	TSLAIADEI	HLA-B*58:01	DQA1*05:01-DQB1*02:01, DQA1*03:01-DQB1*03:02, DQA1*04:01-DQB1*04:02
	LAIADEIGL	HLA-B*35:01	
IAGRFKESPAPIILA	KESPAPIIL	B44, B39, HLA-B*40:01,	DRB1*07:01, DRB1*09:01, DRB3*02:02, DPA1*02:01-DPB1*14:01
	GRFKESPAP	B27	
	RFKESPAPI	A24, HLA-A*30:01	
	ESPAPIILA	HLA-A*68:02	
	AGRFKESPA	HLA-A*30:01	
NNQILRTILYYPLTT	NQILRTILY	A1, A26, B27, HLA-B*15:01	DRB1*12:01, DPA1*02:01-DPB1*01:01, DPA1*01:03-DPB1*02:01, DPA1*01:03-DPB1*04:01, DPA1*03:01-DPB1*04:02
	QILRTILYY	A1, A3, A26, B58, B62, HLA-A*30:02, HLA-A*30:02, HLA-A*11:01	
	LRTILYYPL	B27, B39, B62	
	RTILYYPLT	HLA-A*30:01	
AQLLDAVIFAFAEID	AQLLDAVIF	B62, B44, HLA-B*15:01	DQA1*05:01-DQB1*02:01, DQA1*03:01-DQB1*03:02, DQA1*04:01-DQB1*04:02
	LLDAVIFAF	B62, B58, A2, HLA-A*02:01, HLA-A*02:06, HLA-B*15:01	
	QLLDAVIFA	HLA-A*02:01, HLA-A*02:06	
	AVIFAFAEI	HLA-A*02:06, HLA-A*68:02	
KDGDFAMSYDDALSI	FAMSYDDAL	A2, B8, B39, HLA-B*35:01, HLA-B*53:01, HLA-A*68:02	DRB3*01:01, DQA1*03:01-DQB1*03:02, DQA1*01:01-DQB1*05:01, DQA1*05:01-DQB1*02:01
	MSYDDALSI	B58, HLA-B*58:01, HLA-B*53:01	

Supplementary Table S8: Comparison of all the models predicted by trRosetta of the 6 vaccine constructs designed, based on ProSA, ERRAT and Ramachandran plot analysis. The chosen vaccine construct is italicized

S.no	Vaccine	ProSA	ERRAT	Ramachandran plot
Vaccine construct 1	Model 1	-8.77	39.68	93.9% in favoured region
	Model 2	-8.23	50.29	94.1% in favoured region
	Model 3	-8.05	39.68	94.5% in favoured region
	Model 4	-7.88	49.23	85.9% in favoured region
	Model 5	-8.24	45.69	93.5% in favoured region
Vaccine construct 2	Model 1	-6.67	49.00	94.9% in favoured region
	Model 2	-8.07	45.09	94.1% in favoured region
	Model 3	-7.57	42.6	95.7% in favoured region
	Model 4	-7.68	39.48	93.3% in favoured region
	Model 5	-8.37	44.89	92.5% in favoured region
Vaccine construct 3	Model 1	-7.12	38.29	88.3% in favoured region
	Model 2	-6.77	37.87	93.7% in favoured region
	Model 3	-7.79	38.69	96.9% in favoured region
	Model 4	-7.65	38.88	94.5% in favoured region
	Model 5	-7.89	35.119	95.9% in favoured region
Vaccine construct 4	Model 1	-7.87	41.31	96.6% in favoured region
	Model 2	***-8.92***	***53.38***	***96.5% in favoured region***
	Model 3	-8.74	43.57	94.3% in favoured region
	Model 4	-8.42	25.32	96.3% in favoured region
	Model 5	-8.05	40.55	96.5% in favoured region
Vaccine construct 5	Model 1	-7.93	48.90	95.1% in favoured region
	Model 2	-7.38	36.32	97.5% in favoured region
	Model 3	-8.5	45.83	96.1% in favoured region
	Model 4	-8.33	47	96.1% in favoured region
	Model 5	-8.24	39.68	94.3% in favoured region
Vaccine construct 6	Model 1	-8.16	44.84	93.6% in favoured region
	Model 2	-8.8	46.82	96.3% in favoured region
	Model 3	-9	50.11	93.9% in favoured region
	Model 4	-8.49	40.07	95.1% in favoured region
	Model 5	-7.94	40.45	92.9% in favoured region

Supplementary Table S9: IL2 inducing HTL epitopes, as predicted by IL2Pred server. All the IL-2 inducing HTL epitopes are shown in bold

Protein	IL-2 inducing HTL Epitopes		
	Sequence	Property	Confidence Score
FliC	**MRVNTNVSALIANNQ**	**IL-2 Inducers**	**0.63**
	**NNNEIKIQLVNTASI**	**IL-2 Inducers**	**0.518**
	**NNEIKIQLVNTASIM**	**IL-2 Inducers**	**0.506**
CotE	**NNQILRTILYYPLTT**	**IL-2 Inducers**	**0.822**
	AQLLDAVIFAFAEID	Non-IL-2 Inducers	0.378
	KDGDFAMSYDDALSI	Non-IL-2 Inducers	0.188
SlpA	**IAGRFKESPAPIILA**	**IL-2 Inducers**	**0.758**
	RYETSLAIADEIGLD	Non-IL-2 Inducers	0.452
	ETSLAIADEIGLDND	Non-IL-2 Inducers	0.368

Supplementary Table S10: Proinflammatory cytokine inducing HTL epitopes, as predicted by the ProInflam server. All the proinflammatory cytokine (includes TNF) inducing HTL epitopes are shown in bold

Protein	Epitopes	Score	Prediction
FliC	MRVNTNVSALIANNQ	-0.0186293	Negative
	NNNEIKIQLVNTASI	0.161698	Negative
	NNEIKIQLVNTASIM	0.194394	Negative
CotE	NNQILRTILYYPLTT	0.414755	Negative
	**AQLLDAVIFAFAEID**	**1.31402**	**Proinflamatory**
	**KDGDFAMSYDDALSI**	**0.950338**	**Proinflamatory**
SlpA	**RYETSLAIADEIGLD**	**0.862104**	**Proinflamatory**
	**ETSLAIADEIGLDND**	**0.763875**	**Proinflamatory**
	**IAGRFKESPAPIILA**	**0.867588**	**Proinflamatory**

Supplementary Table S11: IFN-γ inducing HTL epitopes as predicted by the IFNepitope server. A positive score indicates the epitopes to be IFN-γ inducing and all the IFN-γ inducing HTL epitopes are shown in bold

Protein	Epitopes	Prediction	Score
FliC	MRVNTNVSALIANNQ	NEGATIVE	-0.1317451
	NNNEIKIQLVNTASI	NEGATIVE	-0.28196038
	NNEIKIQLVNTASIM	NEGATIVE	-0.68297806
CotE	**NNQILRTILYYPLTT**	**POSITIVE**	**0.017958599**
	**AQLLDAVIFAFAEID**	**POSITIVE**	**0.095331273**
	KDGDFAMSYDDALSI	NEGATIVE	-0.69994113
SlpA	RYETSLAIADEIGLD	NEGATIVE	- 0.51675055
	ETSLAIADEIGLDND	NEGATIVE	- 0.63724713
	IAGRFKESPAPIILA	NEGATIVE	- 0.348843
